# Protective effect of sevoflurane on myocardial ischemia-reperfusion injury: a systematic review and meta-analysis

**DOI:** 10.1097/JS9.0000000000001975

**Published:** 2024-08-02

**Authors:** Amirhossein Nasiri-Valikboni, Mohamad Rashid, Amir Azimi, Hamed Zarei, Mahmoud Yousefifard

**Affiliations:** a Physiology Research Center, Iran University of Medical Sciences, Tehran; b Student Research Committee, Babol University of Medical Sciences, Babol, Iran

**Keywords:** anesthetics, inhalation, apoptosis, heart function tests, myocardial infarction, reperfusion injury, sevoflurane

## Abstract

**Background:**

Myocardial ischemia-reperfusion (I/R) injury significantly impacts recovery in both cardiac and noncardiac surgeries, potentially leading to severe cardiac dysfunction. Sevoflurane, a volatile anesthetic, is reputed for its protective effects against myocardial I/R injury, although evidence remains inconclusive. This systematic review and meta-analysis aim to clarify the cardioprotective efficacy of sevoflurane.

**Methods:**

The systematic search of databases including Medline, Embase, Scopus, and Web of Science, was supplemented with a manual search to retrieve studies using rat or mouse models of myocardial I/R injury, comparing sevoflurane pretreatment (≥24 h before I/R), preconditioning (within 24 h before I/R), or postconditioning (after I/R) against nontreated controls. The outcomes were cardiac function, myocardial infarct size, apoptosis, inflammation, oxidative stress, and cardiac biomarkers. Using the random effects model, standardized mean differences (SMD) were pooled to perform meta-analyses.

**Results:**

Fifty-one studies, encompassing 8189 subjects, were included in the meta-analysis. Pretreatment with Sevoflurane significantly reduced infarct size. Sevoflurane preconditioning exhibited positive effects on left ventricular parameters and ejection fraction, and reduced infarct size, apoptosis, and oxidative stress. Postconditioning with Sevoflurane demonstrated improvements in cardiac function, including enhanced left ventricular parameters and reduced infarct size, apoptosis, inflammation, oxidative stress, and cardiac biomarkers.

**Conclusion:**

Sevoflurane demonstrates a significant protective effect against myocardial I/R injury in animal models. These findings support the potential clinical utility of sevoflurane as an anesthetic choice in preventing and managing myocardial I/R injury during surgeries.

## Introduction

Highlights

- There is no conclusive evidence on cardioprotective effects of volatile anesthetics like sevoflurane in cardiac and noncardiac surgeries and their underlying mechanisms of action in secondary damages caused by myocardial reperfusion.
- Pretreatment with Sevoflurane significantly reduced infarct size.
- Sevoflurane preconditioning exhibited positive effects on left ventricular parameters and ejection fraction, and reduced infarct size, apoptosis, and oxidative stress.
- Postconditioning with Sevoflurane demonstrated improvements in cardiac function, including enhanced left ventricular parameters and reduced infarct size, apoptosis, inflammation, oxidative stress, and cardiac biomarkers.
- Sevoflurane demonstrates a significant protective effect against myocardial I/R injury in animal models.

Globally, 200 million people are estimated to suffer from ischemic heart disease^1^. This condition happens when myocardial oxygen demand outreaches its supply. The most common underlying process is coronary artery disease stemming from atherosclerotic plaque formation. When therapeutic interventions restore blood flow, the ischemic zone can suffer from secondary damage due to reperfusion injury, known as myocardial ischemia-reperfusion (I/R) injury^2^. The main underlying mechanisms for the development of I/R injury are not yet fully understood. Some key molecular mechanisms implicated in the progression of I/R injury include production of reactive oxygen species^3^, cardiomyocyte apoptosis^4^, inflammation, and oxidative stress^5^. There is no debates on the significance of myocardial I/R injury in cardiovascular surgeries, including cardiac bypass procedures, heart transplantation, and valve replacements^6^. Furthermore, following noncardiac surgeries, 17–20% of patients experience myocardial I/R injury^7,8^.

Volatile anesthetics are commonly employed for sustaining general anesthesia. They effectively inhibit patient reactions to surgical stimuli, while eliciting bronchodilation^9^. Patients undergoing anesthesia with volatile agents for on-pump coronary artery bypass graft surgeries exhibit lower postoperative troponin levels compared to those receiving intravenous anesthesia^10^. sevoflurane is a volatile, nonexplosive inhalational anesthetic agent that is rapidly absorbed and provides quick induction. It belongs to the halogenated ether drug class, along with other agents like isoflurane and desflurane. Some controversial evidence depicted among adults undergoing cardiac surgery with cardiopulmonary bypass, sevoflurane demonstrated superiority over propofol in terms of long-term mortality, myocardial infarction, cardiac index, the need for inotropic drugs, and extubation time^11^. Similar benefits have been identified in noncardiac surgeries^12^.

Volatile anesthetics such as sevoflurane may confer cardiac protection through diverse cellular pathways, including protein kinase C and G, ATP-dependent potassium channels, endothelial nitric-oxide (NO) synthetase, and the suppression of caspase-mediated apoptosis^13^. However, there is no conclusive evidence on cardioprotective effects of volatile anesthetics like sevoflurane in cardiac and noncardiac surgeries and their underlying mechanisms of action in secondary damages caused by myocardial reperfusion. This study aims to offer the highest level of evidence on the effectiveness of sevoflurane as a preventive and therapeutic agent on myocardial I/R injury through a systematic review and meta-analysis from all eligible preclinical trials. In addition to cardiac function as the primary outcome, we also evaluated some of the most important mechanisms of the cardioprotective effects of sevoflurane in myocardial I/R injury.

## Materials and methods

### Study design

This review adhered to the Preferred Reporting Items for Systematic Reviews and Meta-Analyses (PRISMA 2020, Supplemental Digital Content 1, http://links.lww.com/JS9/D229, Supplemental Digital Content 2, http://links.lww.com/JS9/D230) guidelines (Supplementary Table 1, Supplemental Digital Content 1, http://links.lww.com/JS9/D229)^14^ and was prospectively registered on the International Prospective Register of Systematic Reviews (PROSPERO ID: CRD42023454464). The overall quality of the review was assessed as high quality using the AMSTAR-2 criteria (Supplemental Digital Content 3, http://links.lww.com/JS9/D231)^15^.

Several methods were utilized to choose appropriate keywords. These methods encompassed MeSH terms (in Medline), Emtree terms (in Embase), and their synonyms, along with consultation with subject matter experts and an overview of related articles aided in keyword selection. Subsequently, a thorough search was conducted across electronic databases, including Medline (via PubMed), Embase, Scopus, and Web of Science, by 9th August 2023, to identify relevant articles. Search strategies were based on keywords related to sevoflurane and myocardial I/R injury (Supplementary Material 1, Supplemental Digital Content 1, http://links.lww.com/JS9/D229). An additional manual search was carried out in Google and Google Scholar. Furthermore, the references of review articles and included articles were searched for relevant studies.

### Inclusion and exclusion criteria

The definition of PICO was as follows: The population (P) of the study was animal (rats or mice) models of myocardial I/R injury without risk factors of cardiovascular disease (i.e. diabetes, aging, hyperlipidemia, or obesity). The intervention (I) was the administration of sevoflurane. The comparison (C) was made with a control group (myocardial I/R injury with no treatment). The primary outcome (O) was cardiac function and the secondary outcomes were myocardial infarct size, apoptosis, inflammation, oxidative stress, and serum levels of cardiac biomarkers. Accordingly, we excluded studies on animals other than rat and mice, combination therapy, human studies, in-vitro studies, diabetic animals, and studies that had not reported the required data for meta-analysis of any desired outcomes.

### Data gathering

The results of the systematic search were imported into the X8.0 version of the Endnote software and duplicate reports were removed. During the initial screening, two researchers worked independently to evaluate the titles and abstracts of the identified articles and identify those that could potentially be relevant to the study. Subsequently, the full texts of these selected articles underwent a thorough review, during which the inclusion and exclusion criteria were applied. Articles that met these criteria were included in the study.

The obtained data included information regarding the bibliographic information of the articles, the sample size and animals’ characteristics, the I/R injury induction model, duration of ischemia, anesthesia agent, drug dose, administration method, duration of exposure with sevoflurane, repeat of treatment, interval of injury to treatment, the interval of injury to outcome assessment (follow-up), and the treatment setting. If multiple follow-ups were reported for the outcome measurement, we used the data from the longest follow-up in the meta-analysis. There were three types of therapeutic settings among studies. Pretreatment means the administration of sevoflurane at least 24 h before I/R injury. If sevoflurane was administered within 24 h before injury and after injury, it was defined as preconditioning and postconditioning, respectively.

For each eligible study, in cases the required data was only presented in figures, two reviewers (A.N. and M.R.) used the Plot Digitizer software to gather data from statistical graphs independently. Then, the mean values were adopted. Any discrepancies were effectively addressed through constructive discussions involving a third reviewer.

### Outcomes

In our study, we assessed a comprehensive array of cardiac function outcomes to evaluate the impact of sevoflurane conditionings on heart performance and structure. These outcomes included the maximal decline in left ventricular pressure (negative dp/dt), the maximal rate of increase in left ventricular pressure (positive dp/dt), left ventricular ejection fraction (LVEF), left ventricular end-diastolic pressure (LVEDP), left ventricular systolic pressure (LVSP), left ventricular internal diameter at end-diastole (LVIDd) and end-systole (LVIDs), left ventricular fractional shortening (LVFS), and heart rate (HR). Myocardial infarct size was quantified using Evans blue dye and Triphenyltetrazolium chloride (TTC) double staining. Apoptosis was evaluated by counting TUNEL-positive cells and measuring the expression levels of B-cell lymphoma 2 (Bcl-2) and caspase-3. Additionally, the inflammatory response was assessed by quantifying cytokines such as interleukin-1 beta (IL-1β), interleukin-6 (IL-6), and tumor necrosis factor alpha (TNF-α). Oxidative stress was examined through the levels of glutathione (GSH), malondialdehyde (MDA), and superoxide dismutase (SOD). Finally, cardiac biomarkers including creatine kinase-myocardial band (CK-MB) and lactate dehydrogenase (LDH) were measured to evaluate cellular injury and myocardial stress.

### Risk of bias assessment and certainty of evidence

The quality control of the included studies were evaluated using SYRCLE’s risk of bias assessment tool^16^. For evaluation of the certainty of evidence for each outcome, we used the Grading of Recommendations Assessment, Development, and Evaluation (GRADE)^17^. Any conflicts or disagreements were resolved through discussions with a third researcher.

### Statistical analyses

The statistical analyses for this study were carried out utilizing STATA 17.0. The included studies were classified and summarized according to administration time of sevoflurane; which was categorized as pretreatment, preconditioning, and postconditioning. These classifications were analyzed and will be reported separately.

For each individual experiment, we computed a standardized mean difference (SMD) by Hedge’s g method, along with a 95% CI. These SMD values were then combined to determine an overall effect size. A meta-analysis was conducted only when data were available from a minimum of three distinct studies. In the present study, the random effect model was selected as the analytical approach, primarily because methodological heterogeneity was observed in most of the analyses. This model accounts for variations among the included studies and provides a more conservative estimation of the overall effect size in the presence of such heterogeneity.

To evaluate the statistical heterogeneity, we visually inspected the forest plots and employed *I* ^2^ and *χ* ^2^ tests. Additionally, when there was a minimum of 10 experiments related to a particular outcome, we conducted subgroup analyses and meta-regression to pinpoint potential factors contributing to this heterogeneity.

Subgroup analyses were performed on different animal species, I/R models, ischemia duration, injury to treatment interval, sevoflurane dose, and treatment duration. Additionally, for outcomes with at least 10 experiments, we reported the publication bias assessment with a Funnel Plot using the modified Egger’s test suggested by Doleman *et al*.^18^.

## Results

### Study selection and characteristics of the included studies

**FIGURE 1:**
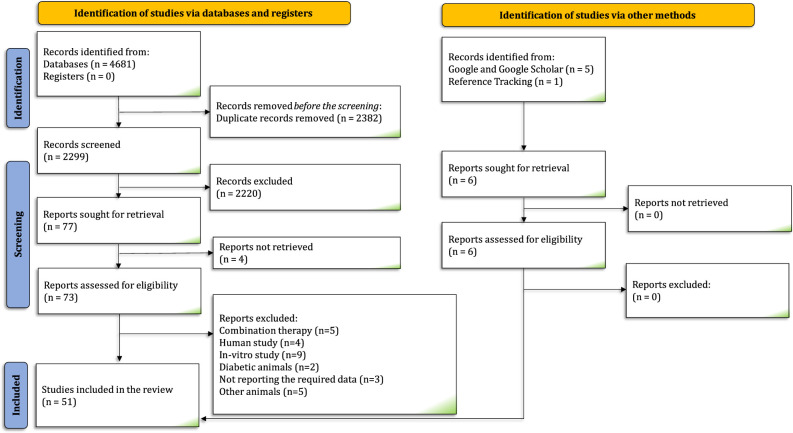
The PRISMA flow diagram depicts the flow of the study selection process through the different phases of the present systematic review.

Systematic search in electronic databases resulted 2299 nonduplicated studies. Following the screening of titles and abstracts and the manual search, the full-text of 79 articles were reviewed in details. Subsequently, 51 studies were included in the meta-analysis^19–69^. The PRISMA flowchart and selection methods are presented in Figure 1. Characteristics of the included studies are presented in Table 1. Gathered data from 8189 animals (4067 in the control group and 4122 in the treatment group) were pooled and analyzed separately in three groups based on the treatment setting of sevoflurane; pretreatment, preconditioning, and postconditioning. Left anterior descending artery (LAD)/left main coronary artery (LMCA) ligation was the most commonly used I/R induction model, followed by Langendorf model. In pretreatment experiments, sevoflurane was administered at 2.4–2.5% concentration. The corresponding values for preconditioning and postconditioning experiments are 1.8 to 3.6%, and 1 to 4.8%, respectively. The interval between injury and treatment was 24–168 h for pretreatment, 0–75 min for preconditioning, and 25–60 min for postconditioning experiments. sevoflurane administration was single-dose or double-dose in pretreatment, one to six doses in preconditioning, and single-dose in all postconditioning experiments. The animals were exposed by sevoflurane for 60–120 min in pretreatment, 5–45 min in preconditioning, and 2–18 min in postconditioning experiments. In pretreatment experiments, the outcomes were assessed in 120 min postinjury in almost all experiments, except for one experiment which measured TUNEL-positive cells after one day. The corresponding range was 60 min to 4 days for preconditioning, and 60 min to 7 days for postconditioning of sevoflurane.

**Table 1:** Summary characteristics of the included studies.

Author, year, country	Sex, strain, species, age (weeks), weight (gram),	Number of controls/treated	I/R model, ischemia duration (min)	Treatment condition, anesthetic drug	sevoflurane dose, administration route	Treatment duration (min)
An, 2023^19^, China	Male, Sprague Dawley, Rat, Adult, 220-250	12/12	LAD, 30	Precondition, 3% Pentobarbital sodium (50 mg/kg)	2.4%, Inhalation	10 min
Bahrenburg, 2017^20^, Germany	Male, Wistar, Rat, Adult, 268-340	8/8	LAD, 25	Pretreatment, Pentobarbital sodium (100 mg/kg)	1 MAC, Inhalation	60, 120 min
Cao, 2015^21^, China	Male, Sprague Dawley, Rat, 9-10, 270-350	6/6	Langendorff, 30	Postcondition, Pentobarbital sodium (50 mg/kg)	2.5%, Inhalation	15 min
Deng, 2022^22^, China	Male, C57/BL6, Mice, NR, 20-25	5/5	LAD, 30	Postcondition, Pentobarbital sodium (50 mg/kg)	2.5%, Inhalation	2 min
Drenger, 2011^23^, USA	Male, Sprague Dawley, Rat, 12, 310-340	10/8	LMCA, 30	Postcondition, Ketamine and xylazine	2.4%, Inhalation	5 min
Frassdorf, 2010^24^, Netherlands	Male, Wistar, Rat, NR, 380-420	6/6	LMCA, 25	Precondition, ketamine (150 mg/kg BW) intraperitoneal Injection	2.4%, Inhalation	5, 10, 15, 30 min
Gao, 2016^26^, China	Male, C57BL/6, Mice, 7-8, NR	7/7	LAD, 45	Postcondition, Pentobarbital sodium (30 mg/kg)	2%, Inhalation	15 min
Gao, 2021^25^, China	Male, C57BL/6, Mice, 7-8, NR	7/7	LAD, 45	Postcondition, NR	2%, Inhalation	15 min
Geng, 2022, China	Male, Sprague Dawley, Rat, Adult, 250-300	10/10	Langendorff, 30	Postcondition, Chloral hydrate	3%, Perfused	15 min
Hong, 2020^27^, China	Male, Sprague Dawley, Rat, 10-12, 230-330	8-10/8-10	LAD, 30	Pretreatment, Pentobarbital sodium (100 mg/kg)	2.4%, Inhalation	120 min
Huang, 2019^28^, China	Male, C57BL/6, Mice, 10-12, 20-30	10/15	LMCA, 30	Postcondition, Pentobarbital sodium (50 mg/kg)	2.4%, Inhalation	5 min
Huhn, 2008^29^, Netherlands	Male, Wistar, Rat, NR, 250-350	9/9-11	LAD, 25	Postcondition, S-ketamine (150 mg/kg)	2.4% and 4.8%, Inhalation	5 min
Li, 2013^31^, China	Male, Sprague Dawley, Rat, 12-96, 325-559	6/6	LAD, 30	Postcondition, Pentobarbital sodium (30–40 mg/kg)	3%, Inhalation	5 min
Li, 2015, China	Male, Sprague Dawley, Rat, 12-96, 325-559	12/12	LAD, 30	Postcondition, Pentobarbital sodium (30–40 mg/kg)	1and 2 MAC, Inhalation	5 min
Lin, 2016^32^, China	Male, Sprague Dawley, Rat, NR, 300-350	12/12	LAD, 30	Postcondition, Pentobarbital sodium (50 mg/kg)	2%, Inhalation	15 min
Liu, 2019^33^, China	Male, Sprague Dawley, Rat, 6, 200-220	12/12	LAD, 30	Precondition, 1% Pentobarbital sodium (50 mg/kg)	2.4%, Inhalation	Three episodes of 5 min
Ma, 2013^34^, China	Male, Sprague Dawley, Rat, NR, 130-180	8/8	LMCA, 30	Postcondition, Pentobarbital sodium (50 mg/kg)	2.4%, Inhalation	Three episodes of 5 min
Obal, 2001^36^, Germany	NR, Wistar, Rat, NR, 479-493	11/11	LAD then modify Langendorff, 25	Postcondition, Pentobarbital sodium (60 mg/kg)	1.8% and 2.4% and 3.6% and 4.8%, Inhalation	15 min
Obal, 2003^37^, Germany	NR, Wistar, Rat, NR, 477-503	7/7-8	LAD then modify Langendorff, 25	Postcondition, Chloralose	2.4%, Inhalation	2, 5, 10 min
Obal, 2005^35^, Germany	Male, Wistar, Rat, NR, 351-443	9/10	LAD, 25	Pre/Postcondition, a-chloralose	2%, Inhalation	2 min, Two episodes of 5 min
Popescu, 2022^38^, Romania	Male, Wistar, Rat, 20-28, 300-400	3/3	LAD, 30	Pretreatment, Chloral hydrate solution (0.8 ml/100 g)	2.4%, Inhalation	Cycles of 5 min each (30 min)
Qi, 2019^39^, China	Male, C57BL/6, Mice, NR, NR	20/20	LAD, 30	Postcondition, Ketamine and xylazine	3.4%, Inhalation	5 min
Qiao, 2012, China	Male, Sprague Dawley, Rat, 9-10, 270-320	6/6	LAD, 30	Pretreatment, Pentobarbital sodium (50 mg/kg)	2.5%, Inhalation	120 min
Qiao, 2018, China	Male, Sprague Dawley, Rat, 9-12, 250-350	8/8	LAD/Langendorff, 30	Postcondition, Pentobarbital sodium (50 mg/kg)	2.5%, Inhalation	15 min
Qin, 2022^68^, China	Male, Sprague Dawley, Rat, Adult, 250-300	6/6	LAD, 30	Precondition, Pentobarbital sodium (50 mg/kg)	2.5%, Inhalation	30 min
Redel, 2006, Germany	Male, C57BL/6, Mice, 8-12, NR	8/8	LAD, 45	Pre/Post-condition, Pentobarbital sodium (50 mg/kg)	1 MAC, Inhalation	15,18 min
Rui, 2021^42^, China	Male, C57BL/6N, Mice, 6-8, 15-25	3/3	LAD, 30	Postcondition, Pentobarbital sodium at 60 mg/kg and with atropine at 0.04 mg/kg	2.4%, Inhalation	15 min
Song, 2022^43^, China	Male, Sprague Dawley, Rat, 8, 250-300	18/18	LMCA, 30	Postcondition, 1% Pentobarbital sodium (50 mg/kg)	2.4%, Inhalation	15 min
Stumpner, 2014^44^, Germany	Male, C57BL/6, Mice, 8-12, NR	7/7	LAD, 45	Postcondition, Pentobarbital sodium (50 mg/kg)	1 MAC, Inhalation	15 min
Tai, 2012^45^, China	Male, Sprague Dawley, Rat, NR, 250-300	7/7	LAD, 30	Postcondition, Pentobarbital sodium (50 mg/kg)	1 MAC, Inhalation	5 min
Tan, 2020^46^, China	Male, Sprague Dawley, Rat, 7-8, 200-300	6-8/6-8	LAD, 30	Postcondition, 3% Pentobarbital sodium (50 mg/kg)	1% and 2% and 4%, Inhalation	5 min
Tosaka, 2011^47^, Japan	Male, Sprague Dawley, Rat, 13-15, 422-488	7/7	LAD, 30	Pre/Post-condition, Pentobarbital sodium (50 mg/kg)	2%, Inhalation	8, 15 min
Wang, 2010^48^, China	Male, Sprague Dawley, Rat, NR, 270-350	8/8	LAD, 30	Precondition, Pentobarbital sodium (50 mg/kg)	2.5%, Inhalation	30 min
Wang, 2020, China	Male, C57BL/6, Mice, 8-10, 25-30	10/10	LAD, 30	Precondition, 0.8% Pentobarbital sodium (50 mg/kg)	2.5%, Inhalation	15 min
Wu, 2021^50^, Chia	Male, Sprague Dawley, Rat, 8, 250-300	6/6	LAD, 45	Precondition, 1% Pentobarbital sodium	1.8% and 2.4% and 3.6%, Inhalation	45 min
Wu, 2022^51^, China	Male, Sprague Dawley, Rat, 8-12, 200-300	10/10	LAD, 30	Postcondition, 2.5% Pentobarbital sodium (50 mg/kg)	3.4%, Inhalation	5 min
Xiao, 2011^52^, China	Male, Sprague Dawley, Rat, Adult, 250-300	6/6	LAD, 30	Pretreatment, Pentobarbital sodium (50 mg/kg)	2.5%, Inhalation	60 min
Xie, 2014^54^, China	Male, Sprague Dawley, Rat, NR, 270-350	8/8	LAD, 30	Pretreatment, Pentobarbital sodium (50 mg/kg)	2.5%, Inhalation	120 min
Xie, 2020^53^, China	Male, C57BL/6 J or AMPK-DN or WT, Mice, 8-10, NR	6-15/6-15	LAD, 30	Precondition, Isoflurane	2%, Inhalation	Three episodes of 5 min
Xu, 2013^55^, China	Male, Sprague Dawley, Rat, 6, 130-180	8/8	LAD, 30	Postcondition, Pentobarbital sodium (50 mg/kg)	2.4%, Inhalation	5 min
Yang, 2016^56^, China	Male, Sprague Dawley, Rat, Adult, 250-300	22/22	Langendorff, 45	Postcondition, Pentobarbital sodium (40 mg/kg)	1 MAC, Perfused	15 min
Yao, 2010^57^, China	Male, Sprague Dawley, Rat, Adult, 250-300	15/15	Langendorff, 30	Postcondition, Pentobarbital sodium (40 mg/kg)	3%, Perfused	15 min
Yu, 2014, China	Male, Sprague Dawley, Rat, Adult, 200-230	10/10	LAD, 30	Postcondition, Pentobarbital sodium (50 mg/kg)	2.4%, Inhalation	15 min
Yu, 2021^58^, China	Male, Sprague Dawley, Rat, Adult, 220-250	8/8	LAD, 30	Postcondition, 25% urethane (5 ml/ kg intraperitoneally)	2.5%, Inhalation	10 min
Zeng, 2022, Pakistan	Male, Sprague Dawley, Rat, 8-10, 280-300	7/7	LAD, 30	Postcondition, Pentobarbital sodium (50 mg/kg)	NR, Inhalation	15 min
Zhang, 2012^61^, China	Male, Sprague Dawley, Rat, NR, 130-180	6/6	LAD, 30	Pretreatment, Chloral hydrate	2.4%, Inhalation	60 min
Zhang^62^, 2014, China	Male, Sprague Dawley, Rat, Adult, 180-230	9-10/9-10	LAD/ Langendorff, 30	Postcondition, Pentobarbital sodium (50 mg/kg)	2.4%, Inhalation/ Perfused	15 min
Zhang, 2020, China	Male, Sprague Dawley, Rat, Adult, 180-230	8/8	LAD/ Langendorff, 30	Postcondition, Pentobarbital sodium (50 mg/kg)	2.4%, Inhalation/ Perfused	15 min
Zhang^64^, 2022, China	Male, WT C57BL/6, Mice, Adult, 20-25	12/12	LAD, 30	Precondition, Isoflurane	2%, Inhalation	Three cycles of 15 min
Zhao, 2013^65^, China	Male, WT or AMPK-DN, Mice, 6-7, NR	14-16/14-16	LAD, 30	Precondition, 2% Isoflurane	2%, Inhalation	Three episodes of 10 min
Zhou, 2017^66^, China	Male, Sprague Dawley, Rat, Adult, 250-300	10/10	Langendorff, 30	Pre/Post-condition, Nembutal sodium (50 mg/kg)	1 MAC, Perfused	10 min

AMPK-DN, AMP-activated Protein Kinase Dominant Negative; I/R, Ischemia/Reperfusion; KO, knockout; LAD, left anterior descending; LMCA, left main coronary artery; MAC, minimum alveolar concentration; NR, not reported; WT, wild-type.

### Meta-analysis

#### Effect of pretreatment administration of sevoflurane on myocardial I/R injury

Pretreatment administration of sevoflurane was defined as the administration of sevoflurane 24 h or more prior to I/R injury. Seven studies including 34 analyses were included in the meta-analysis of the therapeutic effects of sevoflurane pretreatment in myocardial I/R injury. The interval between pretreatment administration of sevoflurane and myocardial I/R injury varied between 24 h and 7 days. The findings of this section are presented in Table 2. Assessment of publication bias was only applicable for infarct size, which showed no evidence of publication bias in this part of the study (*P*=0.404, Supplementary Figure 1, Supplemental Digital Content 1, http://links.lww.com/JS9/D229).

- Cardiac function
  Pretreatment with sevoflurane was not associated with a significant change in HR following myocardial I/R injury (SMD=0.12, 95% CI: −0.35 to 0.59, *P*=0.626; *I*²=0.00%) (Table 2, Supplementary Figure 2, Supplemental Digital Content 1, http://links.lww.com/JS9/D229). We could not assess the effect of pretreatment administration of sevoflurane on other cardiac function parameters due to the insufficient number of studies. Assessing the level of evidence indicated a moderate level of evidence using the GRADE framework (Supplementary Table 2, Supplemental Digital Content 1, http://links.lww.com/JS9/D229).
- Myocardial infarct size

**Table 2:** The efficacy of sevoflurane pretreatment and preconditioning in myocardial ischemia/reperfusion injury.

Outcome	Number of experiments	*P* for publication bias	*I* ^2^ (*P*)	SMD (95% CI)	*P*	Level of evidence^a^
Pretreatment						
Cardiac function						
HR	4	NA	0.00% (0.902)	0.12 (−0.35, 0.59)	0.626	Moderate
Infarct size	14	0.404	71.98% (<0.0001)	-2.02 (−2.65, −1.40)	<0.0001	High
Preconditioning						
Cardiac function						
-dp/dt	4	NA	0.00% (0.714)	2.02 (1.50–2.53)	<0.0001	High
+dp/dt	4	NA	65.60% (0.030)	2.59 (1.61–3.58)	<0.0001	High
LVEDP	4	NA	0.00% (0.485)	−0.99 (−1.41, −0.57)	<0.0001	High
LVEF	6	NA	92.06% (0.0001)	3.07 (1.21–4.93)	0.001	Moderate
HR	9	NA	70.35% (0.002)	−0.22 (−0.84, 0.39)	0.475	Moderate
Infarct size	17	0.098	79.14% (<0.0001)	−3.42 (−4.18, −2.65)	<0.0001	Moderate
Apoptosis						
TUNEL-positive cells	11	0.897	82.09% (<0.0001)	−3.47 (−4.59, −2.34)	<0.0001	Moderate
Caspase-3	5	NA	91.32% (0.0002)	−2.40 (−4.08, −0.72)	0.005	Moderate
Oxidative stress						
MDA	5	NA	97.02% (<0.0001)	−7.04 (−12.49, −1.58)	0.012	Moderate
SOD	5	NA	95.53% (<0.0001)	17.16 (6.86–27.46)	0.001	Moderate
Cardiac biomarkers						
Troponin-I	4	NA	67.26% (0.044)	−1.65 (−2.53, −0.78)	<0.0001	Moderate
LDH	5	NA	89.68% (<0.0001)	−4.85 (−7.42, −2.28)	<0.0001	Moderate

^a^ Based on the GRADE framework. The details are presented in Supplementary Table 1 (Supplemental Digital Content 1, http://links.lww.com/JS9/D229).

+dp/dt, Maximal rate of left ventricular pressure; -dp/dt, Maximal decline in left ventricular pressure; HR, heart rate; LDH, lactate dehydrogenase; LVEDP, left ventricular end-diastolic pressure; LVEF, left ventricular ejection fraction; MDA, malondialdehyde; NA, not applicable due to the insufficient number of experiments; SMD, standardize mean difference; SOD, superoxide dismutase.

Pretreatment with sevoflurane significantly reduces the infarct size following myocardial I/R injury (SMD=−2.02, 95% CI: −2.65 to −1.40, *P*<0.0001; *I*²=71.98%) (Table 2, Supplementary Figure 2, Supplemental Digital Content 1, http://links.lww.com/JS9/D229).

Subgroup analysis and meta-regression were performed to investigate the source of heterogeneity. The improvement in infarct size was evident in all subgroups. However, meta-regressions demonstrated that the extent of improvement is significantly greater in animals with a 30 min ischemia duration compared to 25 min (meta-regression coefficient=−1.44, 95% CI: −2.54 to −0.35; *P*=0.010) (Table 3). Moreover, meta-regression showed that sevoflurane pretreatment has a better ameliorating effect on infarct size when administered 24 h before myocardial I/R injury compared to longer intervals of treatment and injury (meta-regression coefficient=−1.51, 95% CI: −2.54 to −0.48; *P*=0.004) (Table 3). There was also no dose-dependent effect of sevoflurane pretreatment on the infarct size (meta-regression coefficient=−9.50, 95% CI: −23.24 to 4.24; *P*=0.175) (Table 3). The level of evidence was indicated high for infarct size, using the GRADE framework (Supplementary Table 2, Supplemental Digital Content 1, http://links.lww.com/JS9/D229).

**Table 3:** Subgroup analyses and meta-regressions for different variables in pretreatment administration of sevoflurane.

		Subgroup analysis			Meta-regression	
	Number of experiments	SMD [95% CI]	*P*	*I* ^2^ (%)	Coefficient [95% CI]	*P*
Infarct size						
Animal						
Mice	0	N/A			N/A	
Rat	14	−2.02 [−2.65, −1.40]	<0.0001	71.98		
I/R model						
Langendorf	0	N/A			N/A	
LAD/LMCA ligation	14	−2.02 [−2.65, −1.40]	<0.0001	71.98		
Ischemia duration						
25 min	8	−1.47 [−2.26, −0.67]	<0.0001	75.74	Reference	
30 min	6	−2.86 [−3.46, −2.26]	<0.0001	0.00	−1.44 [−2.54, −0.35]	0.010
Treatment to ischemia interval						
Longer than 24 h	7	−1.34 [−2.19, −0.48]	0.002	76.78	Reference	
Exactly 24 h	7	−2.79 [−3.33, −2.25]	<0.0001	0.00	−1.51 [−2.54, −0.48]	0.004
Frequency of administration						
1	10	−2.20 [−2.96, −1.44]	<0.0001	70.85	Reference	
2	4	−1.61 [−2.77, −0.46]	<0.0001	76.27	0.59 [−0.79, 1.97]	0.400
Treatment duration						
60 min	6	−1.87 [−2.97, −0.77]	0.001	79.15	Reference	
120 min	8	−2.15 [−2.92, −1.38]	<0.0001	66.99	−0.32 [−1.62, 0.97]	0.623
Sevoflurane dose						
2.4%	10	−1.79 [−2.56, −1.01]	<0.0001	77.36	Reference	
2.5%	4	−2.70 [−3.42, −1.97]	<0.0001	0.00	−0.95 [−2.32, 0.42]	0.175

#### Effect of sevoflurane preconditioning on myocardial I/R injury

Preconditioning administration of sevoflurane was defined as the administration of sevoflurane less than 24 h prior to I/R injury. Sixteen studies including 114 analyses were included in the meta-analysis of the therapeutic effect of sevoflurane preconditioning in myocardial I/R injury. The findings of this section are presented in Table 2. Assessment of publication bias was only applicable for infarct size and TUNEL-positive cells, which showed none in this part of the study (*P*=0.098 and *P*=0.897, respectively; Supplementary Figure 3, Supplemental Digital Content 1, http://links.lww.com/JS9/D229).

- Cardiac function
  The meta-analysis demonstrated that animals treated with sevoflurane preconditioning had a significant increase in negative dp/dt (SMD=2.02, 95% CI: 1.50–2.53, *P*<0.0001; *I*²=0.00%) and positive dp/dt (SMD=2.59, 95% CI: 1.61–3.58, *P*<0.0001; *I*²=65.60%) (Table 2, Supplementary Figure 4, Supplemental Digital Content 1, http://links.lww.com/JS9/D229). Sevoflurane preconditioning could also raise LVEF (SMD=3.07, 95% CI: 1.21–4.93, *P*=0.001; *I*²=92.06%) and reduce LVEDP (SMD=−0.99, 95% CI: −1.41 to −0.57, *P*<0.0001; *I*²=0.00%) (Table 2, Supplementary Figure 4, Supplemental Digital Content 1, http://links.lww.com/JS9/D229). However, sevoflurane preconditioning did not have a significant effect on the HR (SMD=−0.22, 95% CI: −0.84 to 0.39, *P*=0.475; *I*²=70.35%) (Table 2, Supplementary Figure 4, Supplemental Digital Content 1, http://links.lww.com/JS9/D229). We evaluated the level of evidence for the therapeutic effect of sevoflurane preconditioning as high for -dp/dt, +dp/dt, and LVEDP. However, the level of evidence for LVEF and HR were moderate (Supplementary Table 2, Supplemental Digital Content 1, http://links.lww.com/JS9/D229).
- Myocardial infarct size
  The meta-analysis demonstrated that sevoflurane preconditioning significantly declined myocardial infarct size (SMD=−3.42, 95% CI: −4.18 to −2.65, *P*<0.0001; *I*²=79.14%) (Table 2, Supplementary Figure 5, Supplemental Digital Content 1, http://links.lww.com/JS9/D229).
  The improvement in infarct size was evident in all subgroups. In addition, meta-regressions demonstrated that the extent of improvement was not statistically different in any of the subgroups (Table 4). Meta-regression also showed that the effect size is not dependent to sevoflurane dose (meta-regression coefficient=0.11, 95% CI: −1.94 to 2.15; *P*=0.919) (Table 4). Therefore, we cannot find the source of heterogeneity. Subsequently, the level of evidence was rated down to moderate (Supplementary Table 2, Supplemental Digital Content 1, http://links.lww.com/JS9/D229).
- Apoptosis
  The meta-analysis demonstrated that sevoflurane preconditioning significantly subsided the number of TUNEL-positive cells (SMD=−3.47, 95% CI: −4.59 to −2.34, *P*<0.0001; *I*²=65.60%, *P*=0.030) and caspase-3 levels (SMD=−2.40, 95% CI: −4.08 to −0.72, *P*=0.005; *I*²=91.32%, *P*=0.0002) in the myocardium after I/R injury (Table 2, Supplementary Figure 6, Supplemental Digital Content 1, http://links.lww.com/JS9/D229). Both of these effects are in favor of supressing apoptosis.
  The reduction of TUNEL-positive cells was evident in all subgroups. Meta-regressions demonstrated that the extent of improvement was not statistically different in any of the subgroups (Table 4). The level of evidence is moderate for the effect of sevoflurane preconditioning on apoptosis (Supplementary Table 2, Supplemental Digital Content 1, http://links.lww.com/JS9/D229).
- Oxidative stress
  The meta-analysis demonstrated that sevoflurane preconditioning could significantly reduce MDA content (SMD=−7.04, 95% CI: −12.49 to −1.58, *P*=0.012; *I*²=97.02%, *P*<0.0001) (Table 2, Supplementary Figure 7A, Supplemental Digital Content 1, http://links.lww.com/JS9/D229). On the other hand, it increased SOD in treated animals compared to the nontreated group (SMD=17.16, 95% CI: 6.86–27.46, *P*=0.001; *I*²=95.53%, *P*<0.0001) (Table 2, Supplementary Figure 7A, Supplemental Digital Content 1, http://links.lww.com/JS9/D229). Both of these effects are in favor of ameliorating the oxidative stress and had moderate levels of evidence in GRADE framework (Supplementary Table 2, Supplemental Digital Content 1, http://links.lww.com/JS9/D229).
- Cardiac biomarkers

The meta-analysis demonstrated that sevoflurane preconditioning significantly decreased the serum level of Troponin-I (SMD=−1.65, 95% CI: −2.53 to −0.78, *P*<0.0001; *I*²=67.26%, *P*=0.044) and lactate LDH (SMD=−4.85, 95% CI: −7.42 to −2.28, *P*<0.0001; *I*²=89.68%, *P*<0.0001), compared to nontreated animals (Table 2, Supplementary Figure 7B, Supplemental Digital Content 1, http://links.lww.com/JS9/D229). Both of these effects had moderate levels of evidence in GRADE framework (Supplementary Table 2, Supplemental Digital Content 1, http://links.lww.com/JS9/D229).

**Table 4:** Subgroup analyses and meta-regressions for different variables in sevoflurane preconditioning.

		Subgroup analysis			Meta-regression	
	Number of experiments	SMD [95% CI]	*P*	*I* ^2^ (%)	Coefficient [95% CI]	*P*
Infarct size						
Animal						
Mice	5	−2.63 [−4.49, −0.78]	0.005	92.89	Reference	
Rat	11	−3.56 [−4.19, −2.93]	<0.0001	36.35	−1.29 [−2.68, 0.10]	0.07
I/R model						
Langendorf	1	Insufficient data			N/A	
LAD/LMCA ligation	16	−3.30 [−4.08, −2.53]	<0.0001	78.80		
Ischemia duration						
25 min	2	Insufficient data			Insufficient data	
30 min	12	−3.47 [−4.37, −2.56]	<0.0001	79.61	Reference	
45 min	3	−3.49 [−5.92, −1.05]	0.005	82.40	0.10 [−2.09, 2.28]	0.929
Injury to treatment interval						
≤30 min	9	−3.21 [−4.01, −2.41]	<0.0001	62.60	Reference	
>30 min	8	−3.70 [−5.14, −2.27]	<0.0001	87.28	−0.31 [−1.89, 1.27]	0.701
Frequency of administration						
Single-dose	9	−3.67 [−4.57, −2.78]	<0.0001	61.87	Reference	
Multidose	8	−3.16 [−4.46, −1.86]	<0.0001	87.85	0.65 [−0.88, 2.18]	0.405
Treatment duration						
≤10 min	3	−3.90 [−6.00, −1.80]	<0.0001	78.96	Reference	
>10 min	14	−3.33 [−4.17, −2.49]	<0.0001	79.59	0.52 [−1.54, 2.59]	0.619
Sevoflurane dose						
≤2.4%	14	−3.48 [−4.42, −2.54]	<0.0001	83.48	Reference	
>2.4%	3	−3.33 [−4.18, −2.47]	<0.0001	0.00	0.11 [−1.94, 2.15]	0.919
TUNEL-positive cells						
Animal						
Mice	2	Insufficient data			N/A	
Rat	8	−3.21 [−4.40, −2.03]	<0.0001	75.08		
I/R model						
Langendorf	0	Insufficient data				
LAD/LMCA ligation	11	−3.47 [−4.59, −2.34]	<0.0001	82.09	N/A	
Ischemia duration						
30 min	8	−3.45 [−4.85, −2.05]	<0.0001	85.03	Reference	
45 min	3	−3.58 [−5.76, −1.40]	0.001	76.40	−0.16 [−2.85, 2.52]	0.906
Injury to treatment interval						
≤30 min	4	−2.57 [−4.08, −1.06]	0.001	74.45	Reference	
>30 min	7	−4.01 [−5.51, −2.51]	<0.0001	82.60	−1.38 [−3.68, 0.92]	0.239
Frequency of administration						
Single-dose	4	−3.99 [−5.80, −2.17]	<0.0001	73.69	Reference	
Multidose	7	−3.20 [−4.86, −1.72]	<0.0001	85.73	0.82 [−1.60, 3.24]	0.507
Treatment duration						
≤10 min	0	Insufficient data			N/A	
>10 min	11	−3.47 [−4.59, −2.34]	<0.0001	82.09		
Sevoflurane dose						
≤2.4%	9	−3.07 [−4.25, −1.90]	<0.0001	82.17	N/A	
>2.4%	2	Insufficient data				

#### Effect of sevoflurane postconditioning on myocardial I/R injury

Post-treatment administration of sevoflurane was defined as the administration of sevoflurane after the induction of I/R injury. Thirty-four studies including 319 analyses were included in the meta-analysis. The findings of this section are presented in Table 3. No evidence of publication bias was observed in this part of the study (Supplementary Figure 8, Supplemental Digital Content 1, http://links.lww.com/JS9/D229).

- Cardiac function
  Pooled data analysis demonstrated that postconditioning of sevoflurane significantly increases negative dp/dt (SMD=3.13, 95% CI: 2.51–3.75, *P*<0.0001; *I*²=79.44%) and positive dp/dt (SMD=2.03, 95% CI: 1.33–2.72, *P*<0.0001; *I*²=90.54%) (Table 5, Supplementary Figure 9, Supplemental Digital Content 1, http://links.lww.com/JS9/D229). The meta-analysis also exhibited meaningful enhancement in LVDP (SMD=1.56, 95% CI: 1.00–2.13, *P*<0.0001; *I*²=77.98%) and LVSP (SMD=2.81, 95% CI: 1.79–2.83, *P*<0.0001; *I*²=82.61%) in sevoflurane postconditioning group compared to those in I/R injury group (Table 5, Supplementary Figure 10, Supplemental Digital Content 1, http://links.lww.com/JS9/D229). The results were similar on LVEF (SMD=4.40, 95% CI: 3.06–5.74, *P*<0.0001; *I*²=79.41%), LVFS (SMD=3.49, 95% CI: 1.90–5.09, *P*<0.0001; *I*²=87.69%), and HR (SMD=1.07, 95% CI: 0.51–1.63, *P*<0.0001; *I*²=89.17%) (Table 5, Supplementary Figure 11-12, Supplemental Digital Content 1, http://links.lww.com/JS9/D229). On the other hand, sevoflurane postconditioning decreased LVEDP (SMD=−2.06, 95% CI: −3.16 to −0.97, *P*<0.0001; *I*²=95.64; Supplementary Figure 10, Supplemental Digital Content 1, http://links.lww.com/JS9/D229), LVIDd (SMD=−2.22, 95% CI: −3.13 to −1.31, *P*<0.0001; *I*²=60.65%; Supplementary Figure 11, Supplemental Digital Content 1, http://links.lww.com/JS9/D229), and LVIDs (SMD=−2.48, 95% CI: −3.09 to −1.88, *P*<0.0001; *I*²=5.40%; Supplementary Figure 11, Supplemental Digital Content 1, http://links.lww.com/JS9/D229).
  The enhancement of negative and positive dp/dt and LVDP was evident in all subgroups (Table 6). Meta-regressions demonstrated that the enhancement of negative dp/dt was significantly lower in rats compared to mice, which was applied only in three distinct experiments (meta-regression coefficient=−1.86, 95% CI: −3.47 to −0.24; *P*=0.024) (Table 6). Additionally, the analyses demonstrated that sevoflurane postconditioning decreased LVEDP in all subgroups (Table 6). However, meta-regression suggested that the end-diastolic pressure was significantly more suppressed in mice than in rats (meta-regression coefficient=−3.05, 95% CI: −5.82 to −0.28; *P*=0.031) (Table 6). Finally, there is a considerable diversity in the increasing effect of sevoflurane postconditioning on HR. As presented in Table 6, subgroup analysis showed that the enhancement of HR due to sevoflurane postconditioning is not statistically significant in mice (SMD=0.26, 95% CI: −0.97 to 1.49, *P*=0.679; *I* ^2^=77.01%), and LAD/LMCA ligated animals (SMD=0.87, 95% CI: −0.13 to 1.87, *P*=0.090; *I* ^2^=93.19%). Moreover, the effect size was not meaningful in animals that went under ischemia for more than 30 min (SMD=0.29, 95% CI: −0.51 to 1.09, *P*=0.483; *I* ^2^=69.13%), and treatment duration equal to or less than 10 min (SMD=0.38, 95% CI: −0.54 to 1.30, *P*=0.413; *I* ^2^=90.18%) (Table 6). Subsequently, meta-regression showed a significant difference of effect size in treatment duration of ≤10 min and >10 min (meta-regression coefficient=1.08, 95% CI: 0.01–2.16; *P*=0.049) (Table 6). Meta-regression also demonstrated that the heart rate escalation was not dose-dependent (meta-regression coefficient=−0.38, 95% CI: −1.56 to 0.80; *P*=0.528) (Table 6). The GRADE of evidence for the therapeutic effects of sevoflurane postconditioning on cardiac function following myocardial I/R injury are presented in Supplementary Table 3 (Supplemental Digital Content 1, http://links.lww.com/JS9/D229).
- Myocardial infarct size
  Moderate level of evidence from pooled data analysis showed a significant reduction of infarct size following sevoflurane postconditioning (SMD=−3.42, 95% CI: −3.93 to −2.90, *P*<0.0001; *I*²=81.93%) (Table 5, Supplementary Figure 13, Supplemental Digital Content 1, http://links.lww.com/JS9/D229).
  The improvement in infarct size was evident in all subgroups. Meta-regressions demonstrated that the extent of improvement was not statistically different in any of the subgroups (Table 6).
- Apoptosis
  Pooled data analysis revealed that sevoflurane postconditioning has been implicated in cardioprotection by preventing apoptosis. This is evident by significantly less TUNEL-positive myocytes (SMD=−2.83, 95% CI: −3.54 to −2.12, *P*<0.0001; *I*²=81.31%), higher Bcl-2 levels (SMD=5.40, 95% CI: 3.09 to 7.72, *P*<0.0001; *I*²=86.82%), and lower Caspase-3 levels (SMD=-4.09, 95% CI: −5.59 to −2.60, *P*<0.0001; *I*²=76.59%) in sevoflurane postconditioning group compared to the no-treatment I/R group (Table 5, Supplementary Figure 14, Supplemental Digital Content 1, http://links.lww.com/JS9/D229).
  The reduction of TUNEL-positive cells in the sevoflurane postconditioning group was evident in all subgroups. Meta-regressions demonstrated that the extent of improvement was significantly lower in rats than in mice (meta-regression coefficient=1.63, 95% CI: 0.03–3.24; *P*=0.046) (Table 6). The level of evidence was assessed as high for the effect of sevoflurane postconditioning on TUNEL-positive cells, and moderate for Bcl-2 and Caspase-3 (Supplementary Table 3, Supplemental Digital Content 1, http://links.lww.com/JS9/D229).
- Inflammation
  Moderate level of evidence from pooled data analysis showed that inflammation in rodents with myocardial I/R injury is repressed by sevoflurane postconditioning. This is evident by the suppression of IL-1β (SMD=−5.39, 95% CI: −6.62 to −4.15, *P*<0.0001; *I*²=57.59%), IL-6 (SMD=−8.88, 95% CI: −14.35 to −3.41, *P*=0.001; *I*²=97.35%), and TNF-α (SMD=-4.71, 95% CI: −6.60 to −2.83, *P*<0.0001; *I*²=87.73%) following myocardial I/R injury (Table 5, Supplementary Figure 15, Supplemental Digital Content 1, http://links.lww.com/JS9/D229).
- Oxidative stress
  Pooled data analysis demonstrated that oxidative stress in rodents with myocardial I/R injury is suppressed by sevoflurane postconditioning. This is evident by increased expression of GSH (SMD=6.23, 95% CI: 1.17–11.29, *P*=0.016; *I*²=97.05%) and SOD (SMD=4.72, 95% CI: 3.22–6.21, *P*<0.0001; *I*²=86.43%) and suppression of MDA (SMD=−4.03, 95% CI: −5.09 to −2.98, *P*<0.0001; *I*²=71.57%) (Table 5, Supplementary Figure 16, Supplemental Digital Content 1, http://links.lww.com/JS9/D229).
  Subgroup analyses demonstrated that sevoflurane postconditioning could suppress MDA in all subgroups. Subsequently, meta-regression showed an advantage of sevoflurane postconditioning in suppressing MDA levels when animals undergo treatment for more than 10 min (meta-regression coefficient=−1.93, 95% CI: −3.76 to −0.11; *P*=0.037). The level of evidence was moderate for GSH and SOD, and high for MDA (Table 6).
- Cardiac biomarkers

Moderate level of evidence from pooled data analysis on major biomarkers of myocardial cellular injury revealed that sevoflurane postconditioning significantly decreased the release of CK-MB (SMD=−3.11, 95% CI: −4.99 to −1.23, *P*=0.001; *I*²=94.30%), cardiac Troponin-I (SMD=−1.93, 95% CI: −2.51 to −1.35, *P*<0.0001; *I*²=71.22%), and LDH (SMD=−4.46, 95% CI: −5.95 to −2.96, *P*<0.0001; *I*²=86.56%) into plasma after myocardial I/R injury (Table 5, Supplementary Figure 17, Supplemental Digital Content 1, http://links.lww.com/JS9/D229).

In the next step, subgroup analyses showed that the therapeutic effect is present among all subgroups. Meta-regression showed no significant differences among experiments that treated animals within or more than 30 min postinjury (meta-regression coefficient=0.10, 95% CI: −0.17 to 1.37; *P*=0.877) (Table 6).

In order to indirectly compare the therapeutic effect of sevoflurane among pretreatment, preconditioning, and postconditioning administration of sevoflurane on infarct size, we performed a meta-regression. The analysis showed that although all treatment conditions could diminish the infarct size, the effect size for pretreatment is significantly smaller than preconditioning (meta-regression coefficient=−1.37, 95% CI: −2.50 to −0.23; *P*=0.019) and postconditioning administration of sevoflurane (meta-regression coefficient=−1.30, 95% CI: −2.25 to −0.35; *P*=0.007). Interestingly, there is no difference between preconditioning and postconditioning treatment in terms of effect size (meta-regression coefficient=0.05, 95% CI: −0.92 to 1.02; *P*=0.917).

**Table 5:** The efficacy of sevoflurane postconditioning in myocardial ischemia/reperfusion injury.

Outcome	Number of experiments	*P* for publication bias	*I* ^2^ (*P*)	SMD (95% CI)	*P*	Level of evidence^a^
Cardiac function						
-dp/dt	20	0.161	79.44% (<0.0001)	3.13 (2.51–3.75)	<0.0001	High
+dp/dt	24	0.848	90.54% (<0.0001)	2.03 (1.33–2.72)	<0.0001	High
LVDP	13	0.055	77.98% (<0.0001)	1.56 (1.00–2.13)	<0.0001	High
LVEDP	19	0.782	95.64% (<0.0001)	−2.06 (−3.16, −0.97)	<0.0001	Moderate
LVSP	8	NA	82.61% (0.0001)	2.81 (1.79–3.83)	<0.0001	Moderate
LVIDd	4	NA	60.65% (0.064)	−2.22 (−3.13, −1.31)	<0.0001	Moderate
LVIDs	4	NA	5.40% (0.445)	−2.48 (−3.09, −1.88)	<0.0001	High
LVEF	7	NA	79.41% (0.0001)	4.40 (3.06–5.74)	<0.0001	Moderate
LVFS	6	NA	87.69% (<0.0001)	3.49 (1.90–5.09)	<0.0001	Moderate
HR	28	0.082	89.17% (<0.0001)	1.07 (0.51–1.63)	<0.0001	High
Infarct size	50	0.407	81.93% (<0.0001)	−3.42 (−3.93, −2.90)	<0.0001	Moderate
Apoptosis						
TUNEL-positive cells	23	0.116	81.31% (<0.0001)	−2.83 (−3.54, −2.12)	<0.0001	High
Bcl-2	5	NA	86.82% (0.004)	5.40 (3.09–7.72)	<0.0001	Moderate
Caspase-3	7	NA	76.59% (0.0002)	−4.09 (−5.59, −2.60)	<0.0001	Moderate
Inflammation						
IL-1β	5	NA	57.59% (0.034)	−5.39 (−6.62, −4.15)	<0.0001	Moderate
IL-6	6	NA	97.35% (<0.0001)	−8.88 (−14.35, −3.41)	0.001	Moderate
TNF-α	6	NA	87.73% (<0.0001)	−4.71 (−6.60, −2.83)	<0.0001	Moderate
Oxidative stress						
GSH	5	NA	97.05% (<0.0001)	6.23 (1.17–11.29)	0.016	Moderate
MDA	10	0.212	71.57% (0.0002)	−4.03 (−5.09, −2.98)	<0.0001	High
SOD	9	NA	86.43% (<0.0001)	4.72 (3.22–6.21)	<0.0001	Moderate
Cardiac biomarkers						
CK-MB	8	NA	94.30% (<0.0001)	−3.11 (−4.99, −1.23)	0.001	Moderate
Troponin-I	11	0.426	71.22% (0.0002)	−1.93 (−2.51, −1.35)	<0.0001	Moderate
LDH	10	0.860	86.56% (<0.0001)	−4.46 (−5.95, −2.96)	<0.0001	Moderate

^a^ Based on the GRADE framework. The details are presented in supplementary Table 2 (Supplemental Digital Content 1, http://links.lww.com/JS9/D229).

+dp/dt, Maximal rate of left ventricular pressure; Bcl-2, B-cell lymphoma 2; CK-MB, Creatine kinase-myocardial band; -dp/dt, Maximal decline in left ventricular pressure; GSH, glutathione; HR, heart rate; IL-1β, interleukin-1 beta; IL-6, interleukin-6; LDH, lactate dehydrogenase; LVDP, left ventricular diastolic pressure; LVEDP, left ventricular end-diastolic pressure; LVEF, left ventricular ejection fraction; LVFS, left ventricular fractional shortening; LVIDd, left ventricular internal diameter at end-diastole; LVIDs, left ventricular internal diameter at end-systole; LVSP, left ventricular systolic pressure; MDA, malondialdehyde; NA, not applicable due to the insufficient number of experiments; SMD, standardize mean difference; SOD, superoxide dismutase; TNF-α, tumor necrosis factor alpha.

**Table 6:** Subgroup analyses and meta-regressions for different variables in sevoflurane postconditioning.

		Subgroup analysis			Meta-regression	
	Number of experiments	SMD [95% CI]	*P*	*I* ^2^ (%)	Coefficient [95% CI]	*P*
-dp/dt						
Animal						
Mice	3	4.85 [2.68–7.02]	<0.0001	87.49	Reference	
Rat	17	2.82 [2.24–3.41]	<0.0001	72.28	−1.86 [−3.47, −0.24]	0.024
I/R model						
Langendorf	13	2.70 [2.03–3.37]	<0.0001	75.14	Reference	
LAD/LMCA ligation	7	3.96 [2.82–5.11]	<0.0001	78.63	1.22 [−0.02, 2.46]	0.054
Ischemia duration						
≤30 min	20	3.13 [2.51–3.75]	<0.0001	79.44	N/A	
>30 min	0	Insufficient data				
Injury to treatment interval						
≤30 min	13	3.01 [2.15–3.88]	<0.0001	84.75	Reference	
31–45 min	5	3.92 [3.26, 4.58]	<0.0001	0.00	0.99 [−0.48, 2.45]	0.187
46–60 min	2	Insufficient data			Insufficient data	
Treatment duration						
≤10 min	7	3.71 [2.45– 4.96]	<0.0001	84.07	Reference	
>10 min	13	2.84 [2.14–3.54]	<0.0001	75.82	−0.80 [−2.10, 0.50]	0.229
Sevoflurane dose						
≤2.4%	7	3.01 [1.70– 4.32]	<0.0001	85.24	Reference	
**>**2.4%	13	3.22 [2.50– 3.93]	<0.0001	77.31	0.28 [−1.06, 1.62]	0.680
+dp/dt						
Animal						
Mice	2	Insufficient data			N/A	
Rat	22	1.89 [1.17–2.61]	<0.0001	90.39		
I/R model						
Langendorf	18	1.65 [0.91– 2.39]	<0.0001	89.80	Reference	
LAD/LMCA ligation	6	3.22 [1.72–4.72]	<0.0001	88.77	1.53 [−0.02, 3.08]	0.054
Ischemia duration						
25 min	4	−0.60 [−1.00, −1.19]	0.004	0.00	Reference	
30 min	19	2.64 [2.11–3.18]	<0.0001	74.24	3.22 [2.16–4.29]	<0.0001
40 min	1	Insufficient data			Insufficient data	
Injury to treatment interval						
≤30 min	16	1.85 [0.87–2.84]	<0.0001	93.04	Reference	
31 45 min	6	2.39 [1.42–3.37]	<0.0001	80.15	0.62 [−1.09, 2.33]	0.477
46–60 min	2	Insufficient data			Insufficient data	
Treatment duration						
≤10 min	6	2.89 [1.49–4.29]	<0.0001	88.42	Reference	
>10 min	18	1.75 [0.97– 2.53]	<0.0001	90.66	−1.13 [−2.73, 0.46]	0.163
Sevoflurane dose						
≤2.4%	9	1.35 [0.36–2.34]	<0.0001	89.04	Reference	
**>**2.4%	15	2.43 [1.53–3.34]	<0.0001	90.14	1.06 [−0.33, 2.44]	0.136
LVDP						
Animal						
Mice	0	Insufficient data			N/A	
Rat	13	1.56 [1.00–2.13]	<0.0001	77.98		
I/R model						
Langendorf	12	1.51 [0.92–2.11]	<0.0001	79.37	N/A	
LAD/LMCA ligation	1	Insufficient data				
Ischemia duration						
≤30 min	12	1.68 [1.11–2.25]	<0.0001	73.80	N/A	
>30 min	1	Insufficient data				
Injury to treatment interval						
≤30 min	5	1.89 [0.92–2.86]	<0.0001	76.93	Reference	
31–45 min	6	1.70 [0.87–2.53]	<0.0001	78.26	−0.17 [−1.44, 1.09]	0.788
46–60 min	2	Insufficient data			Insufficient data	
Treatment duration						
≤10 min	2	Insufficient data			N/A	
>10 min	11	1.53 [0.87–2.19]	<0.0001	81.77		
Sevoflurane dose						
≤2.4%	3	1.27 [0.16–2.39]	0.025	78.38	Reference	
**>**2.4%	10	1.66 [0.98–2.35]	<0.0001	78.79	0.37 [−1.00, 1.75]	0.594
LVEDP						
Animal						
Rat	16	−1.55 [−2.54, −0.55]	0.002	93.81	Reference	
Mice	3	−4.73 [−8.34, −1.13]	0.01	95.64	−3.05 [−5.82, −0.28]	0.031
I/R model						
Langendorf	10	−1.26 [−2.51, −0.01]	0.048	94.61	Reference	
LAD/LMCA ligation	9	−2.95 [−4.70, −1.19]	0.001	95.18	−1.61 [−3.74, 0.51]	0.136
Ischemia duration						
≤30 min	18	−2.15 [−3.31, −0.99]	<0.0001	95.47	N/A	
>30 min	1	Insufficient data				
Injury to treatment interval						
≤30 min	18	−2.15 [−3.31, −0.99]	<0.0001	95.47	N/A	
31–45 min	1	Insufficient data				
46–60 min	0	Insufficient data				
Treatment duration						
≤10 min	9	−2.33 [−3.89, −0.77]	0.003	94.84	Reference	
>10 min	10	−1.83 [−3.44, −0.23]	0.025	96.39	0.52 [−1.73, 2.77]	0.654
Sevoflurane dose						
≤2.4%	12	−2.17 [−3.68, −0.67]	0.005	96.04	Reference	
**>**2.4%	7	−1.92 [−3.61, −0.23]	0.026	95.41	0.23 [−2.11, 2.57]	0.848
HR						
Animal						
Mice	3	0.26 [−0.97, 1.49]	0.679	77.01	Reference	
Rat	25	1.19 [0.57–1.81]	<0.0001	90.30	0.90 [−0.90, 2.71]	0.327
I/R model						
Langendorf	13	1.38 [0.82–1.93]	<0.0001	78.10	Reference	
LAD/LMCA ligation	15	0.87 [−0.13, 1.87]	0.09	93.19	−0.74 [−1.79, 0.32]	0.170
Ischemia duration						
≤30 min	24	1.24 [0.58–1.90]	<0.0001	90.39	Reference	
>30 min	4	0.29 [−0.51, 1.09]	0.483	69.13	−0.91 [−2.48, 0.67]	0.259
Injury to treatment interval						
≤30 min	17	1.50 [0.46–2.54]	0.005	94.12	Reference	
31–45 min	9	0.77 [0.25–1.29]	0.004	66.92	−0.57 [−1.90, 0.75]	0.397
46–60 min	2	Insufficient data			Insufficient data	
Treatment duration						
≤10 min	10	0.38 [−0.54, 1.30]	0.413	90.18	Reference	
>10 min	18	1.43 [0.81–2.04]	<0.0001	84.90	1.08 [0.01–2.16]	0.049
Sevoflurane dose						
≤2.4%	17	1.39 [0.38–2.39]	0.007	93.81	Reference	
**>**2.4%	11	0.85 [0.26–1.44]	0.005	79.45	−0.38 [−1.56, 0.80]	0.528
Infarct size						
Animal						
Mice	8	−3.89 [−4.85, −2.92]	<0.0001	63.32	Reference	
Rat	42	−3.35 [−3.94, −2.76]	<0.0001	83.88	0.64 [−0.75, 2.03]	0.368
I/R model						
Langendorf	20	−2.77 [−3.37, −2.17]	<0.0001	72.30	Reference	
LAD/LMCA ligation	30	−3.91 [−4.68, −3.14]	<0.0001	84.22	−0.95 [−1.98, 0.08]	0.071
Ischemia duration						
≤30 min	46	−3.47 [−4.03, −2.91]	<0.0001	83.24	Reference	
>30 min	4	−3.09 [−4.33, −1.85]	<0.0001	62.82	0.28 [−1.59, 2.15]	0.769
Injury to treatment interval						
≤30 min	39	−3.71 [−4.36, −3.07]	<0.0001	85.16	Reference	
31–45 min	9	−2.88 [−3.54, −2.21]	<0.0001	36.51	0.65 [−0.69, 1.98]	0.342
46–60 min	2	Insufficient data				
Treatment duration						
≤10 min	23	−3.60 [−4.35, −2.85]	<0.0001	80.66	Reference	
>10 min	27	−3.27 [−3.99, −2.56]	<0.0001	83.12	0.34 [−0.69, 1.38]	0.516
Sevoflurane dose						
≤2.4%	27	−3.61 [−4.35, −2.87]	<0.0001	84.08	Reference	
**>**2.4%	23	−3.21 [−3.93, −2.48]	<0.0001	79.43	0.33 [−0.71, 1.38]	0.553
TUNEL-positive cells						
Animal						
Mice	5	−4.05 [−4.76, −3.35]	<0.0001	3.44	Reference	
Rat	18	−2.49 [−3.27, −1.70]	<0.0001	81.22	1.63 [0.03–3.24]	0.046
I/R model						
Langendorf	6	−2.30 [−3.25, −1.29]	<0.0001	63.07	Reference	
LAD/LMCA ligation	17	−3.04 [−3.94, −2.14]	<0.0001	84.15	−0.67 [−2.29, 0.96]	0.421
Ischemia duration						
≤0 min	21	−2.64 [−3.35, −1.93]	<0.0001	80.65	N/A	
>30 min	2	Insufficient data				
Injury to treatment interval						
≤30 min	17	−2.78 [−3.60, −1.97]	<0.0001	82.17	Reference	
31–45 min	4	−4.11 [−5.22, −3.01]	<0.0001	19.69	−1.43 [−3.33, 0.47]	0.139
46–60 min	2	Insufficient data				
Treatment duration						
≤10 min	14	−2.83 [−3.82, −1.84]	<0.0001	85.75	Reference	
>10 min	9	−2.84 [−3.84, −1.85]	<0.0001	71.13	−0.09 [−1.58, 1.40]	0.908
Sevoflurane dose						
≤2.4%	10	−2.78 [−3.96, −1.60]	<0.0001	85.70	Reference	
**>**2.4%	13	−2.88 [−3.79, −1.98]	<0.0001	77.72	−0.28 [−0.88, 0.33]	0.372
MDA						
Animal						
Mice	2	Insufficient data			N/A	
Rat	8	−3.55 [−4.54, −2.55]	<0.0001	65.68		
I/R model						
Langendorf	0	Insufficient data			N/A	
LAD/LMCA ligation	10	−4.03 [−5.09, −2.98]	<0.0001	71.57		
**Ischemia duration**						
≤30 min	8	−3.55 [−4.54, −2.55]	<0.0001	65.68	N/A	
>30 min	2	Insufficient data				
Injury to treatment interval						
≤30 min	8	−3.55 [−4.54, −2.55]	<0.0001	65.68	N/A	
31–45 min	2	Insufficient data				
46–60 min	0	Insufficient data				
Treatment duration						
≤10 min	5	−3.08 [−4.22, −1.95]	<0.0001	62.15	Reference	
>10 min	5	−5.07 [−6.52, −3.61]	<0.0001	58.89	−1.93 [−3.76, −0.11]	0.037
Sevoflurane dose						
≤2.4%	6	−4.51 [−6.23, −2.78]	<0.0001	80.33	Reference	
**>**2.4%	3	−3.49 [−4.53, −2.46]	<0.0001	10.78	0.81 [−1.80, 3.43]	0.542
Troponin-I						
Animal						
Mice	0	Insufficient data			N/A	
Rat	11	−1.93 [−2.51, −1.35]	<0.0001	71.22		
I/R model						
Langendorf	9	−2.01 [−2.66, −1.36]	<0.0001	69.80	N/A	
LAD/LMCA ligation	2	Insufficient data				
Ischemia duration						
≤30 min	11	−1.93 [−2.51, −1.35]	<0.0001	71.22	N/A	
>30 min	0	Insufficient data				
Injury to treatment interval						
≤30 min	4	−2.32 [−3.53, −1.11]	<0.0001	79.59	Reference	
31–45 min	5	−2.13 [−2.73, −1.53]	<0.0001	34.99	0.10 [−0.17, 1.37]	0.877
46–60 min	2	Insufficient data			Insufficient data	
Treatment duration						
≤10 min	1	Insufficient data			N/A	
>10 min	10	−1.87 [−2.50, −1.25]	<0.0001	72.74		
Sevoflurane dose						
≤2.4%	2	Insufficient data			N/A	
**>**2.4%	9	−2.01 [−2.66, −1.36]	<0.0001	69.80		
LDH						
Animal						
Mice	1	Insufficient data			N/A	
Rat	9	−4.34 [−8.20, −3.46]	<0.0001	88.15		
I/R model						
Langendorf	2	Insufficient data			N/A	
LAD/LMCA ligation	8	−4.38 [−5.60, −3.17]	<0.0001	71.54		
Ischemia duration						
≤30 min	9	−4.34 [−5.97, −2.71]	<0.0001	88.15	N/A	
>30 min	1	Insufficient data				
Injury to treatment interval						
≤30 min	9	−4.34 [−5.97, −2.71]	<0.0001	88.15	N/A	
31–45 min	1	Insufficient data				
46–60 min	0	Insufficient data				
Treatment duration						
≤10 min	4	−3.35 [−4.70, −2.00]	<0.0001	61.42	Reference	
>10 min	6	−5.43 [−7.93, −2.92]	<0.0001	90.89	−1.78 [−4.88, 1.31]	0.258
Sevoflurane dose						
≤2.4%	6	−5.53 [−7.82, −3.24]	<0.0001	87.33	Reference	
**>**2.4%	3	−2.94 [−5.06, −0.81]	0.007	81.89	2.45 [−0.98, 5.87]	0.162

### Risk of bias assessment

The allocation sequence was appropriately generated and implemented in 35 of the studies. None of the studies clearly disclosed allocation concealment, nor was there any mention of random housing during the experiments. Caregiver blinding was not clearly disclosed in any of the studies, whereas outcome assessor blinding was disclosed in 8 studies. It was unclear if a random selection process was used for outcome assessment in any of the studies. There appeared to be no incomplete outcome data or other factors that could potentially cause bias (Table 7).

**Table 7:** Risk of bias assessment of the included studies based on the CYRCLE tool.

Study	Sequence generation	Baseline characteristics	Allocation concealment	Random housing	Blinding trial caregivers	Random outcome assessment	Blinding outcome assessors	Incomplete outcome data	Selective outcome reporting	Other sources of bias
An, 2023^19^	L	L	U	U	U	U	U	L	L	L
Behmenburg, 2017^20^	L	L	U	U	U	U	U	L	L	L
Cao, 2015^21^	L	L	U	U	U	U	U	L	L	L
Deng, 2022^22^	U	L	U	U	U	U	U	L	L	L
Drenger, 2011^23^	L	L	U	U	U	U	L	L	L	L
Frassdorf, 2010^24^	L	L	U	U	U	U	U	L	L	L
Gao, 2016^26^	L	L	U	U	U	U	U	L	L	L
Gao, 2021^25^	L	L	U	U	U	U	U	L	L	L
Geng, 2022	U	L	U	U	U	U	U	L	L	L
Hong, 2020^27^	U	L	U	U	U	U	U	L	L	L
Huang, 2019^28^	U	L	U	U	U	U	U	L	L	L
Huhn, 2008^29^	U	L	U	U	U	U	U	L	L	L
Li, 2013^31^	L	L	U	U	U	U	U	L	L	L
Li, 2015	L	L	U	U	U	U	U	L	L	L
Lin, 2016^32^	L	L	U	U	U	U	U	L	L	L
Liu, 2019^33^	L	L	U	U	U	U	U	L	L	L
Ma, 2013^34^	U	L	U	U	U	U	U	L	L	L
Obal, 2001^36^	U	L	U	U	U	U	U	L	L	L
Obal, 2003^37^	U	L	U	U	U	U	L	L	L	L
Obal, 2005^35^	L	L	U	U	U	U	U	L	L	L
Popescu, 2022^38^	L	L	U	U	U	U	U	L	L	L
Qi, 2019^39^	L	U	U	U	U	U	U	L	L	L
Qiao, 2012	L	L	U	U	U	U	U	L	L	L
Qiao, 2018	U	L	U	U	U	U	U	L	L	L
Qin, 2022^68^	L	L	U	U	U	U	U	L	L	L
Redel, 2006	L	L	U	U	U	L	U	L	L	L
Ru, 2021	U	L	U	U	U	U	U	L	L	L
Song, 2022^43^	U	L	U	U	U	U	U	L	L	L
Stumpner, 2014^44^	U	L	U	U	U	U	L	L	L	L
Tai, 2012^45^	L	L	U	U	U	U	U	L	L	L
Tan, 2020^46^	U	L	U	U	U	U	U	L	L	L
Tosaka, 2011^47^	L	L	U	U	U	U	U	L	L	L
Wang, 2010^48^	L	L	U	U	U	U	U	L	L	L
Wang, 2020	L	L	U	U	U	U	U	L	L	L
Wu, 2021^50^	L	L	U	U	U	U	U	L	L	L
Wu, 2022^51^	U	L	U	U	U	U	U	L	L	L
Xiao, 2011^52^	L	L	U	U	U	U	U	L	L	L
Xie, 2014^54^	L	L	U	U	U	U	L	L	L	L
Xie, 2020^53^	L	L	U	U	U	U	U	L	L	L
Xu, 2013^55^	U	L	U	U	U	U	U	L	L	L
Yang, 2016^56^	L	L	U	U	U	U	U	L	L	L
Yao, 2010^57^	L	L	U	U	U	U	L	L	L	L
Yu, 2014	L	L	U	U	U	U	L	L	L	L
Yu, 2021^58^	L	L	U	U	U	U	U	L	L	L
Zeng, 2022	L	L	U	U	U	U	U	L	L	L
Zhang, 2012^61^	U	L	U	U	U	U	U	L	L	L
Zhang, 2014^62^	L	L	U	U	U	U	L	L	L	L
Zhang, 2020	L	L	U	U	U	L	L	L	L	L
Zhang, 2022^64^	L	L	U	U	U	U	U	L	L	L
Zhao, 2013^65^	L	L	U	U	U	U	U	L	L	L
Zhou, 2017^66^	L	L	U	U	U	U	U	L	L	L

H, high risk of bias; L, low risk of bias; U, unclear risk of bias.

## Discussion

Sevoflurane is widely used in cardiac surgery since it has the advantages of shorter induction, reduced recovery time and higher safety when compared with other anesthetics^21^. Myocardial I/R is an important unfavorable event in the perioperative period of open-heart surgeries and represents a major challenge to perioperative management. As a volatile anesthetic, we presented moderate to high level of evidence of sevoflurane preconditioning cardio-protection and shed light to its mechanisms of action. The results of the current meta-analysis can encourage the wider application of sevoflurane in cardiac surgeries. Our study provides substantial evidence that sevoflurane preconditionings and postconditionings ameliorate myocardial I/R injury and improve cardiac function by inhibiting myocardial tissue apoptosis, oxidative stress, myocardial tissue inflammation, and reducing serum levels of cardiac biomarkers. Our findings indicate that administering sevoflurane at any point between 7 days before ischemia and up to 1 h after ischemia, across a range of standard doses, consistently leads to a significant reduction in myocardial infarct size. In addition, the result of pooled data analysis showed that -dp/dt, +dp/dt, LVEDP, and LVEF were significantly improved by sevoflurane preconditioning. -dp/dt, +dp/dt, LVDP, LVEDP, LVSP, LVIDd, LVIDs, LVED, LVFS, and HR were also improved by sevoflurane postconditioning, suggesting enhanced ventricular, systolic, and diastolic functions.

The results of the current meta-analysis align with previous studies suggesting that sevoflurane postconditioning increases Bcl-2 and decreases Bax expression, improving cardiac function and mitochondrial ultrastructure^70^. Sevoflurane treatment during early reperfusion led to a marked reduction in myocardial infarct size and cleaved caspase-3 expression, and increased Bcl-2 protein expression. Another study confirmed that sevoflurane dramatically improved I/R-induced cardiac insufficiency, suppressed cardiac infarction, and reduced the infarct area^71^.

It is noteworthy that several findings regarding the cardioprotective effects of sevoflurane preconditioning and postconditioning warrant special consideration. Qiao *et al*.^67^ reported that the cardioprotection afforded by sevoflurane postconditioning is age-dependent, with significant protection observed in young animals but not in older ones. Evidence suggests that this age-related cardioprotection of sevoflurane postconditioning may be linked to the inability to activate Akt and Erk1/2 in older animals^31^. Additionally, Obal *et al*.^35^ discovered that a combination of sevoflurane preconditioning and postconditioning can yield additive protection against myocardial I/R injury, partly mediated by the opening of mitochondrial adenosine triphosphate-dependent potassium (mKATP) channels. Furthermore, various cardiovascular disease risk factors, including obesity, hyperlipidemia, and diabetes, can significantly diminish or even eliminate the cardioprotective effects of sevoflurane conditioning^34,58^. As the primary aim of this analysis was to assess the cardioprotective effects of sevoflurane conditioning, preclinical studies conducted on animals with these cardiovascular disease risk factors were specifically excluded. However, it is advisable to take into account these risk factors when determining the optimal protocols for sevoflurane conditioning.

While several in-vivo experiments have demonstrated significant cardioprotective effects of sevoflurane conditioning, clinical trials in humans have produced varied results regarding the efficacy of these treatments. Some studies indicate potential cardioprotective benefits of sevoflurane in patients undergoing coronary artery bypass graft (CABG) surgery, particularly when administered to maintain anesthesia at 1 MAC. These studies have shown improved clinical outcomes and reduced myocardial injury biomarkers following surgery^72,73^. A recent meta-analysis suggests that halogenated agents, including desflurane, isoflurane, and sevoflurane, may decrease the incidence of myocardial infarction, mortality rates, and the need for mechanical ventilation after cardiac surgery, especially in patients undergoing CABG surgery^74^. However, another meta-analysis of 79 randomized controlled trials involving 6219 patients undergoing noncardiac surgery found inconclusive evidence regarding the cardioprotective benefits of sevoflurane^75^. It is worth noting that this meta-analysis used perioperative myocardial infarction or deaths as the primary endpoint, and no such cases were reported in any of the included studies. Similarly, another systematic review and meta-analysis suggest that the use of volatile anesthetics such as sevoflurane, desflurane, or isoflurane may reduce mortality and perioperative complications in patients undergoing cardiac surgery, but not in those undergoing noncardiac surgery^12^. The reasons for the significant differences in the cardioprotective effects of sevoflurane between patients undergoing cardiac and noncardiac surgeries remain unclear. Therefore, further studies are warranted to determine whether the cardio-protection provided by sevoflurane is indeed dependent on the type of surgery and to identify factors that may hinder the translation of sevoflurane’s cardioprotective benefits into improved clinical outcomes for patients.

The molecular mechanisms of myocardial I/R injury are complex and multifactorial. Among the key factors contributing to myocardial I/R injury, oxidative stress plays a crucial role. It begins at the onset of reperfusion, triggering a series of subsequent pathophysiological processes. Oxidative stress is characterized by a severe imbalance between excessive ROS production and impaired antioxidant defense mechanisms. Consequently, ROS are major contributors to adverse ventricular remodeling by promoting myocardial interstitial fibrosis, cardiomyocyte hypertrophy, and cell death^76^. SOD and GSH inhibit reactive oxygen species (ROS) during myocardial I/R by degrading superoxide and hydroxyl free radicals^60^. Increased reactive oxygen plays a critical role in the occurrence and progression of myocardial I/R injury. Our results demonstrated the importance of inhibiting oxidative stress in the effect of sevoflurane preconditioning and postconditioning on improving cardiac function in myocardial I/R injury. Oxygen free radicals are generated explosively in the first few minutes of reperfusion, causing membrane lipid peroxidation and protein dysfunction^77^. By sevoflurane treatment, L-type calcium channels may be directly inhibited, thereby maintaining myocardial calcium homeostasis. Sevoflurane reduces the accumulation of ROS during reperfusion, and it protects myocardial function during reperfusion^69^. For instance, Zhao *et al*.^65^ established both in vivo mouse models of myocardial I/R and in vitro models using adult mouse cardiomyocytes subjected to simulated I/R. Their findings revealed that sevoflurane preconditioning reduced superoxide generation by 43.6% compared to control treatment. Moreover, this antioxidant effect was largely preserved in mice with dominant-negative AMP-activated protein kinase (AMPK-DN), but completely eliminated in mice lacking caveolin-3 (Cav-3KO). These results suggest that sevoflurane-mediated suppression of superoxide generation relies heavily on caveolin-3 and only partially on the AMPK signaling pathway. Additionally, similar to our results, the study demonstrated that sevoflurane preconditioning significantly reduced caspase-3 activity compared to control treatment, indicating inhibition of cardiomyocyte apoptosis as part of the cardioprotective mechanism of sevoflurane preconditioning.

Excessive oxidative stress leads to the opening of the mitochondrial permeability transition pore (MPTP) and the release of cytochrome C, which in turn triggers the intrinsic apoptosis pathway through the activation of caspase-9 and caspase-3^78^. Following reperfusion injury, both oxidative stress and apoptosis contribute to significant cardiomyocyte loss, resulting in an enlarged infarct area. These pathological mechanisms underpin the rationale for developing therapeutic strategies to combat myocardial I/R injury by inhibiting oxidative stress and apoptosis.

Sevoflurane’s antiapoptotic effects have been linked to the activation of the phosphatidylinositol 3-kinase (PI3K)/Akt pathway^79^, regulation of ROS^80^, and inhibition of MPTP opening^81^. Notably, studies have shown that sevoflurane postconditioning leads to downregulation of NHE1 phosphorylation and transcription levels, potentially preventing MPTP opening and subsequent cardiac injury^21,59^. Furthermore, sevoflurane postconditioning increases NO and NOS levels, contributing to its cardioprotective effects^21^. Moreover, sevoflurane postconditioning inhibited the loss of NAD+ levels, which also prevents MPTP opening^21,59^. These findings suggest a multifaceted mechanism by which sevoflurane exerts its protective effects against myocardial I/R injury, involving modulation of NHE1, NO/NOS signaling, and MPTP regulation.

Our comprehensive analysis reveals that if treatment with sevoflurane is administered more than 24 h before I/R injury, its ability to reduce infarct size significantly diminishes. In the case of preconditioning, where treatment occurs within 24 h prior to the injury, it is unnecessary to restrict the therapeutic window to the initial 30 min, as our findings show no significant difference in effect size during this period. Regarding postconditioning, administering treatment within 1 h after the injury is effective; however, there is no evidence to suggest that shorter intervals between injury and treatment provide any additional benefits.

When myocardial I/R injury occurs, phospholipids in the cell membrane are degraded, and arachidonic acid metabolites such as leukotrienes increase, attracting a large number of white blood cells into the injured tissue^60^. Activated inflammatory cells release inflammatory substances, including a large amount of ROS, various proteolytic enzymes, and cytokines, resulting in cytotoxicity, damage to cell structures, and dysfunction^60^. IL-1β promotes vascular endothelial cells to express ICAM-1 and VCAM-1, and leukocytes to express CD11b/CD18 and other adhesion molecules, thereby enhancing the adhesion of leukocytes and vascular endothelial cells, which facilitates leukocyte inflammatory exudation^82^.

Additionally, IL-1 stimulates endothelial cells and neutrophils to produce NO and oxygen free radicals, causing oxidative stress damage. As an inflammatory cascade amplifier, TNF-α can activate and attract monocytes, macrophages, neutrophils, and vascular endothelial cells. It induces and promotes the secretion and release of inflammatory mediators such as IL-1 and IL-8 by these cells, initiating an inflammatory cascade reaction and amplifying the inflammatory responses^60^.

## Limitations

This meta-analysis includes all studies that met our rigorous inclusion criteria, but several limitations warrant special attention. First, there is no data from large animal models, which share more anatomical and physiological similarities with the human heart than small animals. This limitation may affect the interpretation and generalization of our results. Large animal experiments are needed to further validate the favorable effects of sevoflurane conditioning observed in small animal models. Second, the present only includes studies conducted on normal animals without risk factors for cardiovascular diseases, such as aging, diabetes, obesity, hyperlipidemia, and hypertension. Consequently, our results cannot be generalized to animals with these risk factors. Third, the included studies administered sevoflurane at a maximum dose of 2 MAC (4.8%), and there is no evidence regarding the effects of higher doses. Forth, there was considerable heterogeneity among the included studies, though we identified some potential sources. Fifth, there is a scarcity of data on the effects of sevoflurane pretreatment more than 24 h before injury and also on certain outcomes, such as Bax levels for both preconditioning and postconditioning. To address these issues and confirm the therapeutic effect of sevoflurane conditioning on myocardial I/R injury, more animal studies and human RCTs are required.

## Conclusion

Moderate to high-level evidence demonstrated that sevoflurane conditionings provide a strong and highly reproducible cardioprotective effect in animal models of myocardial I/R injury. This beneficial effect is achieved through mechanisms that reduce infarct size, and exhibit antiapoptotic, anti-inflammatory, and antioxidative properties, which can be confirmed by the suppression of cardiac biomarkers in the serum. Sevoflurane conditionings could be applied before the onset of ischemia, during ischemia, or during reperfusion.

## Ethical approval

The present study does not involve patients.

## Consent

The present study does not involve patients.

## Source of funding

This study was funded and supported by Physiology Research Center, Iran University of Medical Sciences, Tehran, Iran (grant number: 1402-11-32-28298).

## Author contribution

M.Y.: study design and conceptualization; A.N. and M.R.: data gathering; H.Z. and M.Y.: analysis; H.Z., M.Y., and A.A.: interpretation. All authors contributed in drafting, revising, and reading and approving the final manuscript.

## Conflicts of interest disclosure

The authors declare no conflicts interests.

## Research registration unique identifying number (UIN)

The protocol of the present study was prospectively registered on the international prospective register of systematic reviews (PROSPERO; CRD42023454464).

## Guarantor

Mahmoud Yousefifard; Physiology Research Center, Iran University of Medical Sciences, Hemmat Highway, Tehran 14665-354, Iran. Tel.: +98 2186704771; E-mail: yousefifard20@gmail.com Hamed Zarei; Physiology Research Center, Iran University of Medical Sciences, Hemmat Highway, Tehran 14665-354, Iran. Tel.: +98 9192516629; E-mail: hamedzareii@gmail.com.

## Data availability statement

The dataset generated and analyzed during the current study is available from the corresponding author upon reasonable request.

## Provenance and peer review

Not commissioned, externally peer-reviewed.

## Supplementary Material
