# Supplementary material for: Protective effect of sevoflurane on myocardial ischemia-reperfusion injury: a systematic review and meta-analysis
Source: Int J Surg. 2024 Aug 2;110(11):7311–30. doi: 10.1097/JS9.0000000000001975 (PMC11573079; doi:10.1097/JS9.0000000000001975)
Supplement: Supplementary file 1 [file js9-110-7311-s001.pdf]

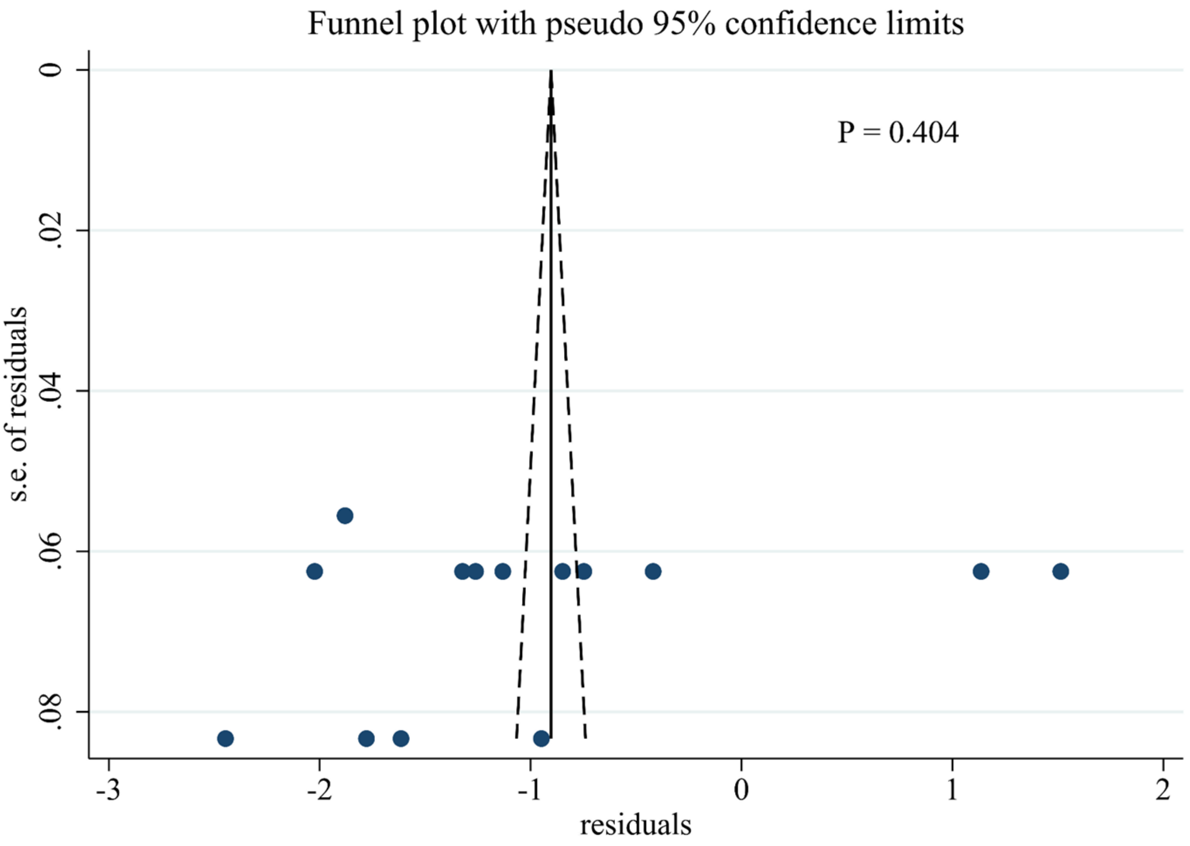

Supplementary Figure 1 Publication bias assessment for the effect of sevoflurane pretreatment on infarct size.

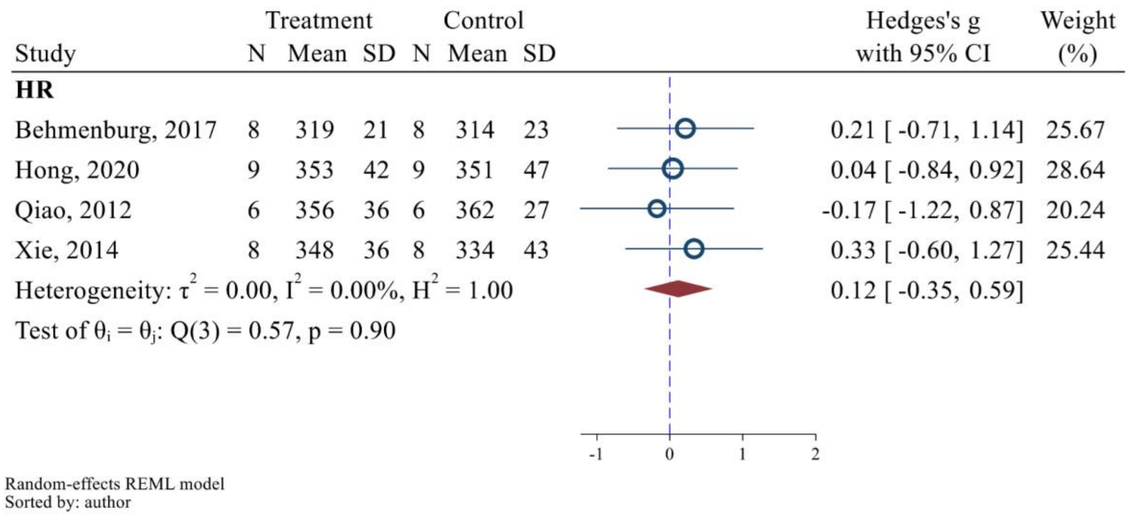

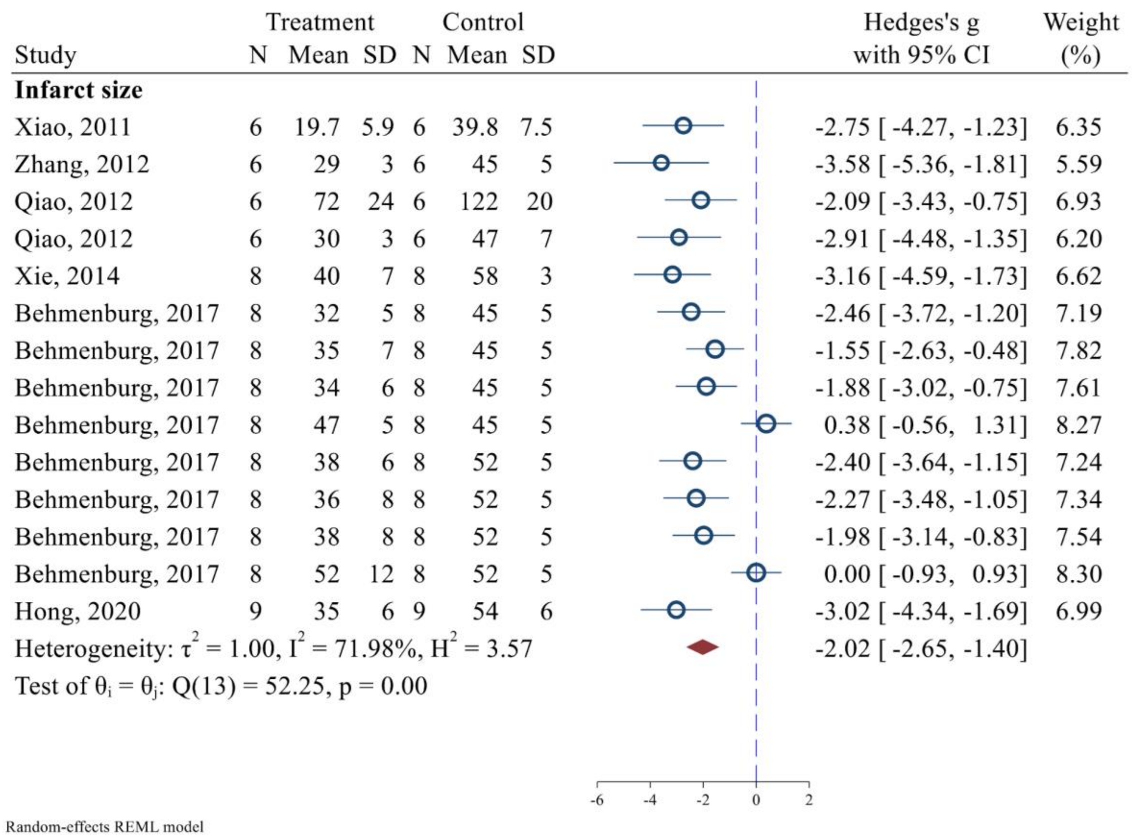

Supplementary Figure 2 The forest plot for the effect of sevoflurane pretreatment on heart rate and infarct size after myocardial I/R injury.

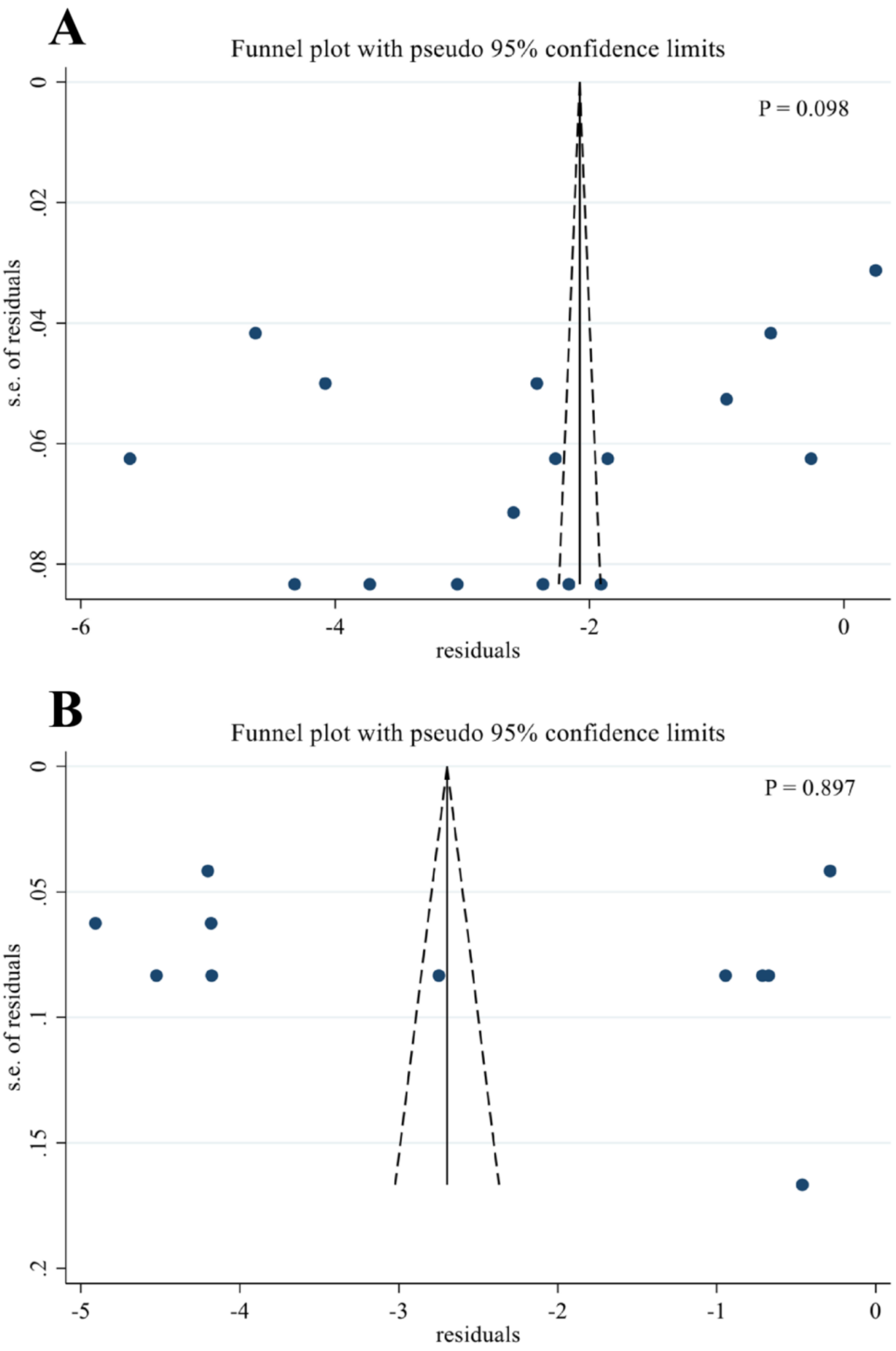

Supplementary Figure 3 Publication bias assessment for the effect of sevoflurane preconditioning on (A) infarct size and (B) TUNEL-positive cells.

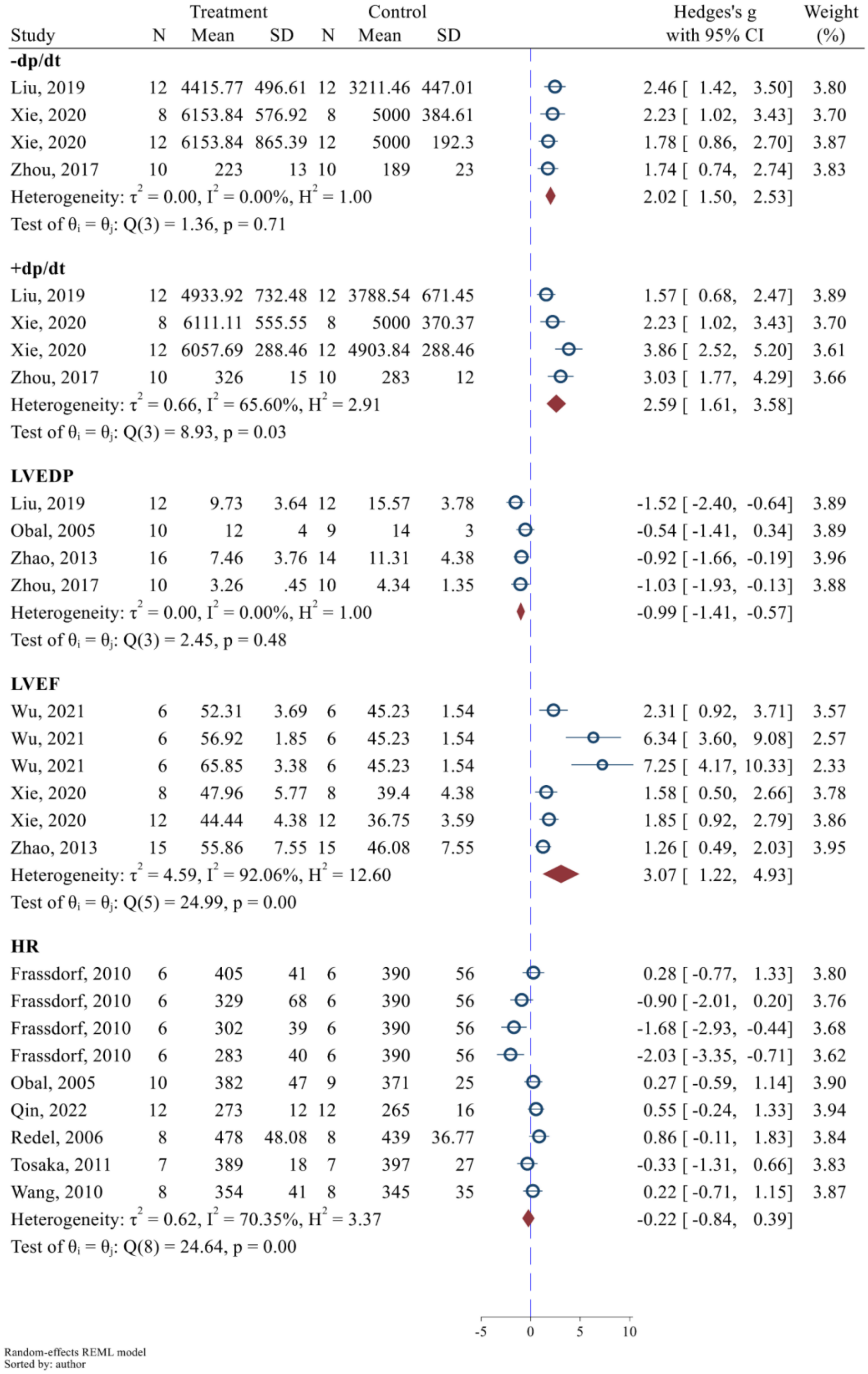

Supplementary Figure 4 The forest plot for the effect of sevoflurane preconditioning on cardiac function after myocardial I/R.

Study	Treatment N	Treatment Mean	Treatment SD	Control N	Control Mean	Control SD	Hedges's g with 95% CI	Weight (%)
**Infarct size**								
Zhang, 2022	12	26.93	3.76	12	34.13	4.35	-1.71 [-2.62, -0.80]	5.67
Qin, 2022	6	16.89	4.52	6	40.39	7.51	-3.50 [-5.25, -1.75]	5.12
Wu, 2021	6	33.23	2.87	6	49.03	4.02	-4.18 [-6.15, -2.21]	4.94
Wu, 2021	6	25.27	4.02	6	49.03	4.02	-5.46 [-7.88, -3.04]	4.56
Wu, 2021	6	17.51	2.11	6	49.03	4.02	-9.06 [-12.84, -5.29]	3.43
Wang, 2020	10	23.31	7.21	10	57.81	11.01	-3.55 [-4.93, -2.17]	5.39
Obal, 2005	10	23.33	12.31	9	48.85	11.28	-2.06 [-3.14, -0.98]	5.58
Redel, 2006	8	26.6	16.97	8	50.4	15.27	-1.39 [-2.44, -0.35]	5.60
Wang, 2010	8	34	6	8	53	6	-2.99 [-4.38, -1.60]	5.38
Frassdorf, 2010	6	42	6	6	67	3	-4.87 [-7.07, -2.66]	4.74
Frassdorf, 2010	6	33	4	6	67	3	-8.88 [-12.58, -5.18]	3.48
Frassdorf, 2010	6	11	5	6	67	3	-12.54 [-17.66, -7.41]	2.53
Frassdorf, 2010	6	16	4	6	67	3	-13.32 [-18.74, -7.89]	2.37
Tosaka, 2011	7	16	7	7	42	6	-3.73 [-5.43, -2.04]	5.16
Ma, 2013	8	26	5	8	44	5	-3.40 [-4.90, -1.90]	5.30
Zhao, 2013	16	37.5	8.8	16	44.74	7.04	-0.89 [-1.60, -0.18]	5.76
Liu, 2019	6	22.8	4.65	6	38.7	4.24	-3.30 [-4.98, -1.62]	5.17
Xie, 2020	8	25.17	2.31	8	39.86	1.77	-6.75 [-9.26, -4.23]	4.47
Xie, 2020	12	25.7	2.89	12	39.6	1.58	-5.76 [-7.57, -3.96]	5.07
An, 2023	6	32.68	4.58	6	53.64	7.73	-3.05 [-4.65, -1.44]	5.23
Zhou, 2017	10	29.99	5	10	61.2	6.38	-5.22 [-7.04, -3.39]	5.06
Heterogeneity: $\tau^2 = 5.13$, $I^2 = 89.41\%$, $H^2 = 9.44$							-4.37 [-5.45, -3.29]	
Test of $\theta_i = \theta_j$: Q(20) = 131.87, p = 0.00								

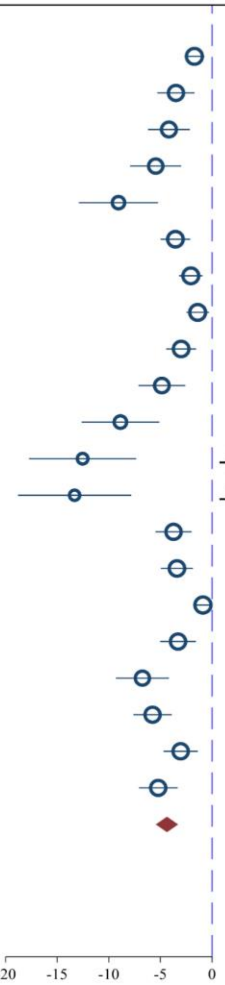

Random-effects REML model

Supplementary Figure 5 The forest plot for the effect of sevoflurane preconditioning on infarct size after myocardial I/R.

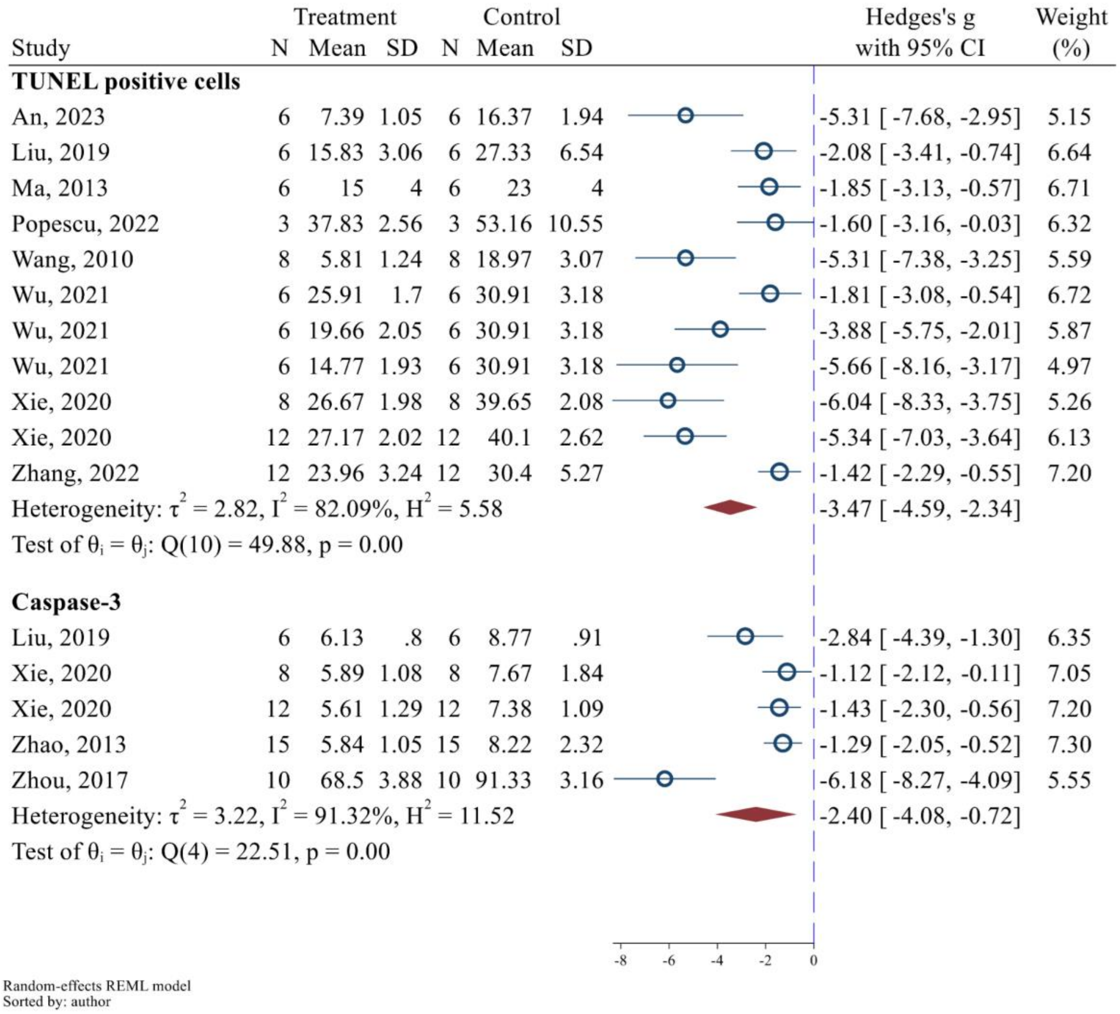

Supplementary Figure 6 The forest plot for the effect of sevoflurane preconditioning on apoptosis after myocardial I/R.

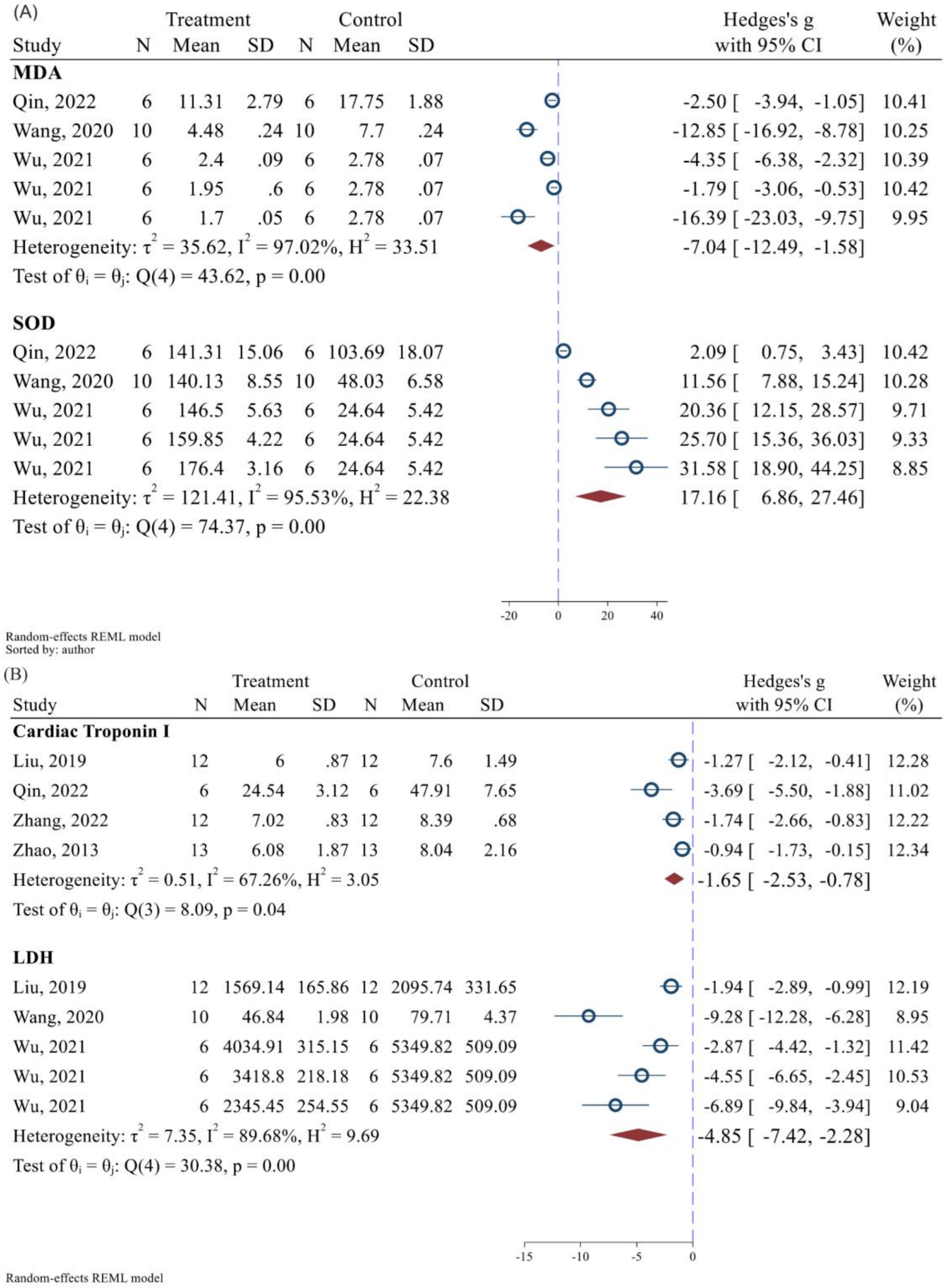

Supplementary Figure 7 The forest plot for the effect of sevoflurane preconditioning on (A) oxidative stress and (B) serum cardiac biomarker levels after myocardial I/R.

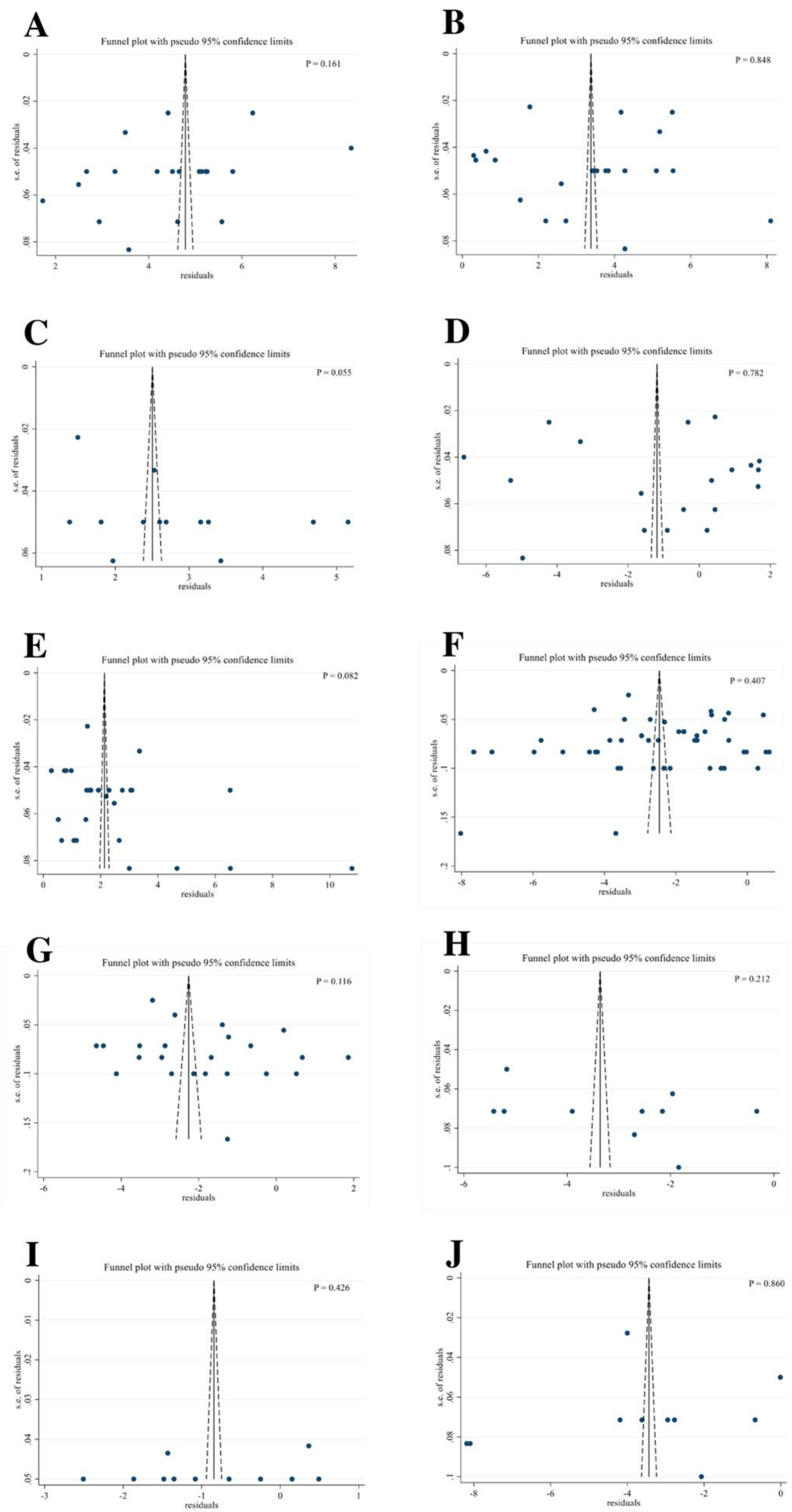

A
Funnel plot with pseudo 95% confidence limits
P = 0.161
s.e. of residuals
residuals
B
Funnel plot with pseudo 95% confidence limits
P = 0.848
s.e. of residuals
residuals
C
Funnel plot with pseudo 95% confidence limits
P = 0.055
s.e. of residuals
residuals
D
Funnel plot with pseudo 95% confidence limits
P = 0.782
s.e. of residuals
residuals
E
Funnel plot with pseudo 95% confidence limits
P = 0.082
s.e. of residuals
residuals
F
Funnel plot with pseudo 95% confidence limits
P = 0.407
s.e. of residuals
residuals
G
Funnel plot with pseudo 95% confidence limits
P = 0.116
s.e. of residuals
residuals
H
Funnel plot with pseudo 95% confidence limits
P = 0.212
s.e. of residuals
residuals
I
Funnel plot with pseudo 95% confidence limits
P = 0.426
s.e. of residuals
residuals
J
Funnel plot with pseudo 95% confidence limits
P = 0.860
s.e. of residuals
residuals

Supplementary Figure 8 Publication Bias Assessment for the Effect of Sevoflurane Post-conditioning on: (A) -dp/dt, (B) +dp/dt, (C) LVDP, (D) LVEDP, (E) HR, (F) Infarct Size, (G) TUNEL, (H) MDA, (I) Troponin-I, (J) LDH

Study	Treatment N	Treatment Mean	Treatment SD	Control N	Control Mean	Control SD	Hedges's g with 95% CI	Weight (%)
**-dp/dt**								
Geng, 2022	10	927.3	100.2	10	592.2	83.5	3.48 [2.11, 4.85]	2.21
Geng, 2022	10	1000.2	85.3	10	592.2	83.5	4.63 [2.97, 6.29]	2.05
Geng, 2022	10	926.2	80	10	592.2	83.5	3.91 [2.44, 5.39]	2.16
Geng, 2022	10	864	72.1	10	592.2	83.5	3.34 [2.01, 4.67]	2.23
Geng, 2022	10	957.2	89.1	10	592.2	83.5	4.05 [2.54, 5.56]	2.14
Geng, 2022	10	931.3	80.4	10	592.2	83.5	3.96 [2.47, 5.45]	2.15
Geng, 2022	10	843.4	76.3	10	592.2	83.5	3.01 [1.75, 4.26]	2.27
Geng, 2022	10	714.1	72.2	10	592.2	83.5	1.50 [0.54, 2.45]	2.42
Huang, 2019	15	3666.66	166.67	10	2527.77	138.89	7.04 [4.94, 9.14]	1.81
Qi, 2019	20	3982.45	350.88	20	2982.45	280.7	3.08 [2.18, 3.99]	2.44
Qi, 2019	20	4982.45	491.23	20	2982.45	280.7	4.90 [3.67, 6.13]	2.28
Qiao, 2018	8	787.5	1056.25	8	290.65	459.4	0.58 [-0.37, 1.52]	2.42
Tan, 2020	7	.69	.07	7	.58	.04	1.81 [0.62, 2.99]	2.31
Tan, 2020	7	.77	.06	7	.58	.04	3.49 [1.87, 5.11]	2.07
Tan, 2020	7	.91	.09	7	.58	.04	4.44 [2.52, 6.35]	1.91
Wu, 2021	10	3770.7	140.13	10	3171.97	152.87	3.91 [2.44, 5.38]	2.16
Yao, 2010	15	1794	173	15	1238	287	2.28 [1.38, 3.19]	2.44
Zhang, 2014	9	1198	244	9	881	218	1.30 [0.33, 2.28]	2.41
Zhang, 2020	6	1103	99	6	888	64	2.38 [0.97, 3.79]	2.19
Zhou, 2017	10	234	17	10	189	23	2.13 [1.06, 3.20]	2.37
Heterogeneity: $\tau^2 = 1.54$, $I^2 = 79.44\%$, $H^2 = 4.86$							3.13 [2.51, 3.75]	
Test of $\theta_i = \theta_j$: Q(19) = 89.26, p = 0.00								
**+dp/dt**								
Geng, 2022	10	2009.2	201.1	10	1450.1	220.3	2.54 [1.39, 3.69]	2.33
Geng, 2022	10	2296.3	198	10	1450.1	220.3	3.87 [2.41, 5.33]	2.16
Geng, 2022	10	1869.2	138.1	10	1450.1	220.3	2.18 [1.11, 3.26]	2.36
Geng, 2022	10	1858.2	94.1	10	1450.1	220.3	2.31 [1.20, 3.41]	2.35
Geng, 2022	10	1993.3	100.2	10	1450.1	220.3	3.04 [1.78, 4.30]	2.27
Geng, 2022	10	1986.1	91	10	1450.1	220.3	3.05 [1.78, 4.31]	2.27
Geng, 2022	10	1908	89.9	10	1450.1	220.3	2.61 [1.44, 3.77]	2.32
Geng, 2022	10	1843.1	92	10	1450.1	220.3	2.23 [1.14, 3.32]	2.36
Obal, 2001	11	99	23.69	11	110	49.07	-0.27 [-1.08, 0.53]	2.48
Obal, 2001	11	77	29.61	11	110	49.07	-0.78 [-1.62, 0.05]	2.47
Obal, 2001	13	82	55.19	11	110	49.07	-0.51 [-1.30, 0.27]	2.49
Obal, 2001	12	73	35.35	11	110	49.07	-0.84 [-1.67, -0.02]	2.47
Qi, 2019	20	5122.8	543.86	20	3894.73	280.7	2.78 [1.92, 3.64]	2.46
Qi, 2019	20	6000	649.12	20	3894.73	280.7	4.13 [3.04, 5.22]	2.36
Qiao, 2018	8	988.09	1234.12	8	599.2	813.48	0.35 [-0.58, 1.29]	2.43
Tan, 2020	7	.96	.09	7	.83	.06	1.59 [0.45, 2.74]	2.33
Tan, 2020	7	.91	.08	7	.83	.06	1.06 [0.00, 2.12]	2.37
Tan, 2020	7	1.68	.15	7	.83	.06	6.97 [4.21, 9.73]	1.47
Wu, 2021	10	4318.47	152.87	10	3681.53	140.13	4.16 [2.62, 5.70]	2.12
Yang, 2016	22	2283.52	1042.3	22	1632.18	1329.83	0.54 [-0.06, 1.13]	2.56
Yao, 2010	15	2741	277	15	1750	208	3.94 [2.72, 5.15]	2.29
Zhang, 2014	9	1568	398	9	1103	208	1.39 [0.40, 2.39]	2.40
Zhang, 2020	6	1457	107	6	1145	79	3.06 [1.45, 4.67]	2.08
Zhou, 2017	10	324	21	10	283	12	2.30 [1.20, 3.40]	2.35
Heterogeneity: $\tau^2 = 2.63$, $I^2 = 90.54\%$, $H^2 = 10.57$							2.03 [1.33, 2.72]	
Test of $\theta_i = \theta_j$: Q(23) = 231.17, p = 0.00								

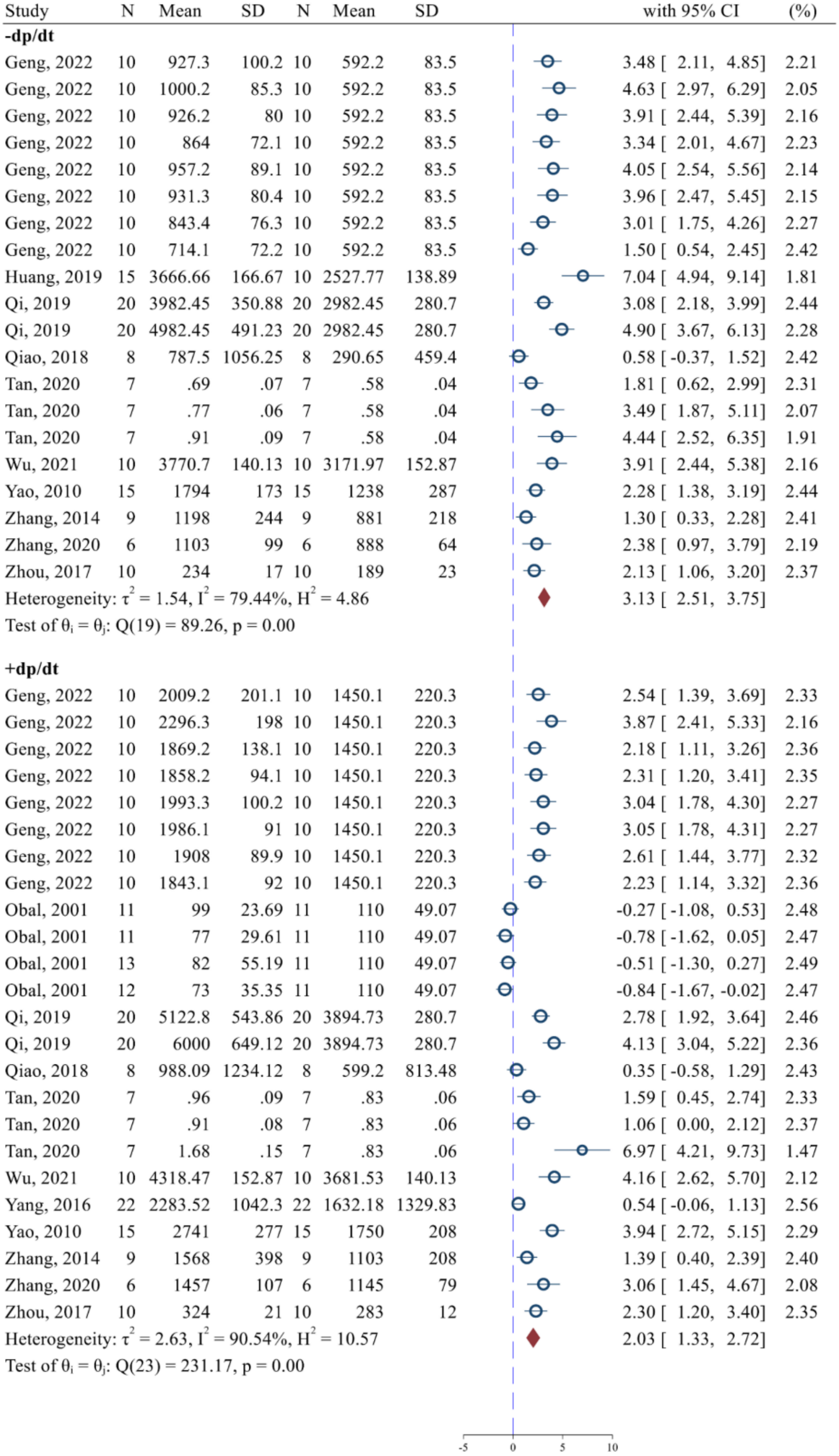

Random-effects REML model

Supplementary Figure 9 The forest plot for the effect of sevoflurane post-conditioning on maximal decline of left ventricular pressure (-dp/dt) and maximal rate of left ventricular pressure (+dp/dt) after myocardial I/R.

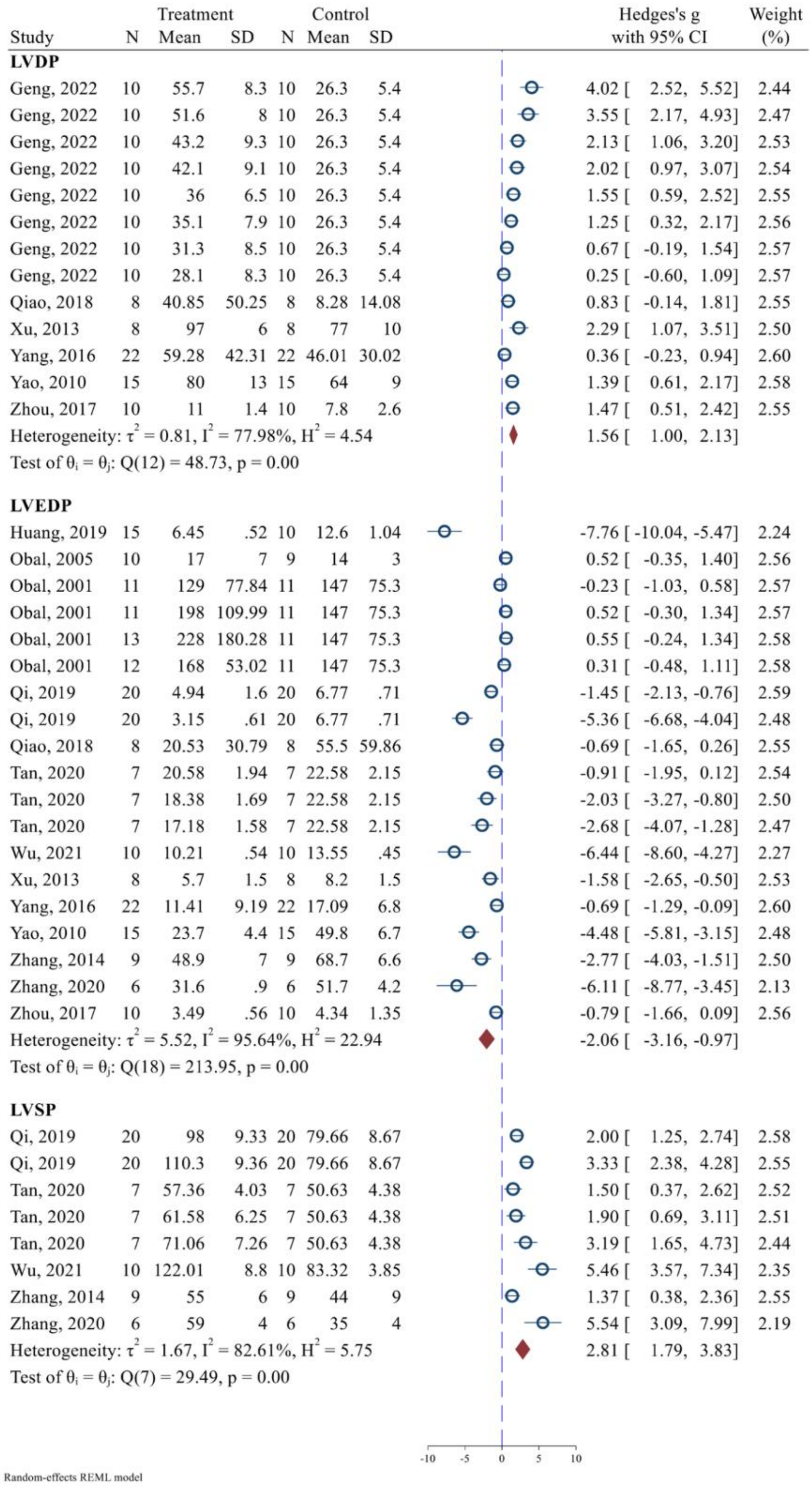

Supplementary Figure 10 The forest plot for the effect of sevoflurane post-conditioning on left ventricular diastolic pressure (LVDP), left ventricular end-diastolic pressure (LVEDP), and left ventricular shortening fraction (LVSP) after MIRI.

Study	Treatment N	Treatment Mean	Treatment SD	Control N	Control Mean	Control SD	Hedges's g with 95% CI	Weight (%)
**LVIDd**								
Wu, 2022	10	5.5	.67	10	7.59	.85	-2.62 [-3.78, -1.45]	4.84
Yu, 2014	10	6.74	.44	10	7.69	.69	-1.57 [-2.54, -0.60]	4.88
Zhang, 2014	10	6.78	.38	10	7.76	.77	-1.55 [-2.51, -0.58]	4.89
Zhang, 2020	8	7.48	.15	8	8.5	.33	-3.76 [-5.36, -2.16]	4.73
Heterogeneity: $\tau^2 = 0.51$, $I^2 = 60.65\%$, $H^2 = 2.54$							-2.22 [-3.13, -1.31]	
Test of $\theta_i = \theta_j$: Q(3) = 7.28, p = 0.06								
**LVIDs**								
Wu, 2022	10	4.41	.48	10	6.09	.66	-2.79 [-3.99, -1.58]	4.83
Yu, 2014	10	5.25	.32	10	6.32	.74	-1.80 [-2.81, -0.79]	4.88
Zhang, 2014	10	5.17	.19	10	6.38	.55	-2.82 [-4.03, -1.61]	4.83
Zhang, 2020	8	5.54	.23	8	6.36	.3	-2.90 [-4.27, -1.53]	4.79
Heterogeneity: $\tau^2 = 0.02$, $I^2 = 5.40\%$, $H^2 = 1.06$							-2.48 [-3.09, -1.88]	
Test of $\theta_i = \theta_j$: Q(3) = 2.67, p = 0.44								
**LVEF**								
Huang, 2019	15	47.83	2.06	10	32.16	1.65	7.93 [5.60, 10.26]	4.47
Ru, 2021	3	52.25	5.05	3	38.02	3.96	2.51 [0.60, 4.42]	4.62
Song, 2022	18	51.45	5.41	18	25.8	2.96	5.75 [4.28, 7.23]	4.76
Wu, 2022	10	53.04	4.71	10	33.6	3.4	4.53 [2.90, 6.17]	4.72
Yu, 2014	10	44.72	4.19	10	31.47	1.71	3.97 [2.48, 5.45]	4.76
Zhang, 2014	10	46.02	5.5	10	34.53	3.82	2.32 [1.22, 3.43]	4.86
Zhang, 2020	8	46.56	4.34	8	31.07	1.49	4.51 [2.70, 6.33]	4.66
Heterogeneity: $\tau^2 = 2.55$, $I^2 = 79.41\%$, $H^2 = 4.86$							4.40 [3.06, 5.74]	
Test of $\theta_i = \theta_j$: Q(6) = 28.22, p = 0.00								
**LVFS**								
Huang, 2019	15	34.48	2.76	10	23.79	1.03	4.60 [3.11, 6.09]	4.76
Ru, 2021	3	34.84	3.39	3	22.26	2.52	3.37 [1.07, 5.67]	4.48
Song, 2022	18	27.14	2.3	18	13.25	1.39	7.15 [5.38, 8.92]	4.67
Yu, 2014	10	23.43	3.2	10	16.83	2.6	2.17 [1.09, 3.24]	4.86
Zhang, 2014	10	24.1	3.66	10	17.38	2.26	2.12 [1.05, 3.18]	4.86
Zhang, 2020	8	23.3	4.31	8	15.98	2.12	2.04 [0.87, 3.20]	4.84
Heterogeneity: $\tau^2 = 3.38$, $I^2 = 87.69\%$, $H^2 = 8.12$							3.49 [1.90, 5.09]	
Test of $\theta_i = \theta_j$: Q(5) = 33.16, p = 0.00								

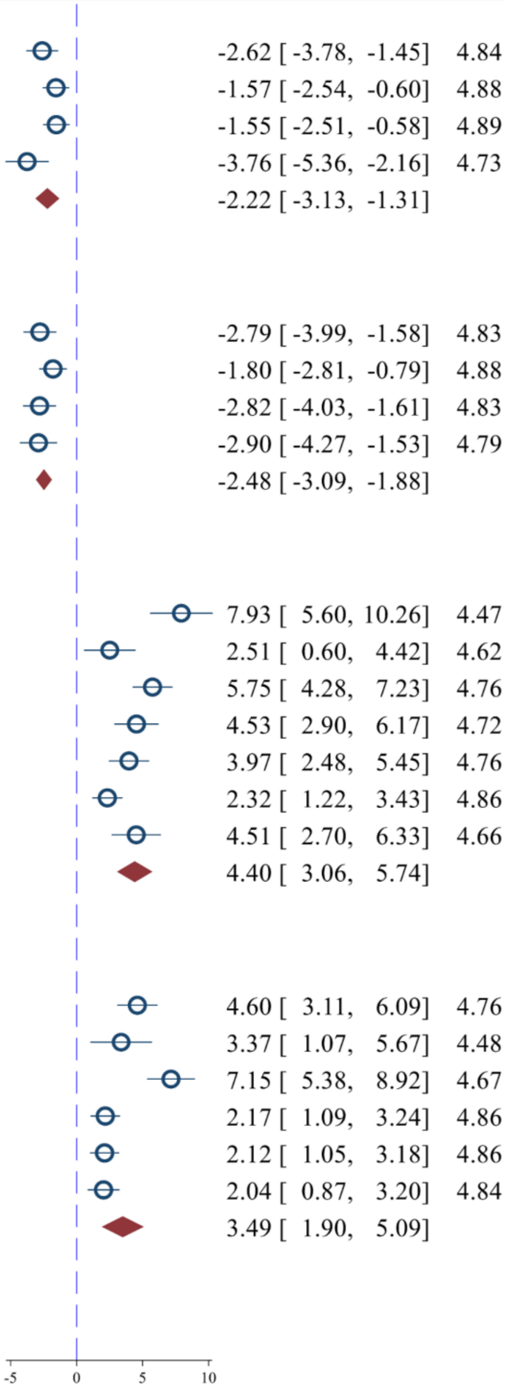

Random-effects REML model

Supplementary Figure 11 The forest plot for the effect of sevoflurane post-conditioning on left ventricular internal diameter at end-diastole (LVIDd), left ventricular internal diameter at end-systole (LVIDs), left ventricular ejection fraction (LVEF), and left ventricular fraction shortening (LVFS) after myocardial I/R injury.

Study	Treatment N	Treatment Mean	Treatment SD	Control N	Control Mean	Control SD	Hedges's g with 95% CI	Weight (%)
**HR**								
Gao, 2021	7	66	5.4	7	59.3	2.3	1.51 [0.38, 2.64]	3.59
Gao, 2016	7	365	8	7	365	12	0.00 [-0.98, 0.98]	3.72
Geng, 2022	10	275.2	20.9	10	241.3	12	1.91 [0.88, 2.93]	3.68
Geng, 2022	10	269.2	15.3	10	241.3	12	1.94 [0.91, 2.98]	3.67
Geng, 2022	10	263.2	14	10	241.3	12	1.61 [0.63, 2.59]	3.72
Geng, 2022	10	258.3	16.1	10	241.3	12	1.15 [0.24, 2.06]	3.78
Geng, 2022	10	248.1	13.5	10	241.3	12	0.51 [-0.34, 1.36]	3.83
Geng, 2022	10	250.2	10	10	241.3	12	0.77 [-0.10, 1.64]	3.81
Geng, 2022	10	246.3	14.1	10	241.3	12	0.37 [-0.48, 1.21]	3.83
Geng, 2022	10	247.1	12	10	241.3	12	0.46 [-0.39, 1.31]	3.83
Huhn, 2008	11	405	33	9	380	28	0.78 [-0.10, 1.65]	3.81
Li, 2015	12	346	14	12	352	18	-0.36 [-1.14, 0.42]	3.88
Li, 2015	12	337	15	12	352	18	-0.87 [-1.69, -0.06]	3.86
Li, 2015	12	316	17	12	319	15	-0.18 [-0.95, 0.59]	3.89
Li, 2015	12	313	13	12	319	15	-0.41 [-1.19, 0.37]	3.88
Obal, 2005	10	397	23	9	371	25	1.04 [0.12, 1.96]	3.77
Redel, 2006	8	402	67.88	8	439	36.77	-0.64 [-1.59, 0.31]	3.74
Tai, 2012	7	77.7	3.6	7	80.9	7.7	-0.50 [-1.50, 0.50]	3.71
Tosaka, 2011	7	394	34	7	397	27	-0.09 [-1.07, 0.89]	3.72
Xu, 2013	8	313	39	8	299	41	0.33 [-0.60, 1.26]	3.76
Yang, 2016	22	270.41	56.19	22	249.43	49.2	0.39 [-0.20, 0.98]	4.02
Yao, 2010	15	249	18	15	174	43	2.21 [1.32, 3.11]	3.79
Yu, 2014	6	185	11.3	6	132	6.1	5.39 [2.99, 7.78]	2.37
Zhang, 2014	6	246	10	6	125	13	9.63 [5.64, 13.62]	1.34
Zhang, 2014	9	216	47	9	158	35	1.33 [0.35, 2.31]	3.72
Zhang, 2020	6	182	16	6	128	12	3.52 [1.77, 5.28]	2.97
Zhang, 2020	6	156	19	6	124	12	1.86 [0.58, 3.14]	3.44
Zhou, 2017	10	263	17	10	173	15	5.38 [3.51, 7.24]	2.86
Heterogeneity: $\tau^2 = 1.93$, $I^2 = 89.17\%$, $H^2 = 9.23$							1.07 [0.51, 1.63]	
Test of $\theta_i = \theta_j$: Q(27) = 155.59, p = 0.00								

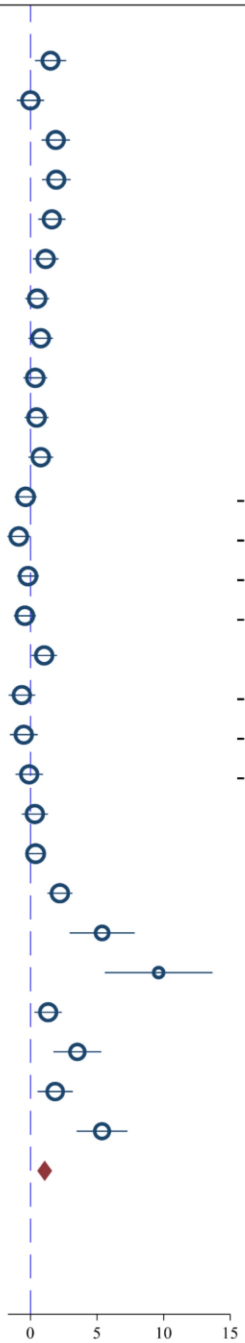

Random-effects REML model

Supplementary Figure 12 The forest plot for the effect of sevoflurane post-conditioning on heart rate (HR) after myocardial I/R injury.

Study	Treatment N	Treatment Mean	Treatment SD	Control N	Control Mean	Control SD	Hedges's g with 95% CI	Weight (%)
**Infarct size**								
Cao, 2015	6	25.67	5.52	6	51.07	2.27	-5.56 [-8.01, -3.10]	1.67
Deng, 2022	5	18.36	2.62	5	40.73	5.51	-4.68 [-7.02, -2.35]	1.73
Gao, 2021	7	26.61	1.29	7	36.77	2.58	-4.66 [-6.65, -2.68]	1.92
Gao, 2016	7	26.5	3.4	7	36.9	7.6	-1.65 [-2.81, -0.50]	2.36
Geng, 2022	5	20	2.8	5	44	5.8	-4.76 [-7.13, -2.39]	1.71
Geng, 2022	5	23	5.7	5	44	5.8	-3.30 [-5.13, -1.47]	2.00
Geng, 2022	5	22	4.7	5	44	5.8	-3.76 [-5.76, -1.77]	1.91
Geng, 2022	5	25	3.9	5	44	5.8	-3.47 [-5.36, -1.58]	1.97
Geng, 2022	5	32	5.8	5	44	5.8	-1.87 [-3.26, -0.48]	2.24
Geng, 2022	5	30	5.8	5	44	5.8	-2.18 [-3.65, -0.71]	2.20
Geng, 2022	5	34	4.2	5	44	5.8	-1.78 [-3.15, -0.42]	2.25
Geng, 2022	5	39	4.9	5	44	5.8	-0.84 [-2.02, 0.34]	2.35
Huang, 2019	15	32.26	1.52	10	45.04	3.11	-5.42 [-7.12, -3.73]	2.08
Huhn, 2008	11	32.7	12.8	9	51.4	5	-1.77 [-2.78, -0.77]	2.43
Li, 2013	6	34	2	6	56	5	-5.33 [-7.71, -2.96]	1.71
Li, 2013	6	45	3	6	47	4	-0.52 [-1.59, 0.54]	2.40
Li, 2015	6	34	3	6	58	5	-5.37 [-7.76, -2.98]	1.70
Li, 2015	6	32	2	6	58	5	-6.30 [-9.03, -3.57]	1.53
Li, 2015	6	45	3	6	47	3	-0.62 [-1.69, 0.46]	2.40
Li, 2015	6	43	3	6	47	3	-1.23 [-2.39, -0.08]	2.36
Lin, 2016	6	28.9	11.76	6	39.6	2.45	-1.16 [-2.31, -0.02]	2.36
Obal, 2005	10	18.21	4.74	9	48.85	11.28	-3.45 [-4.85, -2.06]	2.24
Obal, 2001	11	33	7	11	38	7	-0.69 [-1.52, 0.14]	2.50
Obal, 2001	11	23	6.5	11	38	7	-2.14 [-3.16, -1.11]	2.42
Obal, 2001	13	23	6.5	11	38	7	-2.15 [-3.14, -1.17]	2.44
Obal, 2001	12	23	10	11	38	7	-1.66 [-2.58, -0.74]	2.46
Obal, 2003	8	15	10.1	7	51	5.4	-4.10 [-5.85, -2.35]	2.05
Obal, 2003	8	26	11.54	7	51	5.4	-2.55 [-3.87, -1.23]	2.28
Obal, 2003	7	26	11.47	7	51	5.4	-2.61 [-3.99, -1.23]	2.25
Qi, 2019	20	23.17	3.37	20	39.81	3.92	-4.46 [-5.61, -3.31]	2.36
Qiao, 2018	8	38.71	4.78	8	56.23	6.44	-2.92 [-4.29, -1.55]	2.25
Qiao, 2018	8	25.08	5.05	8	35.73	3.49	-2.32 [-3.55, -1.09]	2.32
Redel, 2006	8	14.9	2.83	8	50.4	15.27	-3.06 [-4.46, -1.65]	2.23
Ru, 2021	3	21.68	2.4	3	41.8	4.07	-4.82 [-7.83, -1.81]	1.40
Song, 2022	6	12.89	1.77	6	52.89	5.67	-8.79 [-12.46, -5.12]	1.14
Stumpner, 2014	7	17	4	7	44	9	-3.63 [-5.29, -1.97]	2.09
Tai, 2012	7	34.9	5.6	7	53.4	7.6	-2.59 [-3.97, -1.22]	2.25
Tan, 2020	7	33.95	4.46	7	47.91	5.75	-2.54 [-3.90, -1.18]	2.26
Tan, 2020	7	23.45	3.02	7	47.91	5.75	-4.99 [-7.08, -2.89]	1.86
Tan, 2020	7	16.11	2.01	7	47.91	5.75	-6.91 [-9.65, -4.17]	1.52
Tosaka, 2011	7	17	6	7	42	6	-3.90 [-5.65, -2.15]	2.05
Xu, 2013	8	27	6	8	44	5	-2.91 [-4.28, -1.54]	2.25
Yao, 2010	10	16.8	4.5	10	36.8	5.4	-3.85 [-5.31, -2.39]	2.20
Yu, 2021	3	19.58	1.05	3	30.91	.92	-9.18 [-14.53, -3.83]	0.69
Yu, 2014	6	17.13	4.19	6	39.57	3.47	-5.38 [-7.78, -2.99]	1.70
Zhang, 2014	6	21.1	4.7	6	44.2	3.1	-5.36 [-7.74, -2.97]	1.70
Zhang, 2014	6	20.09	2.67	6	50.96	3.72	-8.80 [-12.47, -5.13]	1.14
Zhang, 2020	6	31.51	2.09	6	48.26	1.61	-8.29 [-11.76, -4.81]	1.21
Zhang, 2020	6	30.96	1.81	6	46.98	2.32	-7.11 [-10.14, -4.08]	1.39
Zhou, 2017	10	31.55	6.03	10	61.2	6.38	-4.57 [-6.22, -2.93]	2.10
Heterogeneity: $\tau^2 = 2.59$, $I^2 = 81.93\%$, $H^2 = 5.53$							-3.42 [-3.93, -2.90]	
Test of $\theta_i = \theta_j$: Q(49) = 229.07, p = 0.00								

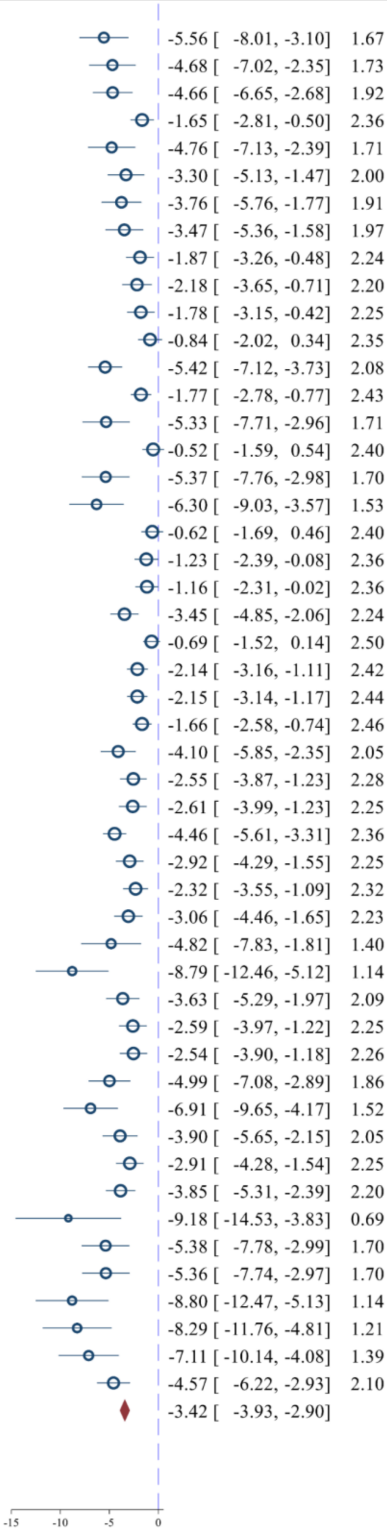

Random-effects REML model

Supplementary Figure 13 The forest plot for the effect of sevoflurane post-conditioning on infarct size after myocardial I/R injury.

Study	Treatment N	Treatment Mean	Treatment SD	Control N	Control Mean	Control SD	Hedges's g with 95% CI	Weight (%)
**TUNEL positive cells**								
Drenger, 2011	8	9.1	4.6	10	13.7	4.7	-0.94 [-1.88, -0.00]	3.06
Gao, 2021	7	17.36	1.21	7	25.27	1.43	-5.59 [-7.88, -3.30]	2.77
Gao, 2016	7	18.56	1.08	7	25.38	1.61	-4.66 [-6.64, -2.67]	2.85
Geng, 2022	5	8.2	1.21	5	13.97	1.9	-3.27 [-5.09, -1.45]	2.89
Geng, 2022	5	6.86	1.41	5	13.97	1.9	-3.84 [-5.86, -1.82]	2.84
Geng, 2022	5	8.34	1.51	5	13.97	1.9	-2.96 [-4.68, -1.25]	2.92
Geng, 2022	5	12.61	2.1	5	13.97	1.9	-0.61 [-1.76, 0.54]	3.03
Geng, 2022	5	11.07	1.87	5	13.97	1.9	-1.39 [-2.66, -0.12]	3.00
Huang, 2019	15	25.93	2.91	10	37.52	3.11	-3.75 [-5.04, -2.45]	3.00
Li, 2015	6	8	1	6	15	2	-4.09 [-6.03, -2.15]	2.86
Li, 2015	6	7	1	6	15	2	-4.67 [-6.81, -2.53]	2.81
Li, 2015	6	28	3	6	26	2	0.72 [-0.36, 1.81]	3.04
Li, 2015	6	25	2	6	26	2	-0.46 [-1.52, 0.60]	3.04
Qi, 2019	20	21.03	2.3	20	35.51	4.03	-4.33 [-5.45, -3.20]	3.03
Ru, 2021	3	27.71	3.14	3	38.71	4.14	-2.40 [-4.26, -0.53]	2.88
Tan, 2020	7	27.22	3.23	7	34.32	4.13	-1.79 [-2.98, -0.61]	3.02
Tan, 2020	7	20	2.32	7	34.32	4.13	-4.00 [-5.78, -2.22]	2.90
Tan, 2020	7	14.19	2.06	7	34.32	4.13	-5.77 [-8.13, -3.42]	2.76
Wu, 2022	5	11.27	1.27	5	21.46	2.12	-5.27 [-7.83, -2.70]	2.70
Xu, 2013	8	13	4	8	23	4	-2.36 [-3.60, -1.13]	3.01
Yao, 2010	10	14.38	3.68	10	27.88	6.24	-2.52 [-3.67, -1.38]	3.03
Yu, 2021	5	7.83	.98	5	10.22	.82	-2.39 [-3.92, -0.86]	2.96
Zhang, 2014	6	13.6	4.2	6	25.8	3.8	-2.81 [-4.35, -1.28]	2.95
Heterogeneity: $\tau^2 = 2.32$, $I^2 = 81.31\%$, $H^2 = 5.35$							-2.83 [-3.54, -2.12]	
Test of $\theta_i = \theta_j$: Q(22) = 120.49, p = 0.00								
**BCL-2**								
Cao, 2015	5	68.33	2.43	5	31.24	3.93	10.25 [5.62, 14.88]	2.06
Deng, 2022	5	.47	.06	5	.21	.1	2.85 [1.17, 4.52]	2.92
Qi, 2019	20	.58	.07	20	.37	.03	3.82 [2.79, 4.86]	3.04
Qi, 2019	20	.62	.06	20	.37	.03	5.17 [3.88, 6.45]	3.00
Yu, 2014	4	.51	.04	4	.2	.02	8.52 [4.18, 12.87]	2.15
Heterogeneity: $\tau^2 = 5.24$, $I^2 = 86.82\%$, $H^2 = 7.58$							5.40 [3.09, 7.72]	
Test of $\theta_i = \theta_j$: Q(4) = 15.41, p = 0.00								
**Caspase-3**								
Cao, 2015	5	19.13	6.02	5	49.05	4.66	-5.02 [-7.49, -2.55]	2.72
Deng, 2022	5	.36	.06	5	.91	.07	-7.62 [-11.14, -4.10]	2.41
Tan, 2020	7	1.25	.12	7	1.47	.17	-1.40 [-2.51, -0.29]	3.03
Tan, 2020	7	1.03	.1	7	1.47	.17	-2.95 [-4.42, -1.48]	2.97
Tan, 2020	7	.96	.11	7	1.47	.17	-3.33 [-4.91, -1.76]	2.95
Zeng, 2022	3	1.59	.27	3	5.43	.82	-5.03 [-8.15, -1.91]	2.53
Zhou, 2017	10	64.85	5.35	10	91.33	3.16	-5.77 [-7.75, -3.80]	2.85
Heterogeneity: $\tau^2 = 2.87$, $I^2 = 76.59\%$, $H^2 = 4.27$							-4.09 [-5.59, -2.60]	
Test of $\theta_i = \theta_j$: Q(6) = 26.22, p = 0.00								

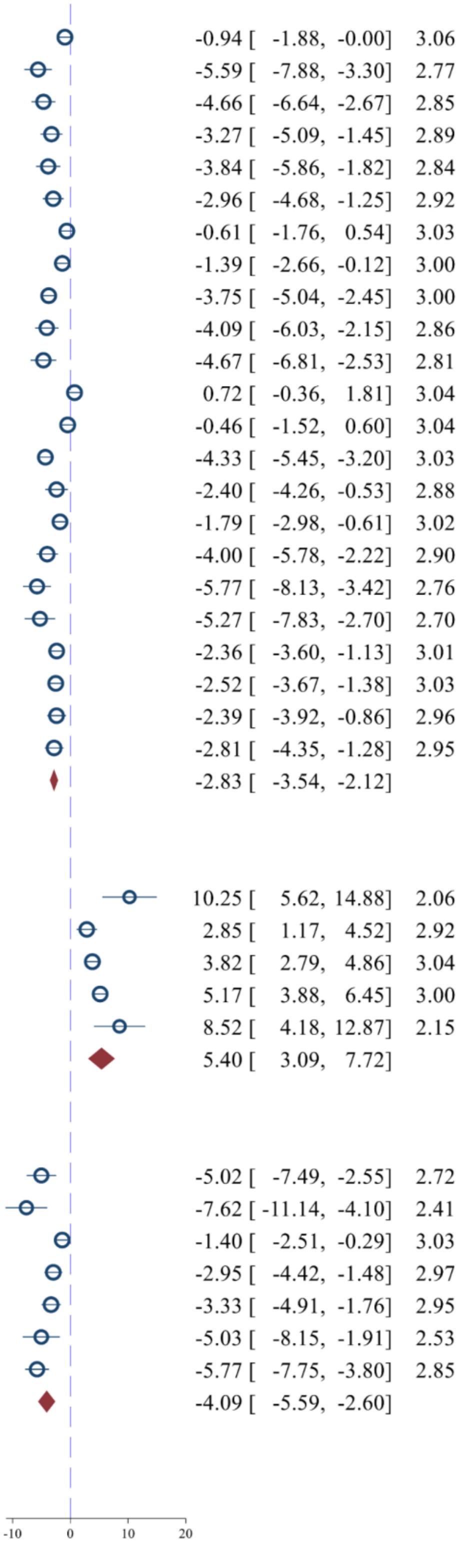

Random-effects REML model

Supplementary Figure 14 The forest plot for the effect of sevoflurane post-conditioning on apoptosis after myocardial I/R injury.

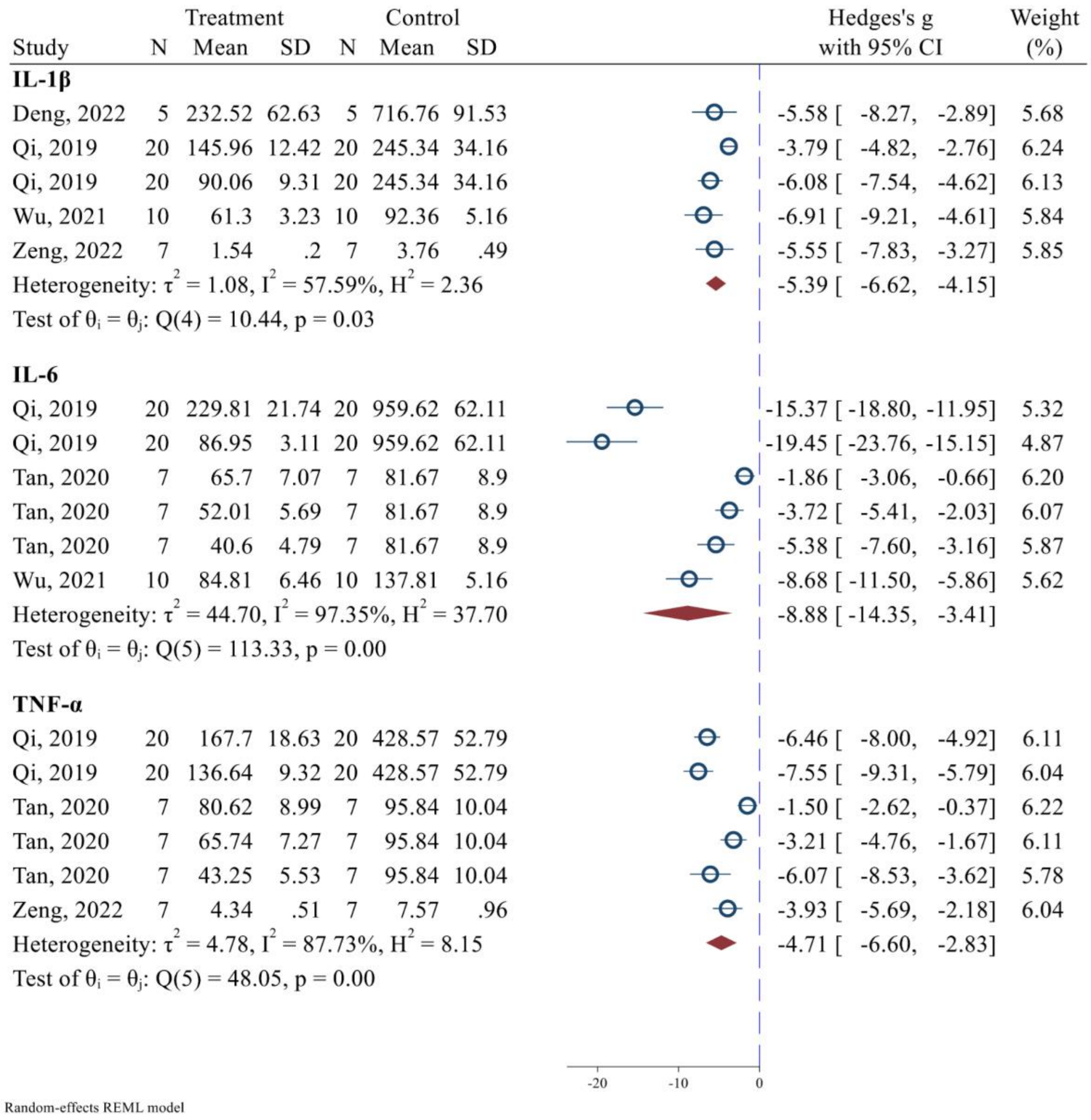

Supplementary Figure 15 The forest plot for the effect of sevoflurane post-conditioning on inflammation after myocardial I/R injury.

Study	Treatment N	Treatment Mean	Treatment SD	Control N	Control Mean	Control SD	Hedges's g with 95% CI	Weight (%)
**GSH**								
Tan, 2020	7	6.79	.86	7	4.69	.64	2.59 [1.22, 3.97]	4.29
Tan, 2020	7	7.47	1.01	7	4.69	.64	3.08 [1.57, 4.58]	4.27
Tan, 2020	7	12.9	1.18	7	4.69	.64	8.10 [4.94, 11.25]	4.00
Yu, 2014	6	13.25	.45	6	6.45	.13	18.95 [11.30, 26.61]	2.86
Zeng, 2022	7	538.02	83.46	7	341.54	50	2.67 [1.28, 4.07]	4.29
Heterogeneity: $\tau^2 = 30.19$, $I^2 = 97.05\%$, $H^2 = 33.95$							6.23 [1.17, 11.29]	
Test of $\theta_i = \theta_j$: Q(4) = 26.81, p = 0.00								
**MDA**								
Gao, 2021	7	15	.53	7	18.71	.53	-6.55 [-9.17, -3.94]	4.11
Gao, 2016	7	15.14	.52	7	19.48	.74	-6.35 [-8.90, -3.80]	4.12
Tan, 2020	7	55.74	6.48	7	66.66	7.41	-1.47 [-2.59, -0.35]	4.31
Tan, 2020	7	41.48	5.18	7	66.66	7.41	-3.69 [-5.37, -2.01]	4.25
Tan, 2020	7	34.81	3.89	7	66.66	7.41	-5.04 [-7.15, -2.93]	4.19
Wu, 2022	5	2.91	.36	5	4.4	.53	-2.97 [-4.69, -1.25]	4.25
Wu, 2021	10	3.1	.23	10	4.56	.21	-6.35 [-8.49, -4.21]	4.19
Yu, 2021	8	6.51	1.12	8	10.17	1.11	-3.10 [-4.52, -1.68]	4.28
Yu, 2014	6	6.55	.54	6	8.73	.51	-3.83 [-5.69, -1.98]	4.23
Zeng, 2022	7	8.89	.56	7	14.58	2.22	-3.29 [-4.85, -1.73]	4.27
Heterogeneity: $\tau^2 = 1.98$, $I^2 = 71.57\%$, $H^2 = 3.52$							-4.03 [-5.09, -2.98]	
Test of $\theta_i = \theta_j$: Q(9) = 32.40, p = 0.00								
**SOD**								
Gao, 2016	7	102.8	2.1	7	91.57	3.16	3.92 [2.17, 5.67]	4.24
Qi, 2019	20	4.7	.77	20	1.6	.18	5.43 [4.10, 6.77]	4.29
Qi, 2019	20	6.87	.88	20	1.6	.18	8.13 [6.25, 10.02]	4.23
Tan, 2020	7	17.33	1.89	7	13.33	1.78	2.04 [0.80, 3.28]	4.30
Tan, 2020	7	21.22	2.33	7	13.33	1.78	3.56 [1.92, 5.21]	4.26
Tan, 2020	7	29.33	3.11	7	13.33	1.78	5.91 [3.51, 8.31]	4.15
Wu, 2021	10	116.28	8.53	10	54.26	3.88	8.96 [6.06, 11.87]	4.05
Yu, 2021	8	25.65	2.04	8	19.41	2.24	2.75 [1.42, 4.08]	4.29
Zeng, 2022	7	43.65	8.74	7	20.89	3.33	3.22 [1.68, 4.77]	4.27
Heterogeneity: $\tau^2 = 4.41$, $I^2 = 86.43\%$, $H^2 = 7.37$							4.72 [3.22, 6.21]	
Test of $\theta_i = \theta_j$: Q(8) = 50.69, p = 0.00								

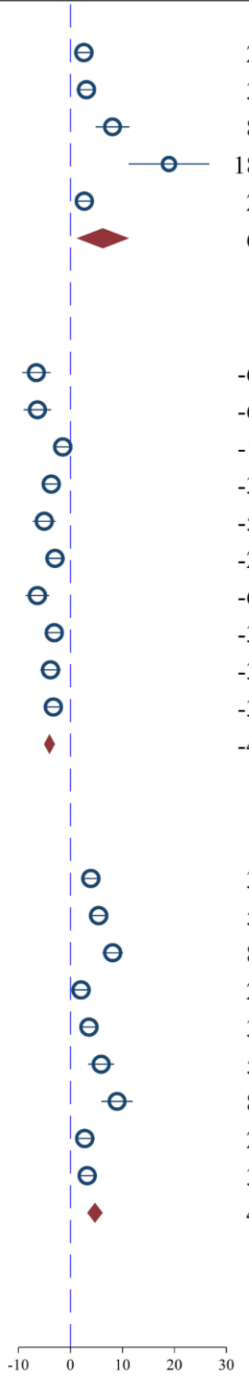

Random-effects REML model

Supplementary Figure 16 The forest plot for the effect of sevoflurane post-conditioning on oxidative stress after myocardial I/R injury.

Study	Treatment N	Treatment Mean	Treatment SD	Control N	Control Mean	Control SD	Hedges's g with 95% CI	Weight (%)
**CK-MB**								
Gao, 2021	7	18.2	1.31	7	34.92	1.97	-9.36 [-12.96, -5.75]	2.10
Gao, 2016	7	23.37	3.38	7	35	1.21	-4.29 [-6.16, -2.42]	3.26
Lin, 2016	12	1581.6	884.04	12	1569.03	695.49	0.02 [-0.76, 0.79]	3.91
Song, 2022	18	296.76	33.61	18	576.11	53.44	-6.12 [-7.67, -4.57]	3.47
Tan, 2020	7	601.52	58.02	7	680.91	70.23	-1.15 [-2.22, -0.08]	3.77
Tan, 2020	7	580.15	58.01	7	680.91	70.23	-1.46 [-2.59, -0.34]	3.74
Tan, 2020	7	519.08	54.96	7	680.91	70.23	-2.40 [-3.73, -1.08]	3.62
Yao, 2010	10	49.83	16.08	10	100.96	25.08	-2.32 [-3.43, -1.22]	3.75
Heterogeneity: $\tau^2 = 6.67$, $I^2 = 94.30\%$, $H^2 = 17.54$							-3.11 [-4.99, -1.23]	
Test of $\theta_i = \theta_j$: Q(7) = 78.13, p = 0.00								
**Cardiac Troponin I**								
Geng, 2022	10	.08	.02	10	.15	.03	-2.63 [-3.80, -1.46]	3.71
Geng, 2022	10	.07	.02	10	.15	.03	-3.01 [-4.26, -1.75]	3.66
Geng, 2022	10	.09	.02	10	.15	.03	-2.25 [-3.35, -1.16]	3.76
Geng, 2022	10	.08	.02	10	.15	.03	-2.63 [-3.80, -1.46]	3.71
Geng, 2022	10	.11	.03	10	.15	.03	-1.28 [-2.21, -0.35]	3.84
Geng, 2022	10	.1	.02	10	.15	.03	-1.88 [-2.90, -0.86]	3.79
Geng, 2022	10	.12	.03	10	.15	.03	-0.96 [-1.85, -0.07]	3.86
Geng, 2022	10	.13	.03	10	.15	.03	-0.64 [-1.50, 0.22]	3.87
Lin, 2016	12	.87	.83	12	2.5	2.77	-0.77 [-1.57, 0.03]	3.90
Yao, 2010	10	4.5	1.18	10	9.2	1.29	-3.64 [-5.05, -2.23]	3.57
Zhang, 2014	11	19.3	2.6	12	28.7	4.2	-2.57 [-3.65, -1.48]	3.76
Heterogeneity: $\tau^2 = 0.68$, $I^2 = 71.22\%$, $H^2 = 3.47$							-1.93 [-2.51, -1.35]	
Test of $\theta_i = \theta_j$: Q(10) = 34.32, p = 0.00								
**LDH**								
Gao, 2016	7	6480.44	178.77	7	7664.8	201.12	-5.83 [-8.20, -3.46]	2.90
Song, 2022	18	233.23	27.85	18	432.61	45.57	-5.16 [-6.52, -3.81]	3.60
Tan, 2020	7	126.77	13.38	7	155.9	16.54	-1.81 [-3.00, -0.62]	3.70
Tan, 2020	7	94.48	11.03	7	155.9	16.54	-4.09 [-5.90, -2.29]	3.30
Tan, 2020	7	87.4	9.45	7	155.9	16.54	-4.76 [-6.78, -2.74]	3.15
Wu, 2022	5	1536.64	166.67	5	2255.3	217.43	-3.35 [-5.20, -1.50]	3.27
Yao, 2010	10	229.6	74.73	10	330.32	89.89	-1.17 [-2.08, -0.25]	3.85
Zeng, 2022	7	49.49	6.81	7	97.23	14.66	-3.91 [-5.66, -2.16]	3.34
Zhang, 2020	6	1910.31	26.9	6	3201.8	174.88	-9.53 [-13.48, -5.58]	1.91
Zhang, 2020	6	1819.44	83.33	6	3166.66	166.67	-9.44 [-13.36, -5.52]	1.93
Heterogeneity: $\tau^2 = 4.62$, $I^2 = 86.56\%$, $H^2 = 7.44$							-4.46 [-5.95, -2.96]	
Test of $\theta_i = \theta_j$: Q(9) = 60.50, p = 0.00								

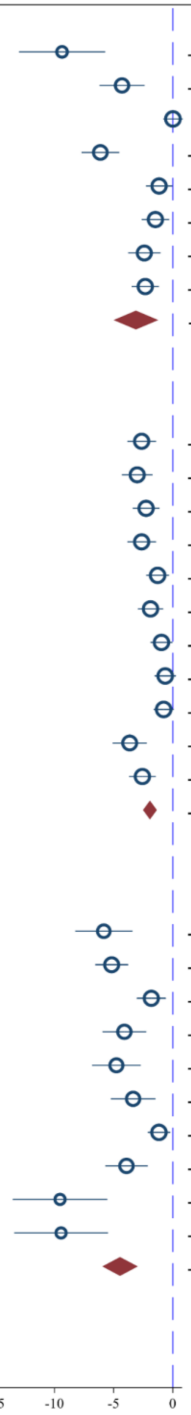

Random-effects REML model

Supplementary Figure 17 The forest plot for the effect of sevoflurane post-conditioning on cardiac biomarker levels after myocardial I/R injury.

Supplementary Table 1: PRISMA checklist

Section and Topic	Item #	Checklist item	Location where item is reported
**TITLE**			
Title	1	Identify the report as a systematic review.	Title page
**ABSTRACT**			
Abstract	2	See the PRISMA 2020 for Abstracts checklist.	Abstract
**INTRODUCTION**			
Rationale	3	Describe the rationale for the review in the context of existing knowledge.	All 3 paragraphs of the introduction, page 2
Objectives	4	Provide an explicit statement of the objective(s) or question(s) the review addresses.	The last 2 sentences of the introduction, page 2 & 3
**METHODS**			
Eligibility criteria	5	Specify the inclusion and exclusion criteria for the review and how studies were grouped for the syntheses.	Methods, Inclusion and exclusion, page 3
Information sources	6	Specify all databases, registers, websites, organisations, reference lists and other sources searched or consulted to identify studies. Specify the date when each source was last searched or consulted.	Methods, Study design, 2nd paragraph, page 3
Search strategy	7	Present the full search strategies for all databases, registers and websites, including any filters and limits used.	Supplementary Material 1
Selection process	8	Specify the methods used to decide whether a study met the inclusion criteria of the review, including how many reviewers screened each record and each report retrieved, whether they worked independently, and if applicable, details of automation tools used in the process.	Methods, Data gathering, first paragraph, page 3 & 4
Data collection process	9	Specify the methods used to collect data from reports, including how many reviewers collected data from each report, whether they worked independently, any processes for obtaining or confirming data from study investigators, and if applicable, details of automation tools used in the process.	Methods, Data gathering, first paragraph, page 3 & 4
Data items	10a	List and define all outcomes for which data were sought. Specify whether all results that were compatible with each outcome domain in each study were sought (e.g. for all measures, time points, analyses), and if not, the methods used to decide which results to collect.	Methods, Outcomes, page 4 & 5
	10b	List and define all other variables for which data were sought (e.g. participant and intervention characteristics, funding sources). Describe any assumptions made about any missing or unclear information.	Methods, Data gathering, page 4
Study risk of bias	11	Specify the methods used to assess risk of bias in the included studies, including details of the tool(s) used, how many reviewers assessed each study and whether they worked independently, and if applicable, details of automation tools used in the process.	Methods, Risk of bias assessment and

Section and Topic	Item #	Checklist item	Location where item is reported
assessment			certainty of evidence, page 5
Effect measures	12	Specify for each outcome the effect measure(s) (e.g. risk ratio, mean difference) used in the synthesis or presentation of results.	Methods, Statistical analysis, 2nd paragraph, page 5
Synthesis methods	13a	Describe the processes used to decide which studies were eligible for each synthesis (e.g. tabulating the study intervention characteristics and comparing against the planned groups for each synthesis (item #5)).	Methods, Statistical analysis, page 5
	13b	Describe any methods required to prepare the data for presentation or synthesis, such as handling of missing summary statistics, or data conversions.	Methods, Statistical analysis, page 5
	13c	Describe any methods used to tabulate or visually display results of individual studies and syntheses.	Methods, Statistical analysis, page 5
	13d	Describe any methods used to synthesize results and provide a rationale for the choice(s). If meta-analysis was performed, describe the model(s), method(s) to identify the presence and extent of statistical heterogeneity, and software package(s) used.	Methods, Statistical analysis, page 5
	13e	Describe any methods used to explore possible causes of heterogeneity among study results (e.g. subgroup analysis, meta-regression).	Methods, Statistical analysis, page 5
	13f	Describe any sensitivity analyses conducted to assess robustness of the synthesized results.	Methods, Statistical analysis, page 5
Reporting bias assessment	14	Describe any methods used to assess risk of bias due to missing results in a synthesis (arising from reporting biases).	Methods, Risk of bias assessment and certainty of evidence, page 5
Certainty assessment	15	Describe any methods used to assess certainty (or confidence) in the body of evidence for an outcome.	Methods, Risk of bias assessment and certainty of evidence, page 5
**RESULTS**			
Study selection	16a	Describe the results of the search and selection process, from the number of records identified in the search to the number of studies included in the review, ideally using a flow diagram.	Study selection and characteristics of the included studies/Figure

Section and Topic	Item #	Checklist item	Location where item is reported
			1, page 6
	16b	Cite studies that might appear to meet the inclusion criteria, but which were excluded, and explain why they were excluded.	This part is not included in the manuscript due to the word limit.
Study characteristics	17	Cite each included study and present its characteristics.	Study selection and characteristics of the included studies/Table 1, page 6
Risk of bias in studies	18	Present assessments of risk of bias for each included study.	Table 7
Results of individual studies	19	For all outcomes, present, for each study: (a) summary statistics for each group (where appropriate) and (b) an effect estimate and its precision (e.g. confidence/credible interval), ideally using structured tables or plots.	Table 2, Table 5, supplementary figures
Results of syntheses	20a	For each synthesis, briefly summarise the characteristics and risk of bias among contributing studies.	Risk of bias, page 12, Table 7
	20b	Present results of all statistical syntheses conducted. If meta-analysis was done, present for each the summary estimate and its precision (e.g. confidence/credible interval) and measures of statistical heterogeneity. If comparing groups, describe the direction of the effect.	Meta-analysis, page 6-12
	20c	Present results of all investigations of possible causes of heterogeneity among study results.	Meta-analysis, page 6-12
	20d	Present results of all sensitivity analyses conducted to assess the robustness of the synthesized results.	No sensitivity analyses.
Reporting biases	21	Present assessments of risk of bias due to missing results (arising from reporting biases) for each synthesis assessed.	Publication bias assessments were reported for all analyses in “Meta-analysis” section, page 6-12
Certainty of evidence	22	Present assessments of certainty (or confidence) in the body of evidence for each outcome assessed.	Certainty of evidence was reported for all analyses in “Meta-analysis” section, page 6-12
**DISCUSSION**			

Section and Topic	Item #	Checklist item	Location where item is reported
Discussion	23a	Provide a general interpretation of the results in the context of other evidence.	The first paragraph, page 12-13
	23b	Discuss any limitations of the evidence included in the review.	Limitations, page 16-17
	23c	Discuss any limitations of the review processes used.	Not present
	23d	Discuss implications of the results for practice, policy, and future research.	Conclusion, page 17
**OTHER INFORMATION**			
Registration and protocol	24a	Provide registration information for the review, including register name and registration number, or state that the review was not registered.	Methods, Study design, page 3
	24b	Indicate where the review protocol can be accessed, or state that a protocol was not prepared.	Methods, Study design, page 3
	24c	Describe and explain any amendments to information provided at registration or in the protocol.	Not applicable
Support	25	Describe sources of financial or non-financial support for the review, and the role of the funders or sponsors in the review.	Funding
Competing interests	26	Declare any competing interests of review authors.	Competing interests
Availability of data, code and other materials	27	Report which of the following are publicly available and where they can be found: template data collection forms; data extracted from included studies; data used for all analyses; analytic code; any other materials used in the review.	Data availability statement

Supplementary Table 2: The certainty of evidence regarding the effect of sevoflurane pretreatment and preconditioning on myocardial I/R injury.

Outcome	Number of experiments	Risk of bias	Imprecision	Inconsistency (I^2 range)	Indirectnes s	Publication bias	Level of evidence
**Pre-treatment**							
**Cardiac function**							
HR	4	Not serious	Serious	No serious inconsistency	Not serious	CD	Moderate
**Infarction**							
Infarct size	14	Not serious	No serious imprecision	No serious inconsistency*	Not serious	No publication bias	High
**Preconditioning**							
**Cardiac function**							
-dp/dt	4	Not serious	No serious imprecision	No serious inconsistency	Not serious	CD	High
+dp/dt	4	Not serious	No serious imprecision	Serious	Not serious	CD	High
LVEDP	4	Not serious	No serious imprecision	No serious inconsistency	Not serious	CD	High
LVEF	6	Not serious	No serious imprecision	Serious	Not serious	CD	Moderate
HR	9	Not serious	No serious imprecision	Serious	Not serious	CD	Moderate
**Infarction**							
Infarct size	17	Not serious	No serious imprecision	Serious	Not serious	No publication bias	Moderate

**Apoptosis**							
TUNEL-positive cells	11	Not serious	No serious imprecision	Serious	Not serious	No publication bias	Moderate
Caspase-3	5	Not serious	No serious imprecision	Serious	Not serious	CD	Moderate
**Oxidative stress**							
MDA	5	Not serious	No serious imprecision	Serious	Not serious	CD	Moderate
SOD	5	Not serious	No serious imprecision	Serious	Not serious	CD	Moderate
**Cardiac biomarkers**							
Troponin-I	4	Not serious	No serious imprecision	Serious	Not serious	CD	Moderate
LDH	5	Not serious	No serious imprecision	Serious	Not serious	CD	Moderate

CD: cannot be determined due to insufficient number of experiments; ; HR: Heart rate; -dp/dt: maximal decline in left ventricular pressure; +dp/dt: maximal rate of left ventricular pressure; LVEDP: Left ventricular end-diastolic pressure; LVEF: Left ventricular ejection fraction; MDA: Malondialdehyde; SOD: Superoxide Dismutase; LDH: Lactate dehydrogenase.

*There is no serious inconsistency since the sources of heterogeneity were identified.

Supplementary Table 3: The certainty of evidence regarding the effect of sevoflurane post-conditioning on myocardial I/R injury.

Outcome	Number of experiments	Risk of bias	Imprecision	Inconsistency (I^2 range)	Indirectness	Publication bias	Level of evidence
**Cardiac function**							
-dp/dt	20	Not serious	No serious imprecision	No serious inconsistency*	Not serious	No publication bias	High
+dp/dt	24	Not serious	No serious imprecision	No serious inconsistency*	Not serious	No publication bias	High
LVDP	13	Not serious	No serious imprecision	No serious inconsistency*	Not serious	No publication bias	High
LVEDP	19	Not serious	No serious imprecision	Serious	Not serious	No publication bias	Moderate
LVSP	8	Not serious	No serious imprecision	Serious	Not serious	CD	Moderate
LVIDd	4	Not serious	No serious imprecision	Serious	Not serious	CD	Moderate
LVIDs	4	Not serious	No serious imprecision	No serious inconsistency	Not serious	CD	High
LVEF	7	Not serious	No serious imprecision	Serious	Not serious	CD	Moderate
LVFS	6	Not serious	No serious imprecision	Serious	Not serious	CD	Moderate
HR	28	Not serious	No serious imprecision	No serious inconsistency*	Not serious	No publication bias	High
**Infarction**							
Infarct size	50	Not serious	No serious imprecision	Serious	Not serious	No publication bias	Moderate

**Apoptosis**							
TUNEL positive cells	23	Not serious	No serious imprecision	No serious inconsistency*	Not serious	No publication bias	High
Bcl-2	5	Not serious	No serious imprecision	Serious	Not serious	CD	Moderate
Caspase-3	7	Not serious	No serious imprecision	Serious	Not serious	CD	Moderate
**Inflammation**							
IL-1β	5	Not serious	No serious imprecision	Serious	Not serious	CD	Moderate
IL-6	6	Not serious	No serious imprecision	Serious	Not serious	CD	Moderate
TNF-α	6	Not serious	No serious imprecision	Serious	Not serious	CD	Moderate
**Oxidative stress**							
GSH	5	Not serious	No serious imprecision	Serious	Not serious	CD	Moderate
MDA	10	Not serious	No serious imprecision	No serious inconsistency*	Not serious	No publication bias	High
SOD	9	Not serious	No serious imprecision	Serious	Not serious	CD	Moderate
**Cardiac biomarkers**							
CK-MB	8	Not serious	No serious imprecision	Serious	Not serious	CD	Moderate
Troponin-I	11	Not serious	No serious imprecision	Serious	Not serious	No publication bias	Moderate

LDH	10	Not serious	No serious imprecision	Serious	Not serious	No publication bias	Moderate

CD: cannot be determined due to insufficient number of experiments; -dp/dt: Maximal decline in left ventricular pressure; +dp/dt: Maximal rate of left ventricular pressure; LVDP: Left ventricular diastolic pressure; LVEDP: Left ventricular end-diastolic pressure; LVSP: Left ventricular systolic pressure; LVIDd: Left ventricular internal diameter at end-diastole; LVIDs: Left ventricular internal diameter at end-systole; LVEF: Left ventricular ejection fraction; LVFS: Left ventricular fractional shortening; HR: Heart rate; Bcl-2: B-cell lymphoma 2; IL-1β: Interleukin-1 beta; IL-6: Interleukin-6; TNF-α: Tumor necrosis factor alpha; GSH: Glutathione; MDA: Malondialdehyde; SOD: Superoxide Dismutase; CK-MB: creatine kinase-myocardial band; LDH: Lactate dehydrogenase.

*There is no serious inconsistency since the sources of heterogeneity were identified.

Supplementary material 1: Search strategy

PubMed:

1- “Myocardial Reperfusion Injury”[mh] OR “Myocardial Ischemia”[mh] OR “Acute Coronary Syndrome”[mh] OR “Angina Pectoris”[mh] OR “Angina, Stable”[mh] OR “Angina, Unstable”[mh] OR “Coronary Disease”[mh] OR “Coronary Artery Disease”[mh] OR “Coronary Occlusion”[mh] OR “Coronary Stenosis”[mh] OR “Coronary Thrombosis”[mh] OR “Coronary Vasospasm”[mh] OR “Myocardial Infarction”[mh] OR “Anterior Wall Myocardial Infarction”[mh] OR “Inferior Wall Myocardial Infarction”[mh] OR “Non-ST Elevated Myocardial Infarction”[mh] OR “ST Elevation Myocardial Infarction”[mh] OR Myocardial Reperfusion[tiab] OR Myocardial Ischemi*[tiab] OR Myocardial ischaemi*[tiab] OR Ischemic Heart Diseas*[tiab] OR Acute Coronary Syndrome[tiab] OR Angina Pectoris[tiab] OR Stable Angina[tiab] OR Unstable Angina[tiab] OR Coronary Disease[tiab] OR Disease, Coronary[tiab] OR Coronary Heart Disease[tiab] OR Artery Disease, Coronary[tiab] OR Artery Diseases, Coronary[tiab] OR Coronary Artery Disease[tiab] OR Coronary Arteriosclerosis[tiab] OR Coronary Atherosclerosis[tiab] OR Coronary Occlusion[tiab] OR Coronary Stenosis[tiab] OR Coronary Artery Stenosis[tiab] OR Coronary Thrombosis[tiab] OR Coronary Vasospasm[tiab] OR Coronary Artery Vasospasm[tiab] OR Myocardial Infarc*[tiab] OR Ischem* / reperfusion Injury[tiab] OR Ischem*/reperfusion Injury[tiab] OR Ischemia-reperfusion Injury[tiab] OR Reperfused Acute Myocardial Infarction[tiab] OR Reperfused Myocardial Infarction[tiab] OR cardiac ischaemia reperfusion injury[tiab] OR cardiac ischaemia/reperfusion injury[tiab] OR cardiac ischaemic reperfusion injury[tiab] OR cardiac ischemia reperfusion injury[tiab] OR cardiac ischemia/reperfusion injury[tiab] OR cardiac ischemic reperfusion injury[tiab] OR cardiac reperfusion injury[tiab] OR cardiomyocyte ischaemia-reperfusion injury[tiab] OR cardiomyocyte ischemia-reperfusion injury[tiab] OR heart ischaemia reperfusion injury[tiab] OR heart ischaemia-reperfusion injury[tiab] OR heart ischaemic reperfusion injury[tiab] OR heart ischemia reperfusion injury[tiab] OR heart ischemia-reperfusion injury[tiab] OR heart ischemic reperfusion injury[tiab] OR heart muscle ischemia reperfusion injury[tiab] OR heart muscle ischemic reperfusion injury[tiab] OR heart reperfusion injury[tiab] OR reperfusion cardiac injury[tiab] OR reperfusion heart injury[tiab] OR reperfusion myocardial injury[tiab] OR reperfusion-induced cardiac injury[tiab] OR reperfusion-induced cardiomyocyte injury[tiab] OR reperfusion-induced heart injury[tiab] OR reperfusion-induced myocardial injury[tiab] OR heart muscle ischaemia[tiab] OR heart muscle ischemia[tiab] OR cardiac ischaemia[tiab] OR cardiac ischemia[tiab] OR cardiac muscle ischaemia[tiab] OR cardiac muscle ischemia[tiab] OR coronary artery ischaemia[tiab] OR coronary artery ischemia[tiab] OR coronary ischaemia[tiab] OR coronary ischemia[tiab] OR coronary syndrome[tiab] OR heart ischaemi*[tiab] OR ischaemic heart[tiab] OR ischemic heart[tiab] OR coronary artery insufficiency[tiab] OR coronary artery occlusive disease[tiab] OR coronary insufficiency[tiab] OR coronary occlusive disease[tiab] OR ischaemia heart disease[tiab] OR ischaemic cardiac disease[tiab] OR ischaemic cardial disease[tiab] OR ischaemic cardiopathy[tiab] OR ischaemic heart disease[tiab] OR ischemia heart disease[tiab] OR ischemic cardiac disease[tiab] OR ischemic cardial disease[tiab] OR ischemic cardiopathy[tiab] OR arteriosclerotic cardiac disease[tiab] OR arteriosclerotic heart disease[tiab] OR atherosclerotic heart disease[tiab] OR coronary arteriosclerosis[tiab] OR coronary artery sclerosis[tiab] OR coronary atherosclerosis[tiab] OR coronary cardiosclerosis[tiab] OR coronary sclerosis[tiab] OR heart arteriosclerosis[tiab] OR heart atherosclerosis[tiab] OR heart coronary

sclerosis[tiab] OR coronary artery vasoconstriction[tiab] OR coronary constriction[tiab] OR coronary vasoconstriction[tiab] OR coronary arterial obstruction[tiab] OR coronary artery stenosis[tiab] OR coronary obstruction[tiab] OR acute coronary artery thrombosis[tiab] OR coronary arterial thrombosis[tiab] OR cardiac infarc*[tiab] OR heart infarc*[tiab] OR atrial infarction[tiab] OR auricular infarction[tiab] OR cardiac muscle necrosis[tiab] OR cardiac necrosis[tiab] OR heart fiber necrosis[tiab] OR heart muscle cell necrosis[tiab] OR heart necrosis[tiab] OR myocardial fiber necrosis[tiab] OR myocardial necrosis[tiab]

2- “Sevoflurane”[mh] OR “Sevoflurane “[tiab] OR “Sevorane”[tiab] OR “petrem”[tiab] OR “sevoflo”[tiab] OR “sevofluran”[tiab] OR “sevofrane”[tiab] OR “sevorane”[tiab] OR “sevotec”[tiab] OR “sojourn”[tiab] OR “ultane”[tiab] OR “sevoflurane”[tiab]

3- #1 AND #2

Embase:

1- ‘heart muscle reperfusion’/exp OR ‘myocardial ischemia reperfusion injury’/exp OR ‘heart muscle ischemia’/exp OR ‘Acute Coronary Syndrome’/exp OR ‘ischemic heart disease’/exp OR ‘non st segment elevation acute coronary syndrome’/exp OR ‘stable angina pectoris’/exp OR ‘unstable angina pectoris’/exp OR ‘impending heart infarction’/exp OR ‘impending heart infarction’/exp OR ‘coronary artery spasm’/exp OR ‘coronary artery obstruction’/exp OR ‘coronary artery thrombosis’/exp OR ‘heart infarction’/exp OR ‘acute heart infarction’/exp OR ‘anterior myocardial infarction’/exp OR ‘experimental myocardial infarction’/exp OR ‘heart atrium infarction’/exp OR ‘heart muscle necrosis’/exp OR ‘heart ventricle infarction’/exp OR ‘impending heart infarction’/exp OR ‘inferior myocardial infarction’/exp OR ‘posterior myocardial infarction’/exp OR ‘silent myocardial infarction’/exp OR ‘ST segment elevation myocardial infarction’/exp OR ‘heart left ventricle infarction’/exp OR ‘heart right ventricle infarction’/exp OR ‘coronary artery disease’/exp OR ‘acute coronary syndrome’/exp OR ‘coronary artery atherosclerosi’/exp OR ‘coronary artery calcification’/exp OR ‘coronary artery constriction’/exp OR ‘Myocardial Reperfusion’:ab,ti OR ‘Myocardial Ischemi*’:ab,ti OR ‘Myocardial ischaemi*’:ab,ti OR ‘Ischemic Heart Diseas*’:ab,ti OR ‘Acute Coronary Syndrome’:ab,ti OR ‘Angina Pectoris’:ab,ti OR ‘Stable Angina’:ab,ti OR ‘Unstable Angina’:ab,ti OR ‘Coronary Disease’:ab,ti OR ‘Disease, Coronary’:ab,ti OR ‘Coronary Heart Disease’:ab,ti OR ‘Artery Disease, Coronary’:ab,ti OR ‘Artery Diseases, Coronary’:ab,ti OR ‘Coronary Artery Disease’:ab,ti OR ‘Coronary Arteriosclerosis’:ab,ti OR ‘Coronary Atherosclerosis’:ab,ti OR ‘Coronary Occlusion’:ab,ti OR ‘Coronary Stenosis’:ab,ti OR ‘Coronary Artery Stenosis’:ab,ti OR ‘Coronary Thrombosis’:ab,ti OR ‘Coronary Vasospasm’:ab,ti OR ‘Coronary Artery Vasospasm’:ab,ti OR ‘Myocardial Infarc*’:ab,ti OR ‘Ischem* / reperfusion Injury’:ab,ti OR ‘Ischem*/reperfusion Injury’:ab,ti OR ‘Ischemia-reperfusion Injury’:ab,ti OR ‘Reperfused Acute Myocardial Infarction’:ab,ti OR ‘Reperfused Myocardial Infarction’:ab,ti OR ‘cardiac ischaemia reperfusion injury’:ab,ti OR ‘cardiac ischaemia/reperfusion injury’:ab,ti OR ‘cardiac ischaemic reperfusion injury’:ab,ti OR ‘cardiac ischemia reperfusion injury’:ab,ti OR ‘cardiac ischemia/reperfusion injury’:ab,ti OR ‘cardiac ischemic reperfusion injury’:ab,ti OR ‘cardiac reperfusion injury’:ab,ti OR ‘cardiomyocyte ischaemia-reperfusion injury’:ab,ti OR ‘cardiomyocyte ischemia-reperfusion injury’:ab,ti OR ‘heart ischaemia reperfusion injury’:ab,ti

OR ‘heart ischaemia-reperfusion injury’:ab,ti OR ‘heart ischaemic reperfusion injury’:ab,ti OR ‘heart ischemia reperfusion injury’:ab,ti OR ‘heart ischemia-reperfusion injury’:ab,ti OR ‘heart ischemic reperfusion injury’:ab,ti OR ‘heart muscle ischemia reperfusion injury’:ab,ti OR ‘heart muscle ischemic reperfusion injury’:ab,ti OR ‘heart reperfusion injury’:ab,ti OR ‘reperfusion cardiac injury’:ab,ti OR ‘reperfusion heart injury’:ab,ti OR ‘reperfusion myocardial injury’:ab,ti OR ‘reperfusion-induced cardiac injury’:ab,ti OR ‘reperfusion-induced cardiomyocyte injury’:ab,ti OR ‘reperfusion-induced heart injury’:ab,ti OR ‘reperfusion-induced myocardial injury’:ab,ti OR ‘heart muscle ischaemia’:ab,ti OR ‘heart muscle ischemia’:ab,ti OR ‘cardiac ischaemia’:ab,ti OR ‘cardiac ischemia’:ab,ti OR ‘cardiac muscle ischaemia’:ab,ti OR ‘cardiac muscle ischemia’:ab,ti OR ‘coronary artery ischaemia’:ab,ti OR ‘coronary artery ischemia’:ab,ti OR ‘coronary ischaemia’:ab,ti OR ‘coronary ischemia’:ab,ti OR ‘coronary syndrome’:ab,ti OR ‘heart ischaemi*’:ab,ti OR ‘ischaemic heart’:ab,ti OR ‘ischemic heart’:ab,ti OR ‘coronary artery insufficiency’:ab,ti OR ‘coronary artery occlusive disease’:ab,ti OR ‘coronary insufficiency’:ab,ti OR ‘coronary occlusive disease’:ab,ti OR ‘ischaemia heart disease’:ab,ti OR ‘ischaemic cardiac disease’:ab,ti OR ‘ischaemic cardial disease’:ab,ti OR ‘ischaemic cardiopathy’:ab,ti OR ‘ischaemic heart disease’:ab,ti OR ‘ischemia heart disease’:ab,ti OR ‘ischemic cardiac disease’:ab,ti OR ‘ischemic cardial disease’:ab,ti OR ‘ischemic cardiopathy’:ab,ti OR ‘arteriosclerotic cardiac disease’:ab,ti OR ‘arteriosclerotic heart disease’:ab,ti OR ‘atherosclerotic heart disease’:ab,ti OR ‘coronary arteriosclerosis’:ab,ti OR ‘coronary artery sclerosis’:ab,ti OR ‘coronary atherosclerosis’:ab,ti OR ‘coronary cardiosclerosis’:ab,ti OR ‘coronary sclerosis’:ab,ti OR ‘heart arteriosclerosis’:ab,ti OR ‘heart atherosclerosis’:ab,ti OR ‘heart coronary sclerosis’:ab,ti OR ‘coronary artery vasoconstriction’:ab,ti OR ‘coronary constriction’:ab,ti OR ‘coronary vasoconstriction’:ab,ti OR ‘coronary arterial obstruction’:ab,ti OR ‘coronary artery stenosis’:ab,ti OR ‘coronary obstruction’:ab,ti OR ‘acute coronary artery thrombosis’:ab,ti OR ‘coronary arterial thrombosis’:ab,ti OR ‘cardiac infarc*’:ab,ti OR ‘heart infarc*’:ab,ti OR ‘atrial infarction’:ab,ti OR ‘auricular infarction’:ab,ti OR ‘cardiac muscle necrosis’:ab,ti OR ‘cardiac necrosis’:ab,ti OR ‘heart fiber necrosis’:ab,ti OR ‘heart muscle cell necrosis’:ab,ti OR ‘heart necrosis’:ab,ti OR ‘myocardial fiber necrosis’:ab,ti OR ‘myocardial necrosis’:ab,ti

2- ‘Sevoflurane’/exp OR ‘Sevoflurane‘:ab,ti OR ‘Sevorane’:ab,ti OR ‘propanebax ‘:ab,ti OR ‘petrem’:ab,ti OR ‘sevo-anesteran’:ab,ti OR ‘sevocalm’:ab,ti OR ‘sevoflo’:ab,ti OR ‘sevofluran’:ab,ti OR ‘sevofrane’:ab,ti OR ‘sevohale’:ab,ti OR ‘sevorane’:ab,ti OR ‘sevotec’:ab,ti OR ‘sojourn’:ab,ti OR ‘ultane’:ab,ti OR ‘sevoflurane’:ab,ti

3- #1 AND #2

Scopus:

1- TITLE-ABS-KEY(“Myocardial Reperfusion” OR “Myocardial Ischemi*” OR “Myocardial ischaemi*” OR “Ischemic Heart Diseas*” OR “Acute Coronary Syndrome” OR “Angina Pectoris” OR “Stable Angina” OR “Unstable Angina” OR “Coronary Disease” OR “Disease, Coronary” OR “Coronary Heart Disease” OR “Artery Disease, Coronary” OR “Artery Diseases, Coronary” OR “Coronary Artery Disease” OR “Coronary Arteriosclerosis” OR “Coronary Atherosclerosis” OR “Coronary Occlusion” OR “Coronary Stenosis” OR “Coronary Artery Stenosis” OR “Coronary Thrombosis”

OR “Coronary Vasospasm” OR “Coronary Artery Vasospasm” OR “Myocardial Infarc*” OR “Ischem* / reperfusion Injury” OR “Ischem*/reperfusion Injury” OR “Ischemia-reperfusion Injury” OR “Reperfused Acute Myocardial Infarction” OR “Reperfused Myocardial Infarction” OR “cardiac ischaemia reperfusion injury” OR “cardiac ischaemia/reperfusion injury” OR “cardiac ischaemic reperfusion injury” OR “cardiac ischemia reperfusion injury” OR “cardiac ischemia/reperfusion injury” OR “cardiac ischemic reperfusion injury” OR “cardiac reperfusion injury” OR “cardiomyocyte ischaemia-reperfusion injury” OR “cardiomyocyte ischemia-reperfusion injury” OR “heart ischaemia reperfusion injury” OR “heart ischaemia-reperfusion injury” OR “heart ischaemic reperfusion injury” OR “heart ischemia reperfusion injury” OR “heart ischemia-reperfusion injury” OR “heart ischemic reperfusion injury” OR “heart muscle ischemia reperfusion injury” OR “heart muscle ischemic reperfusion injury” OR “heart reperfusion injury” OR “reperfusion cardiac injury” OR “reperfusion heart injury” OR “reperfusion myocardial injury” OR “reperfusion-induced cardiac injury” OR “reperfusion-induced cardiomyocyte injury” OR “reperfusion-induced heart injury” OR “reperfusion-induced myocardial injury” OR “heart muscle ischaemia” OR “heart muscle ischemia” OR “cardiac ischaemia” OR “cardiac ischemia” OR “cardiac muscle ischaemia” OR “cardiac muscle ischemia” OR “coronary artery ischaemia” OR “coronary artery ischemia” OR “coronary ischaemia” OR “coronary ischemia” OR “coronary syndrome” OR “heart ischaemi*” OR “ischaemic heart” OR “ischemic heart” OR “coronary artery insufficiency” OR “coronary artery occlusive disease” OR “coronary insufficiency” OR “coronary occlusive disease” OR “ischaemia heart disease” OR “ischaemic cardiac disease” OR “ischaemic cardial disease” OR “ischaemic cardiopathy” OR “ischaemic heart disease” OR “ischemia heart disease” OR “ischemic cardiac disease” OR “ischemic cardial disease” OR “ischemic cardiopathy” OR “arteriosclerotic cardiac disease” OR “arteriosclerotic heart disease” OR “atherosclerotic heart disease” OR “coronary arteriosclerosis” OR “coronary artery sclerosis” OR “coronary atherosclerosis” OR “coronary cardiosclerosis” OR “coronary sclerosis” OR “heart arteriosclerosis” OR “heart atherosclerosis” OR “heart coronary sclerosis” OR “coronary artery vasoconstriction” OR “coronary constriction” OR “coronary vasoconstriction” OR “coronary arterial obstruction” OR “coronary artery stenosis” OR “coronary obstruction” OR “acute coronary artery thrombosis” OR “coronary arterial thrombosis” OR “cardiac infarc*” OR “heart infarc*” OR “atrial infarction” OR “auricular infarction” OR “cardiac muscle necrosis” OR “cardiac necrosis” OR “heart fiber necrosis” OR “heart muscle cell necrosis” OR “heart necrosis” OR “myocardial fiber necrosis” OR “myocardial necrosis”)

2- TITLE-ABS-KEY(“Sevoflurane” OR “Sevorane” OR “propanebax” OR “petrem” OR “sevo-anesteran” OR “sevocalm” OR “sevoflo” OR “sevofluran” OR “sevofrane” OR “sevohale” OR “sevorane” OR “sevotec” OR “sojourn” OR “ultane” OR “sevoflurane”)

3- #1 AND #2

Web of Science

1- TS=(“Myocardial Reperfusion” OR “Myocardial Ischemi*” OR “Myocardial ischaemi*” OR “Ischemic Heart Diseas*” OR “Acute Coronary Syndrome” OR “Angina Pectoris” OR “Stable

Angina" OR "Unstable Angina" OR "Coronary Disease" OR "Disease, Coronary" OR "Coronary Heart Disease" OR "Artery Disease, Coronary" OR "Artery Diseases, Coronary" OR "Coronary Artery Disease" OR "Coronary Arteriosclerosis" OR "Coronary Atherosclerosis" OR "Coronary Occlusion" OR "Coronary Stenosis" OR "Coronary Artery Stenosis" OR "Coronary Thrombosis" OR "Coronary Vasospasm" OR "Coronary Artery Vasospasm" OR "Myocardial Infarc*" OR "Ischem* / reperfusion Injury" OR "Ischem*/reperfusion Injury" OR "Ischemia-reperfusion Injury" OR "Reperfused Acute Myocardial Infarction" OR "Reperfused Myocardial Infarction" OR "cardiac ischaemia reperfusion injury" OR "cardiac ischaemia/reperfusion injury" OR "cardiac ischaemic reperfusion injury" OR "cardiac ischemia reperfusion injury" OR "cardiac ischemia/reperfusion injury" OR "cardiac ischemic reperfusion injury" OR "cardiac reperfusion injury" OR "cardiomyocyte ischaemia-reperfusion injury" OR "cardiomyocyte ischemia-reperfusion injury" OR "heart ischaemia reperfusion injury" OR "heart ischaemia-reperfusion injury" OR "heart ischaemic reperfusion injury" OR "heart ischemia reperfusion injury" OR "heart ischemia-reperfusion injury" OR "heart ischemic reperfusion injury" OR "heart muscle ischemia reperfusion injury" OR "heart muscle ischemic reperfusion injury" OR "heart reperfusion injury" OR "reperfusion cardiac injury" OR "reperfusion heart injury" OR "reperfusion myocardial injury" OR "reperfusion-induced cardiac injury" OR "reperfusion-induced cardiomyocyte injury" OR "reperfusion-induced heart injury" OR "reperfusion-induced myocardial injury" OR "heart muscle ischaemia" OR "heart muscle ischemia" OR "cardiac ischaemia" OR "cardiac ischemia" OR "cardiac muscle ischaemia" OR "cardiac muscle ischemia" OR "coronary artery ischaemia" OR "coronary artery ischemia" OR "coronary ischaemia" OR "coronary ischemia" OR "coronary syndrome" OR "heart ischaemi*" OR "ischaemic heart" OR "ischemic heart" OR "coronary artery insufficiency" OR "coronary artery occlusive disease" OR "coronary insufficiency" OR "coronary occlusive disease" OR "ischaemia heart disease" OR "ischaemic cardiac disease" OR "ischaemic cardial disease" OR "ischaemic cardiopathy" OR "ischaemic heart disease" OR "ischemia heart disease" OR "ischemic cardiac disease" OR "ischemic cardial disease" OR "ischemic cardiopathy" OR "arteriosclerotic cardiac disease" OR "arteriosclerotic heart disease" OR "atherosclerotic heart disease" OR "coronary arteriosclerosis" OR "coronary artery sclerosis" OR "coronary atherosclerosis" OR "coronary cardiosclerosis" OR "coronary sclerosis" OR "heart arteriosclerosis" OR "heart atherosclerosis" OR "heart coronary sclerosis" OR "coronary artery vasoconstriction" OR "coronary constriction" OR "coronary vasoconstriction" OR "coronary arterial obstruction" OR "coronary artery stenosis" OR "coronary obstruction" OR "acute coronary artery thrombosis" OR "coronary arterial thrombosis" OR "cardiac infarc*" OR "heart infarc*" OR "atrial infarction" OR "auricular infarction" OR "cardiac muscle necrosis" OR "cardiac necrosis" OR "heart fiber necrosis" OR "heart muscle cell necrosis" OR "heart necrosis" OR "myocardial fiber necrosis" OR "myocardial necrosis")

2- TS= ("Sevoflurane" OR "Sevorane" OR "propanebax" OR "petrem" OR "sevo-anesteran" OR "sevocalm" OR "sevoflo" OR "sevofluran" OR "sevofrane" OR "sevohale" OR "sevorane" OR "sevotec" OR "sojourn" OR "ultane" OR "sevoflurane")

3- #1 AND #2
