# Supplementary material for: Protective effect of sevoflurane on myocardial ischemia-reperfusion injury: a systematic review and meta-analysis
Source: Int J Surg. 2024 Aug 2;110(11):7311–30. doi: 10.1097/JS9.0000000000001975 (PMC11573079; doi:10.1097/JS9.0000000000001975)
Supplement: Supplementary file 2 [file js9-110-7311-s002.pptx]

## Slide 1
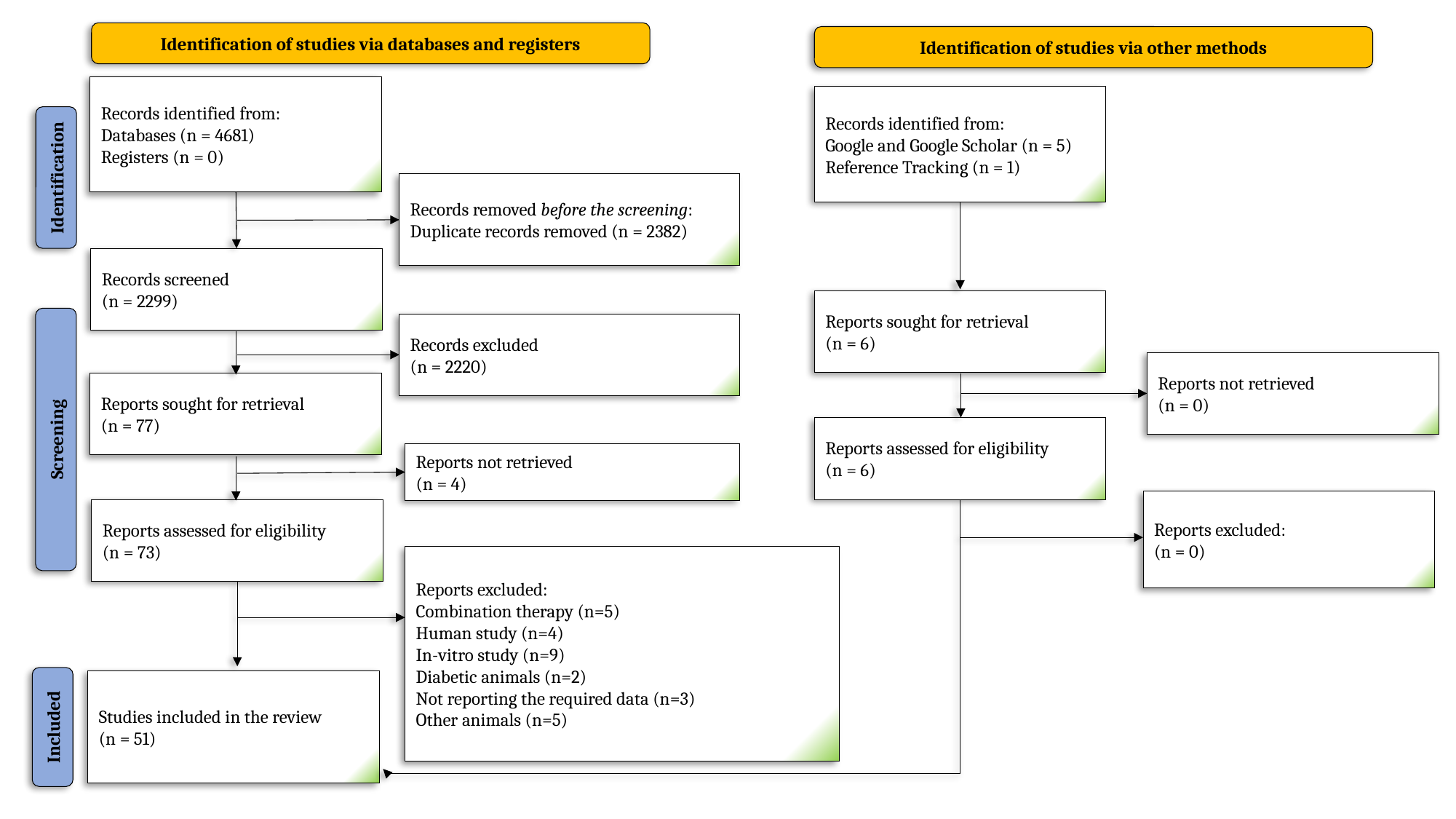

Identification of studies via databases and registers
Identification of studies via other methods
Records identified from:
Databases (n = 4681)
Registers (n = 0)
Records identified from:
Google and Google Scholar (n = 5)
Reference Tracking (n = 1)
Identification
Records removed before the screening:
Duplicate records removed (n = 2382)
Records screened
(n = 2299)
Reports sought for retrieval
(n = 6)
Records excluded
(n = 2220)
Reports not retrieved
(n = 0)
Reports sought for retrieval
(n = 77)
Reports assessed for eligibility
(n = 6)
Screening
Reports not retrieved
(n = 4)
Reports excluded:
(n = 0)
Reports assessed for eligibility
(n = 73)
Reports excluded:
Combination therapy (n=5)
Human study (n=4)
In-vitro study (n=9)
Diabetic animals (n=2)
Not reporting the required data (n=3)
Other animals (n=5)
Studies included in the review
(n = 51)
Included
